# Phylogenomics provides comprehensive insights into the evolutionary relationships among cultivated buckwheat species

**DOI:** 10.1186/s13059-025-03793-2

**Published:** 2025-10-01

**Authors:** Yaliang Shi, Bo Li, Yuanfen Gao, Xiaohan Wang, Yang Liu, Xiang Lu, Hao Lin, Wei Li, Dili Lai, Ming Hao, Jia Gao, Kaixuan Zhang, Dengcai Liu, Sun-Hee Woo, Muriel Quinet, Alisdair R. Fernie, Xu Liu, Yuqi He, Meiliang Zhou

**Affiliations:** 1 National Key Facility for Crop Gene Resources and Genetic Improvement/Key Laboratory of Grain Crop Genetic Resources Evaluation and Utilization, Ministry of Agriculture and Rural Affairs. P. R. China, Institute of Crop Sciences, Chinese Academy of Agricultural Sciences, Beijing, 100081 China; 2 Center for Integrative Conservation, Xishuangbanna Tropical Botanical Garden, Chinese Academy of Sciences, Mengla, 666303 China; 3 Triticeae Research Institute, Sichuan Agricultural University, Chengdu, 610000 China; 4 Department of Crop Science, Chungbuk National University, Cheong-ju, 28644 Republic of Korea; 5 Groupe de Recherche en Physiologie Végétale (GRPV), Earth and Life Institute-Agronomy (ELI-A), Université Catholique de Louvain, Croix du Sud 45, Boîte L7.07.13, B-1348 Louvain-La-Neuve, Belgium; 6 Department of Molecular Physiology, Max-Planck-Institute of Molecular Plant Physiology, Potsdam, 14476 Germany

**Keywords:** Buckwheat, Gene flow, Phylogenetic relationship, Population genetics, Speciation

## Abstract

**Background:**

Buckwheat belongs to the family Polygonaceae and genus *Fagopyrum*, which is characterized by high flavonoid content, short growth period, and strong environmental adaptability. Buckwheat has three cultivated species, including the annual food crops common buckwheat (*Fagopyrum esculentum*) and Tartary buckwheat (*Fagopyrum tataricum*), and the perennial traditional herbal medicine golden buckwheat (*Fagopyrum cymosum*). However, the unclear phylogenetic relationships among these three species based on genomic data limit buckwheat interspecific hybridization and genetic improvement.

**Results:**

Despite their enormous differences in morphology and genome, we confirm the closet relationship between *Fagopyrum cymosum* and *Fagopyrum tataricum*, but not *Fagopyrum esculentum*. The results are also verified through collecting and sequencing an extensive sampling of cultivated/wild populations across all environmentally distinct regions in which these species are found. The changes in flowering time and style morphology controlled by the *AP1* and *S-ELF3* loci significantly contribute to the buckwheat speciation. The introgression from *Fagopyrum cymosum* into wild *Fagopyrum tataricum* explains why wild *Fagopyrum tataricum* exhibits seed morphology similar to *Fagopyrum cymosum*. Furthermore, the convergent traits of leaf morphology and higher flavonoid content between *Fagopyrum cymosum* and wild *Fagopyrum esculentum* are linked to high-altitude adaptation. *Fagopyrum cymosum* is more closely related to wild *Fagopyrum tataricum*, a fact that is confirmed by interspecific hybridization.

**Conclusions:**

Our work provides a valuable example of how phylogenomics can be efficiently utilized for phylogenetic relationship analysis between crops and their wild species relatives, as well as elucidating the plant speciation from the perspectives of genomic evolution and adaptive mechanisms.

**Supplementary Information:**

The online version contains supplementary material available at 10.1186/s13059-025-03793-2.

## Background

Buckwheat, assigned to the family Polygonaceae and genus *Fagopyrum*, is an edible and medical crop plant rich in bioactive phytochemicals that exhibits strong adaptability to the environment and a short growth period, making it a promising solution to address the food crisis, hidden hunger, and anticipated future climate change scenarios [1]. As a result, buckwheat has been recognized as a Future Smart Food in alignment with the United Nations Millennium Development Goals [1]. Recently, buckwheat has attracted increasing attention from agronomic and medical scientists for its various pharmacological properties, including antioxidant, anti-inflammatory, anti-hyperlipidemic, anti-cancer, anti-diabetic, anti-obesity, anti-hypertensive, and hepatoprotective effects [2–5].

The genus *Fagopyrum* Miller comprises 23 known species, primarily endemic to southwest China and the Himalayan region [6–8]. According to morphological and molecular genetic analyses, the genus *Fagopyrum* is divided into two groups. The cymosum group includes three cultivated species, annual grain crops common buckwheat (*Fagopyrum esculentum* Moench) and Tartary buckwheat [*F. tataricum* (Linnaeus) Gaertner], as well as a perennial golden buckwheat [*F. cymosum* (Trevir.) Meisn.] which is utilized as forage and a traditional herbal medicine [9–11]. Until now, the evolutionary relationship among *F. cymosum*, *F. esculentum*, and *F. tataricum* has been a subject of controversy. Both *F. cymosum* and *F. esculentum* exhibit self-incompatibility (SI) due to their heterostyle type of flowers, while *F. tataricum* exhibits self-compatibility (SC) due to its homostyle type of flowers [12]. Additionally, the surface of the achenes is smooth in *F. cymosum* and *F. esculentum*, while it is rough with obvious grooves in *F. tataricum* [12, 13]. Hence, buckwheat scientists, including the eminent Swiss botanist Augustin Pyramus De Candolle, have long considered that *F. cymosum* is closer to *F. esculentum* based on the type of flowers and kernel morphology. Moreover, *F. cymosum* is recognized as the most probable candidate of the primitive type and wild ancestor of *F. esculentum* [13]. However, subsequent phylogenetic analyses based on the chloroplast genomes, microsatellite (SSR) markers, and DNA barcoding indicate a closer genetic relationship between *F. cymosum* and *F. tataricum* [13, 14]. However, the wild progenitor of *F. esculentum*, *F. esculentum* ssp. *ancestrale* Ohnishi is similar to wild *F. cymosum* regarding leaf morphology [15]. The habitats of wild *F. esculentum* closely overlap with *F. cymosum* in southwest China (Yunnan, Sichuan, and partial Tibet), while wild *F. tataricum* closely overlaps with *F. cymosum* in Tibet. Evaluation of phenotypes is confounded by the fact that the habitats of these three species overlap in southwest China and the Himalayan region, which led to them displaying similar characters to one another. Indeed, this may result from various evolutionary mechanisms, including natural hybridization and introgression [15–20]. Recently, comparative genomics analysis revealed that the genome size is similar between *F. cymosum* and *F. tartaricum*, but an estimated genetic divergence time analysis indicated that the divergence between *F. cymosum* and *F. esculentum* occurred before the divergence between *F. cymosum* and *F. tataricum* [11, 21]. Thus, despite extensive studies in this field, the evolutionary relationship among these three species with their wild progenitors remains unclear.

To systematically elucidate the evolutionary relationships among *F. cymosum*, *F. tataricum*, and *F. esculentum*, we not only utilize the genomes of currently cultivars, but also take into account their wild populations from native environments. We collected and sequenced an extensive range of cultivated/wild populations across all environmentally distinct regions in which these species grow. We further generated a high-quality genome assembly for wild *F. esculentum* and conducted comparative phenotypic, genomic, phylogenetic, and population genetic analyses. Phylogenomic and comparative genomic analyses revealed that *F. cymosum* is more closely related to *F. tataricum.* Moreover, gene flow analysis revealed introgression from *F. cymosum* into wild *F. tataricum*, a fact that is consistent with the ease of interspecific (inter-species) hybridization between these species. Additionally, we found that leaf morphology and flavonoid content traits that are convergent between *F. cymosum* and wild *F. esculentum* were found to be linked to high-altitude adaptation. Furthermore, we hypothesized that differences in flowering time between *F. cymosum* and *F. esculentum* as well as the breakdown of self-incompatibility in *F. tataricum* have driven reproductive isolation and speciation. Our work provides comprehensive insights into the genomic evolution, phylogenetic relationships, and adaptive mechanisms underlying *F. cymosum*, *F. tataricum*, and *F. esculentum* phenotypes, offering a valuable example of how phylogenomics can be efficiently utilized for phylogenetic relationship analysis.

## Results

### Characterisation of flower and seed morphology suggests that *F. esculentum* is closer to *F. cymosum* than *F. tataricum*

**Fig. 1:**
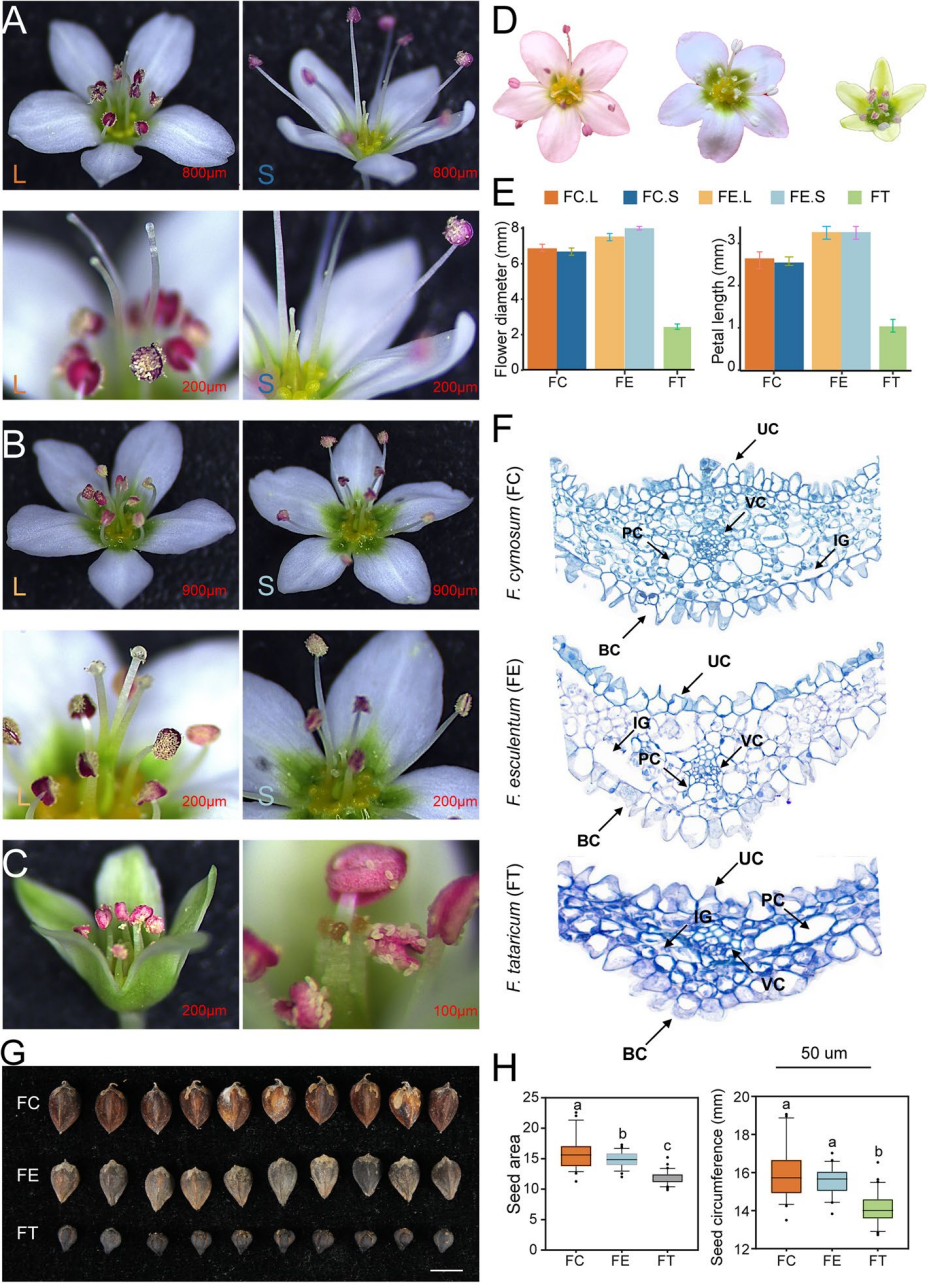
Morphological and size variations in flowers and seeds among *F. cymosum*, *F. esculentum*, and *F. tataricum*. **A** Floral structure of L-morph (long styles and short-level anthers) and S-morph (short styles and long-level anthers) in *F. cymosum*. **B** Floral structures of L-morph and S-morph in *F. esculentum*. **C** Floral structure of homostylous *F. tataricum*. **D, E** Flower size differences among FC (*F. cymosum*), FE (*F. esculentum*), and FT (*F. tataricum*). **F** Longitudinal sections of flowers in *F. cymosum*, *F. esculentum*, and *F. tataricum*. BC, basal epidermal cells; PC, parenchyma cells; UC, upper epidermal cells; VC, vascular cells; IG, intercellular gaps. **G, H** Seed area and perimeter comparisons among *F. cymosum*, *F. esculentum*, and *F. tataricum*. Different letters indicate significant differences at *P* < 0.05 (*t*-test)

To analyze the evolutionary relationships among *F. cymosum* (FC), *F. esculentum* (FE), and *F. tataricum* (FT) from a morphological perspective, we examined the flower and seed morphology of the three species. In all three species, normally developed flowers are comprised of five petals, stamens arranged in two whorls (with three in the inner whorl and five in the outer whorl), and three stigmas. *F. cymosum* produces white and pink flowers, *F. esculentum* features white, pink, purple, and green flowers, while *F. tataricum* bears green flowers exclusively. Both *F. cymosum* and *F. esculentum* exhibit heterostyly (Fig. 1A, B; Additional file 1: Fig. S1), whereas *F. tataricum* displays homostyly (Fig. 1C). The flowers of *F. cymosum* and *F. esculentum* are relatively larger than those of *F. tataricum* (Fig. 1D, E). To explore the underlying reasons for the differences in flower size among the three species, we analyzed the number and size of cells in each tissue by examining their longitudinal sections (Additional file 1: Fig. S2). The results revealed that *F. tataricum* has smaller intercellular gaps and more densely arranged cells. This may explain why *F. tataricum* flowers are smaller than those of *F. cymosum* and *F. esculentum* (Fig. 1F). The seeds of *F. tataricum* are conical with a ventral groove and lack distinct ridges, whereas those of *F. cymosum* and *F. esculentum* are three-angled (Fig. 1G; Additional file 1: Fig. S3). Student’s *t*-tests on seed area and circumference showed that, compared with *F. cymosum* (seed area: 16.023; seed circumference: 15.973) and *F. esculentum* (seed area: 14.836; seed circumference: 15.541), the seeds of *F. tataricum* (seed area: 11.809; seed circumference: 14.155) were significantly smaller (*P* < 0.0001) (Additional file 2: Table S1). By contrast, there was no significant difference between *F. cymosum* and *F. esculentum* (Fig. 1H). Upon examining the buckwheat species’ flower and seed morphological characteristics of the three buckwheat species, we postulated that *F. esculentum* is more like a sister species of *F. cymosum*.

### Comparative genomic and phylogenomic analysis among *F. cymosum*, *F. tataricum*, and *F. esculentum*

**Fig. 2:**
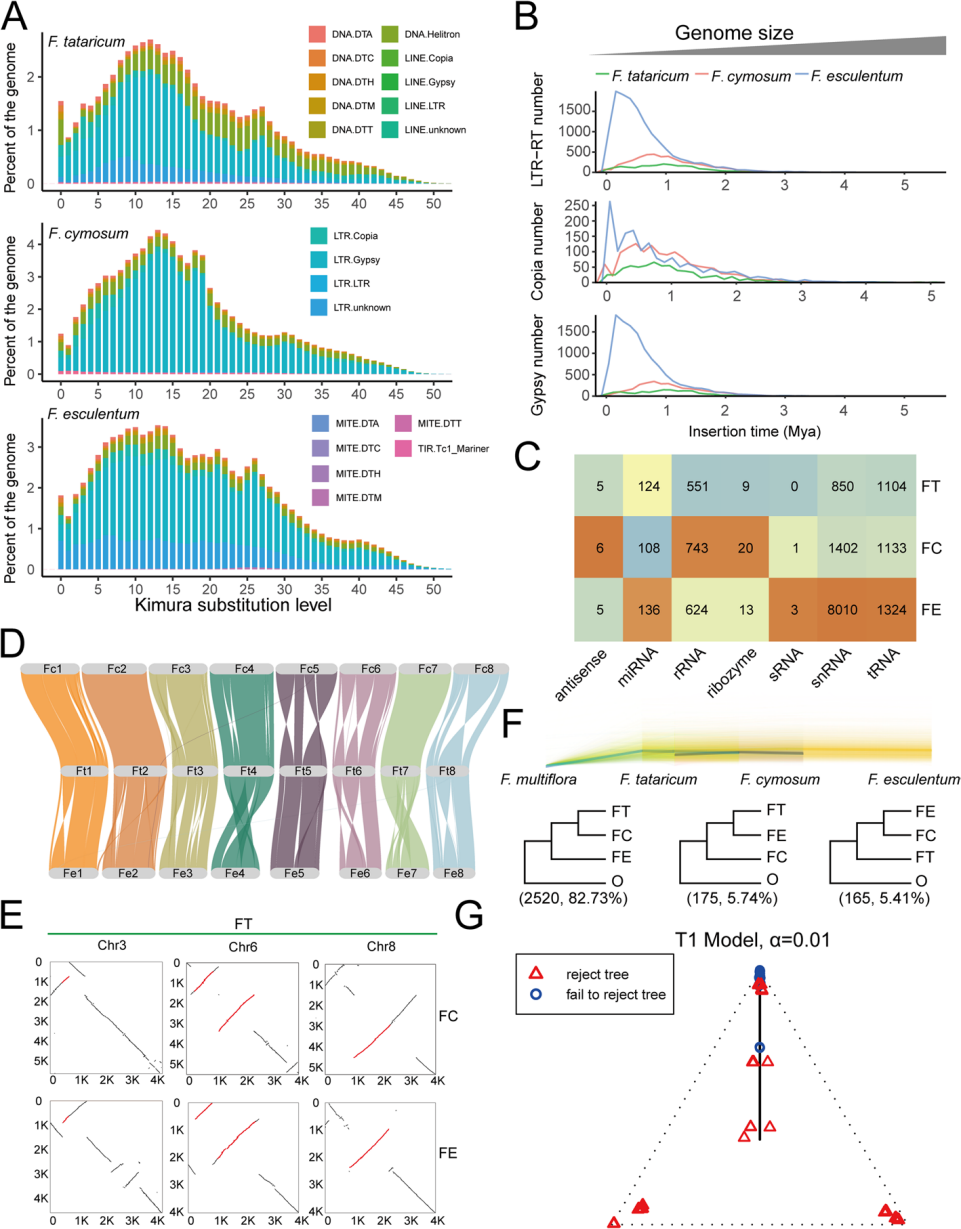
Comparative genomic analysis among *F. cymosum*, *F. tataricum*, and *F. esculentum*. **A** Transposon landscape of *F. cymosum*, *F. tataricum*, and *F. esculentum* genomes. Repeat types are represented by colored bars. **B** Estimated transposon burst times in the genomes of *F. cymosum*, *F. tataricum*, and *F. esculentum*. **C** The types and numbers of non-coding genes in *F. cymosum*, *F. tataricum*, and *F. esculentum* genomes. **D** Syntenic patterns among the genomes of *F. cymosum*, *F. tataricum*, and *F. esculentum* genomes. **E** Collinearity dot plots of three chromosomes among *F. cymosum*, *F. tataricum*, and *F. esculentum* genomes. **F** A total of 1121 single-copy genes were selected for constructing phylogenetic trees among *F. cymosum*, *F. tataricum*, and *F. esculentum*, and the outgroup (*Fallopia multiflora*). **G** Quartet hypothesis test results on the impact of incomplete lineage sorting on the phylogenetic relationships among *F. cymosum*, *F. tataricum*, and *F. esculentum*

To investigate the relationships among *F. cymosum*, *F. tataricum*, and *F. esculentum* at the genomic level, we next analyzed the three reported genomes (*F. cymosum_*LJS genome, *F. tataricum_*HERA genome, and *F. esculentum_*Pintian genome) available in the Buckwheat Genome Database [22–24]. The genome sizes of *F. cymosum* (~ 1.08 Gb) and *F. esculentum* (~ 1.16 Gb) are similar and both are considerably larger than that of *F. tataricum* (~ 0.47 Gb). The three species are diploid, each possessing 16 chromosomes, and there is no significant difference in the number of annotated protein-coding genes among them (Additional file 2: Table S2). To investigate the reasons for the difference in genome size, we first analyzed the genome duplication and repeat sequence content. Furthermore, we did not detect any significant whole-genome duplication events following species divergence (Additional file 1: Fig. S4). Additionally, the number of genes resulting from whole-genome duplication, dispersed duplication, proximal duplication, tandem duplication, and transposed duplication types did not show a significant correlation with the genome size of the three species (Additional file 1: Fig. S5; Additional file 2: Table S2). We subsequently compiled intact transposon libraries for *F. cymosum*, *F. tataricum*, and *F. esculentum* genomes and annotated them by identifying characteristic terminal sequences to distinguish different repeat types (Additional file 2: Table S3). First, the proportion of transposable elements (TEs) in the genomes of *F. cymosum*, *F. tataricum*, and *F. esculentum* exceeds 50%, accounting for 73.98%, 51.55%, and 77.21% of the genomes, respectively. According to the neutral theory of molecular evolution, the accumulation of mutations is time-dependent [25, 26]. Therefore, the distribution of Kimura substitution level reflects the relative transposon insertion time and cumulative amount in the genome. Comparison of TE compositions among the three genomes revealed that the difference in genome size is primarily attributed to a recent large-scale insertion of LTR (Long Terminal Repeats) elements (Fig. 2A; Additional file 2: Table S4). Among the LTR-Gypsy elements, the proportion of LTR-Gypsy elements in *F. cymosum* and *F. esculentum* was similar, accounting for 49.19 and 43.29%, respectively, whereas *F. tataricum* had a significantly lower proportion at 24.33%. Estimates of transposon burst times indicated that the activation periods of various transposons in all three species occurred after their divergence, within the past 1 Mya (Fig. 2B; Additional file 2: Table S5). Therefore, the transposon burst events in *F. cymosum*, *F. tataricum*, and *F. esculentum* occurred independently of one another. In addition, a greater number of LTR insertions were observed in *F. esculentum*, with the lowest number of LTR insertions being observed in *F. tataricum*. Although the genome size of *F. esculentum* is similar to that of *F. cymosum*, its flavonoid content is significantly lower than that of *F. cymosum* and *F. tataricum* [11, 23, 27]. It was found that *F. cymosum* and *F. esculentum* possess more genes than *F. tataricum*, with *F. cymosum* containing a higher proportion of multicopy genes (Additional file 1: Fig. S6; Additional file 2: Table S6). Therefore, to investigate whether multicopy genes contributed to a change in the number of putative enzyme-encoding genes of the main flavonoid metabolism pathway, we identified and compared the copy numbers of flavonoid pathway enzyme genes. We found that the copy number of the phenylalanine ammonia-lyase (*PAL*), cinnamate-4-hydroxylase (*C4H*), 4-coumarate: CoA ligase (*4CL*), chalcone isomerase (*CHI*), flavonoid 3',5'-hydroxylase (*F3′5'H*), and flavonoid 3-O-glucosyltransferase (*F3GT*) gene in *F. esculentum* are lower than in *F. cymosum* and *F. tataricum* (Additional file 1: Fig. S7; Additional file 2: Table S7). This result indicated that the lower copy numbers of some important genes can lead to a reduction of flavonoid content. This observation was interesting given that *F. esculentum* has the largest genome of the three species.

We also subsequently conducted a genome-wide survey of various non-coding genes in *F. cymosum*, *F. tataricum*, and *F. esculentum*. We found that the *F. esculentum* genome contains the highest number of non-coding genes (10,115), followed by *F. cymosum* (3413), with *F. tataricum* having the fewest (2643) (Additional file 2: Table S8). The number of snRNAs in *F. esculentum* (8010) is significantly higher than that in *F. cymosum* (1402) and *F. tataricum* (1104) (Fig. 2C; Additional file 1: Fig. S8). We speculated that a significant expansion of non-coding genes also led to an increase in genome size in *F. esculentum* and *F. cymosum*, although it is important to note that the expansion of non-coding genes in *F. cymosum* is relatively small. Genomic synteny analysis among the three species revealed extensive structural variations, including inversions and translocations among the genomes of *F. cymosum*, *F. tataricum*, and *F. esculentum*; however, the arrangement of genes in *F. tataricum* is similar to that in *F. cymosum* (Fig. 2D). Inversions of chromosomes can play a crucial role in species evolution and adaptation by influencing the recombination dynamics of regional genes, leading to independent genomic evolution between derived and ancestral arrangements, thereby providing opportunities for differentiation and speciation [28, 29]. Four specific inversions in *F. tataricum* were identified on chromosomes 3, 6, and 8 (Fig. 2E; Additional file 2: Table S9). Of the genes in these inversion regions, we found three homologs of *APETALA2* (*AP2*) and *ULTRAPETALA 1* (*ULT1*) genes, which are involved in floral organ development. Kyoto Encyclopedia of Genes and Genomes (KEGG) enrichment analysis revealed that the specific inversion regions in *F. tataricum* include genes related to flavone, flavonol, and anthocyanin biosynthesis, including *PAL*, flavanone-3’-hydroxylase (*F3’H*), and Anthocyanidin-3-O-glucoside rhamnosyltransferase (*RT*) gene (Additional file 1: Fig. S9; Additional file 2: Table S9). We, therefore, speculated that these inversions are functionally important structural variations and may contribute to the differentiation and speciation between *F. cymosum* and *F. tataricum*. To obtain reliable species of phylogenetic trees, a total of 3046 single-copy genes were identified across the four genomes, including the outgroup *Fallopia multiflora* (Fig. 2F; Additional file 2: Table S10). Phylogenetic trees constructed using single-copy genes showed that 82.73% of these support a closer phylogenetic relationship between *F. cymosum* and *F. tataricum*. Given the potential presence of incomplete lineage sorting among the *F. cymosum*, *F. tataricum*, and *F. esculentum* populations, it is necessary to assess whether incomplete lineage sorting has influenced their phylogenetic relationships. Therefore, a quadripartite test was conducted based on the multispecies coalescent model (Additional file 1: Fig. S10). However, the results of the quadripartite test indicated that incomplete lineage sorting had a minimal impact on the phylogenetic relationships among *F. cymosum*, *F. tataricum*, and *F. esculentum* (Fig. 2G). However, the test confirmed a sister relationship between *F. cymosum* and *F. tataricum*. In summary, genomic evidence supports a closer phylogenetic relationship between *F. cymosum* and *F. tataricum* and reveals that the larger genome sizes of *F. cymosum* and *F. esculentum* are attributed to independent transposon bursts.

### Genetic basis of reproductive isolation and species divergence among *F. cymosum*, *F. tataricum*, and *F. esculentum*

**Fig. 3:**
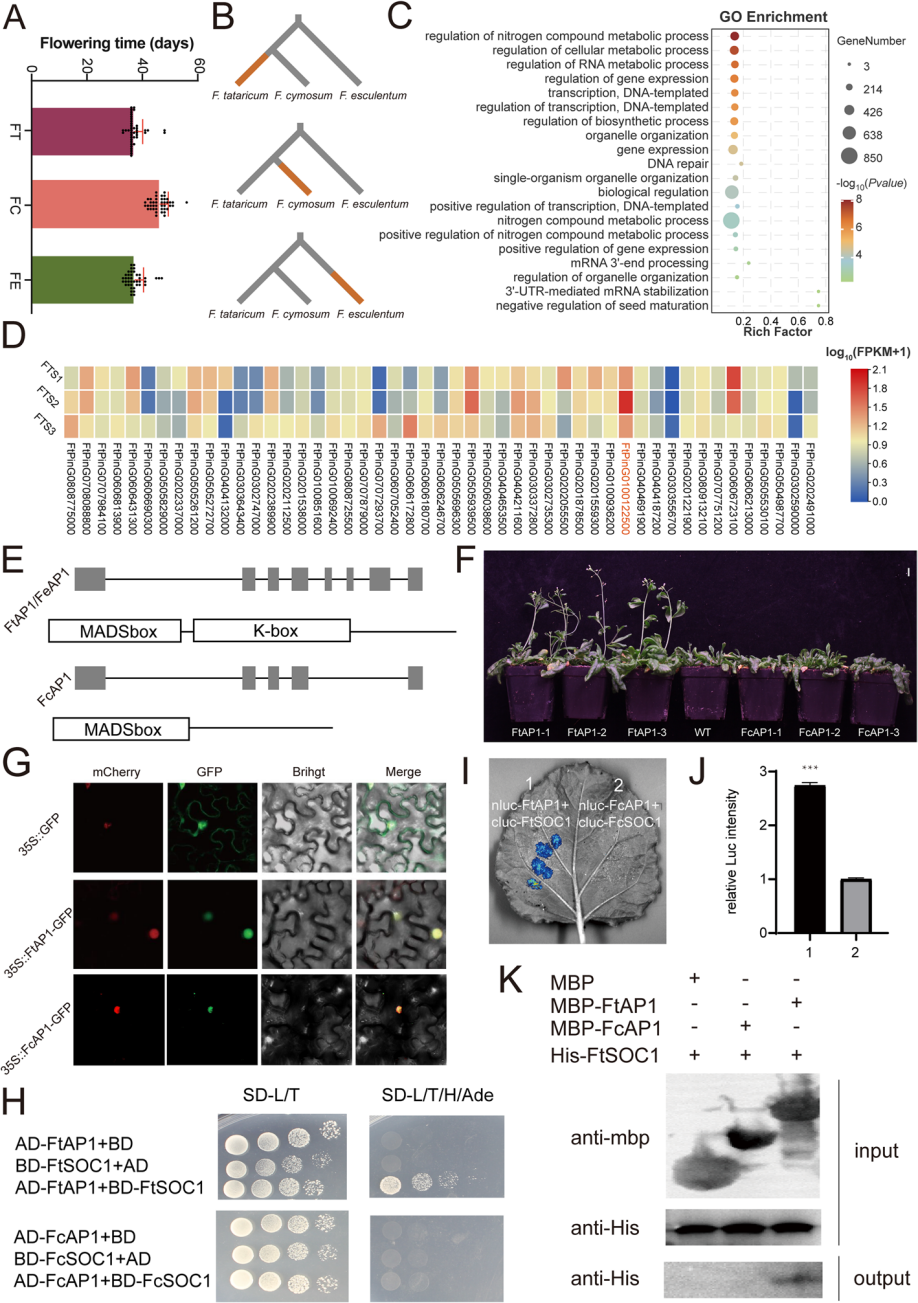
The selection of *AP1* alleles in *F. cymosum* may have contributed to speciation. **A** Flowering time in *F. cymosum*, *F. tataricum*, and *F. esculentum*. **B** Number of positively selected genes in *F. cymosum*, *F. tataricum*, and *F. esculentum*. **C** GO enrichment analysis of positively selected genes in *F. cymosum*. **D** Transcriptomic analysis of *F. tataricum* at different flowering stages. **E** Structural analysis of the *AP1* gene in *F. cymosum*, *F. tataricum*, and *F. esculentum*. **F** Phenotypic analysis (flowering time) in transgenic *Arabidopsis* overexpressing *AP1* from *F. cymosum* and *F. tataricum*. **G** Subcellular localization of FtAP1 and FcAP1. **H** Yeast two-hybrid interaction assay for FtAP1 and FcAP1. **I, J** Luciferase complementation assay (LCA) for FtAP1 and FcAP1. **K** In vitro interaction assay of FtAP1 and FcAP1. In vitro interaction assay of FtAP1-mbp and FcAP1-mbp. FtAP1-mbp and FcAP1-mbp were tested for interactions with FtSOC1-his as the experimental group, while the interaction between the mbp-tag protein and FtSOC1-his served as the control group

The center of origin and habitat range of the different buckwheat species largely overlap, yet the reasons underlying the formation of three distinct species remain unclear. Previous studies have reported that flowering time is a critical factor contributing to reproductive isolation among species [30, 31]. Our investigation confirms significant differences in flowering times among buckwheat species. Specifically, *F. cymosum* exhibited the latest flowering time, with an average of 42.26 days, whereas *F. tataricum* and *F. esculentum* had similar flowering initiation times, averaging 37.16 and 37.69 days, respectively (Fig. 3A; Additional file 2: Table S11). To investigate the genetic basis underlying the differences in flowering time among the three buckwheat species, we examined whether their genes have been subjected to positive selection during evolution. A total of 10,989 single-copy genes were analyzed for positive selection using the branch-site model in PAML, with each of the three evolutionary lineages set as foreground branches (Fig. 3B). The analysis identified 791 positively selected genes in *F. tataricum*, 1389 in *F. esculentum*, and 2795 in *F. cymosum* (Additional file 2: Table S12). These positively selected genes in *F. tataricum* were enriched in biological processes related to environmental adaptation, such as response to UV-B, while genes that were positively selected in *F. esculentum* were enriched in metabolic processes (Additional file 1: Figs. S11 and S12). Subsequent GO enrichment analysis of the genes in *F. cymosum* revealed significant enrichment in pathways related to metabolic regulation and gene expression (Fig. 3C). These findings suggest that these genes may play a crucial role in regulating plant growth and development. Among the positively selected genes in *F. cymosum*, 49 were identified as flowering-related (Additional file 2: Table S13). We analyzed the expression patterns of these genes across the early bud stage (FTS1), mid-bud stage (FTS2), and blooming day (FTS3) in *F. tataricum*. The results showed that *FtPinG010012500* (*FtAP1*) exhibited consistently higher expression levels across all three stages compared to other genes (Fig. 3D). *AP1* is a key gene in the ABCE model and has also been reported as an essential component of the flowering regulatory network [32, 33]. *AP1* transcript levels were also found to be highly expressed in the flowers of two species based on the expression pattern analysis of previous RNA-seq results (Additional file 1: Fig. S13) [23]. This suggests that buckwheat *FcAP1*/*FtAP1* may play a significant role in regulating flowering. A deletion was found at the C-terminus of this gene in *F. cymosum*, while the gene structure remained intact in *F. tataricum* and *F. esculentum*. We hypothesized that this structural alteration may contribute to the later flowering time observed in *F. cymosum* (Fig. 3E). The buckwheat *AP1* genes show homology with AP1 in *Arabidopsis* and other plants, suggesting that they may have similar functions (Additional file 1: Fig. S14). To verify their function, the *FcAP1* and *FtAP1* genes were cloned and transformed into *Arabidopsis thaliana*, and the flowering time of the resultant transgenics was scored and compared to one another. The results showed that the *FtAP1* gene promoted earlier flowering in *Arabidopsis* compared to the *FcAP1* gene (Fig. 3F; Additional file 1: Fig. S15). Subcellular localization analysis revealed that both transcription factors are localized in the nucleus (Fig. 3G). Previous studies have shown that AP1 interacts with SOC1 to co-regulate plant flowering [34]. Through cluster analysis, we found that *FtAP1* and *FtSOC1* exhibited the same expression pattern (Additional file 1: Fig. S16; Additional file 2: Table S14). To investigate whether FtAP1 and FcAP1 interact with the buckwheat SOC1 protein, we next performed yeast two-hybrid assays. The results indicated that FtAP1 interacts with FtSOC1, while FcAP1 does not interact with FcSOC1 (Fig. 3H). The SOC1 genes in *F. tataricum* and *F. cymosum* share a highly consistent sequence, suggesting that they have conserved function (Additional file 1: Fig. S17). To confirm this, we conducted LCA and pull-down assays, which also revealed that only FtAP1 interacts with FtSOC1 (Fig. 3H, I, J, K). Additionally, FtAP1 was found to interact with AtSOC1 from *A. thaliana*, while FcAP1 did not (Additional file 1: Figs. S18, S19). This suggests that the interaction between AP1 and SOC1 in regulating flowering is conserved across plant species. In summary, the differences in flowering time between *F. cymosum* and *F. esculentum* or *F. tataricum* are likely attributed to structural differences in the *AP1* gene, which may have led to reproductive isolation and the early divergence of the two species.

We subsequently investigated the fundamental causes of differences in pollination mechanisms and style morphology between *F. cymosum* and *F. tataricum*. The S-locus has been reported to be closely associated with self-incompatibility [11]. A deletion variant of the *S-ELF3* gene in *F. esculentum* has been found to result in changes in style morphology and self-incompatibility [11, 35]. We also identified that the *S-ELF3* gene on chromosome 1 of *F. cymosum* exhibits the same intact structure and normal expression as in *F. esculentum*. However, in *F. tataricum*, the *S-ELF3* gene on chromosome 7 has undergone a transposon insertion and mutation, transforming it into a pseudogene (Additional file 1: Fig. S20). These differences may potentially lead to the breakdown of self-incompatibility [36]. Therefore, the speciation of *F. cymosum* and *F. tataricum* may be attributed not only to changes in flowering time but also to alterations in style morphology and other reproductive mechanisms.

### Convergent high-altitude adaptive traits between wild *F. esculentum* and wild *F. cymosum*

**Fig. 4:**
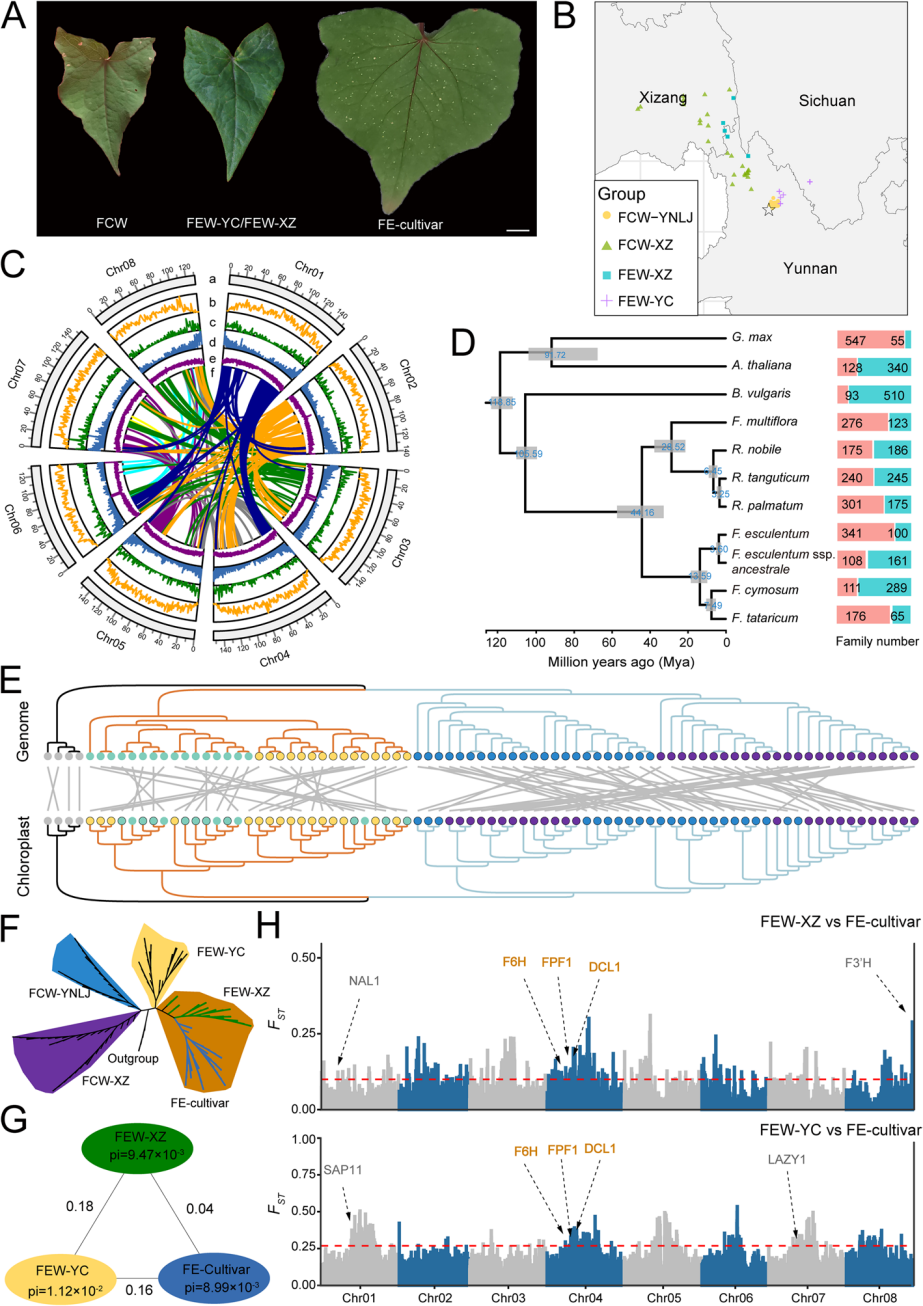
Population genetic and morphology analysis of *F. cymosum* and wild *F. esculentum*. **A** *F. cymosum* (FCW) and wild/cultivated *F. esculentum* leaf morphology. **B** The geographic distribution of wild *F. cymosum* (FCW-YNLJ and FCW) and wild *F. esculentum* (FEW-XZ and FEW-YC). **C** Circos plot of wild *F. esculentum* ssp. *ancestrale* genome. *a* Chromosome size; *b* Gene density; *c* Distribution of LTR-Copia elements; *d* Distribution of LTR-Gypsy elements; *e* GC content distribution; *f* Syntenic relationships within the genome. **D** Divergence time estimation and gene family expansion and contraction. The numbers on the branches of the phylogenetic tree (left) represent divergence times (Mya). The numbers on the right indicate the number of expanded and contracted gene families, respectively. *G. max*, *Glycine max*; *A. thaliana*, *Arabidopsis thaliana*; *B. vulgaris*, *Berberis vulgaris*; *F. multiflora*, *Fallopia multiflora*; *R. nobile*, *Rheum nobile*; *R. tanguticum*, *Rheum tanguticum*; *R. palmatum*, *Rheum palmatum*. **E** Phylogenetic trees of wild *F. cymosum* and wild *F. esculentum* were constructed using nuclear genome data and chloroplast genome data, respectively. The black, yellow, and blue branches represent outgroup (*F. qiangcai*, *F. rubifolium*, *F. gracilipedoides*, and *F. caudatum*), wild *F. esculentum*, and *F. cymosum*, respectively. The connecting lines between the two trees indicate the positions of the same germplasm in each tree.** F** Phylogenetic tree including *F. cymosum* (FCW/FCW-YNLJ), wild *F. esculentum* (FEW-YC/FEW-XZ), and cultivated *F. esculentum* (FE-cultivar). **G** The genetic differentiation index between the two wild *F. esculentum* groups and the cultivated *F. esculentum* group, along with their respective genetic diversity indices (Pi). **H** Genome-wide sliding window genetic differentiation analysis between each of the two wild *F. esculentum* groups and the cultivated *F. esculentum* group. Key genes associated with leaf morphology and flavonoid metabolite biosynthesis were identified within the top 5% differentiation regions

The comparative genomic analysis described above indicates a closer relationship between *F. cymosum* and *F. tataricum*. However, it has been reported that both wild *F. esculentum* (*F. esculentum* ssp. *ancestrale*) and wild *F. cymosum* (FCW) exhibit leaf morphology similarities and higher flavonoid content compared to cultivated *F. esculentum* [15]. The leaf morphology of wild *F. esculentum* and *F. cymosum* exhibits undulate margins and palmate venation, and both leaf sizes are smaller than the cordate leaves of cultivated *F. esculentum* (Fig. 4A; Additional file 1: Fig. S21). Small leaf size and high flavonoid content are traits commonly selected by many plants as adaptations to high-altitude environments [37–42]. The habitats of *F. cymosum* and wild *F. esculentum* overlap geographically. Therefore, we speculated that wild *F. esculentum* was likely close to *F. cymosum* and that interspecific hybridization had occurred. To test this hypothesis and investigate the phylogenetic relationships between *F. cymosum* and wild/cultivated *F. esculentum*, we set out to collect 29 wild *F. esculentum* and 62 wild *F. cymosum* accessions from the Qinghai-Tibet Plateau and the Yunnan-Guizhou Plateau (Fig. 4B; Additional file 2: Table S15). Based on their collection locations. *F. cymosum* was divided into wild *F. cymosum* (FCW) and Lijiang (Yunnan) *F. cymosum* (FCW-YNLJ) groups. Similarly, wild *F. esculentum* was classified into Tibetan wild *F. esculentum* (FEW-XZ) and Yunnan wild *F. esculentum* (FEW-YC) groups. We first assembled a genome of *F. esculentum* ssp. *ancestrale* (DDX) (Fig. 4C). Using 37.05 Gb of PacBio reads, we generated an initial genome assembly of 1.28 Gb, comprising 1093 contigs with an N50 of 127.62 Mb (Additional file 2: Table S16). Using Hi-C data, these contigs were anchored to eight pseudochromosomes, resulting in a total genome assembly size of 1.17 Gb, which included 42 contigs, with an N50 of 55.25 Mb and the longest contig measuring 131.09 Mb (Additional file 1: Fig. S22). Genome quality and completeness were evaluated using Benchmarking Universal Single-Copy Orthologs (BUSCO). The results detected 98.1% completely conserved orthologs, indicating the high completeness of the genome assembly. Additionally, the Long Terminal Repeats (LTR) Assembly Index (LAI) score of 21.74 was higher than 15.40 and 17.65 of the previous genomes, suggesting that the genome quality surpasses previously assembled *F. esculentum* genomes (Additional file 2: Table S17) [24, 35]. A total of 35,332 protein-coding genes were annotated and exhibited 96.7% BUSCO completeness. LTR-RTs accounted for 925.16 Mb, representing 78.85% of the whole genome. The largest LTR-RT superfamilies are *Gypsy*, accounting for 488.37 Mb (41.62% of the genome), and *Copia*, accounting for 61.45 Mb (5.24% of the genome). Therefore, our assembled genome is of high quality and suitable for use as a reference genome in future studies.

To elucidate the phylogenetic relationships among wild/cultivated *F. esculentum* and *F. cymosum*, we constructed a phylogenetic tree using single-copy orthologous genes and estimated divergence times. The results indicated that *F. cymosum* and *F. esculentum* diverged approximately 13.59 Mya, and *F. esculentum* ssp. *ancestrale* and *F. esculentum* approximately 3.60 Mya (Fig. 4D). Gene family expansion facilitates adaptation to new environmental changes [43–46]. *F. esculentum* ssp. *ancestrale* showed 108 expanded and 161 contracted gene families, respectively (Additional file 2: Table S18). A substantial expansion of gene families associated with cell wall thickening and defense was observed in the genome of *F. esculentum* ssp. *ancestrale* (Additional file 1: Fig. S23), which may have enhanced its environmental adaptability [47, 48].

We next performed genome resequencing at an average depth of 10 × for all collected wild *F. esculentum* and wild *F. cymosum* samples, producing a total of 209,518,621 SNPs (Additional file 1: Fig. S24; Additional file 2: Table S19). In addition, we assembled the chloroplast genome of 83 individuals, including *F. cymosum*, cultivated/wild *F. esculentum*, and the outgroup (*F. qiangcai*, *F. rubifolium*, *F. gracilipedoides*, and *F. caudatum*). The chloroplast genome sizes of *F. cymosum*, cultivated/wild *F. esculentum* and the outgroup were around 159 kb in size, and each contained about 122 genes (Additional file 2: Table S20). A phylogenetic tree was constructed using 50 single-copy genes from these chloroplast genomes. Based on the neighbor-joining tree using chloroplast genome sequences and nuclear genome SNPs, which all clearly distinguish wild *F. cymosum* and wild *F. esculentum* (Fig. 4E). Our further ABBA-BABA analysis identified no gene introgression between interspecific groups (Additional file 2: Table S19). Population structure analysis divided *F. cymosum*, and wild/cultivated *F. esculentum* into five groups, consistent with geographic distribution, dividing wild *F. cymosum* into Yunnan and Tibetan groups, and wild *F. esculentum* into Yunnan and Tibetan groups (Additional file 1: Fig. S25), suggesting that cultivated *F. esculentum* diverged from Tibetan wild *F. esculentum* (Fig. 4F). In the group of *F. esculentum*, the genetic differentiation index (*F*_*ST*_) indicates low differentiation (0.04) between cultivated *F. esculentum* and Tibetan wild *F. esculentum*, but moderate differentiation from Yunnan wild *F. esculentum* (Fig. 4G). Besides, cultivated *F. esculentum* exhibits the lowest genetic diversity and linkage disequilibrium (Additional file 1: Fig. S26).

Altitude is the most influential factor affecting leaf size, with leaf area decreasing as altitude increases. Reduced leaf area is considered an adaptive trait for high-altitude environments [40, 42, 49, 50]. Flavonoids are known to act as potent antioxidants by reducing DNA damage caused by high-altitude environments. As such, the accumulation of flavonoids is also considered an adaptive trait for high-altitude environments [38, 41, 42, 51, 52]. By scanning the highly differentiated genomic regions between cultivated *F. esculentum* and the two groups of wild *F. esculentum* from Tibet and Yunnan, we identified genes associated with leaf morphology, specifically Flowering-Promoting Factor 1 (*FPF1*) and Endoribonuclease Dicer Homolog 1 (*DCL1*), as well as Flavonoid-6-Hydroxylase (*F6H*), which is linked to flavonoid content (Fig. 4H; Additional file 2: Table S21). Additionally, high genetic differentiation was detected in the Narrow leaf 1 (*NAL1*) gene, associated with leaf morphology, and the flavonoid 3'-hydroxylase (*F3'H*) gene, related to flavonoid content, between Tibetan wild *F. esculentum* and cultivated *F. esculentum* [53, 54]. The secreted AY-WB protein 11 (*SAP11*) gene, associated with reduced leaf size, showed significant genetic differentiation between Yunnan wild *F. esculentum* and cultivated *F. esculentum* [55]. This suggests that Yunnan wild *F. esculentum* and Tibetan wild *F. esculentum* adapted to high-altitude environments by selecting traits such as smaller leaf size and higher flavonoid content. In summary, despite their relatively distant phylogenetic relationship, *F. cymosum* and wild *F. esculentum* independently selected distinct gene combinations to adapt to high-altitude environments, resulting in similar adaptive phenotypes.

### Introgression and interspecific hybridization between wild *F. cymosum* and wild *F. tataricum*

**Fig. 5:**
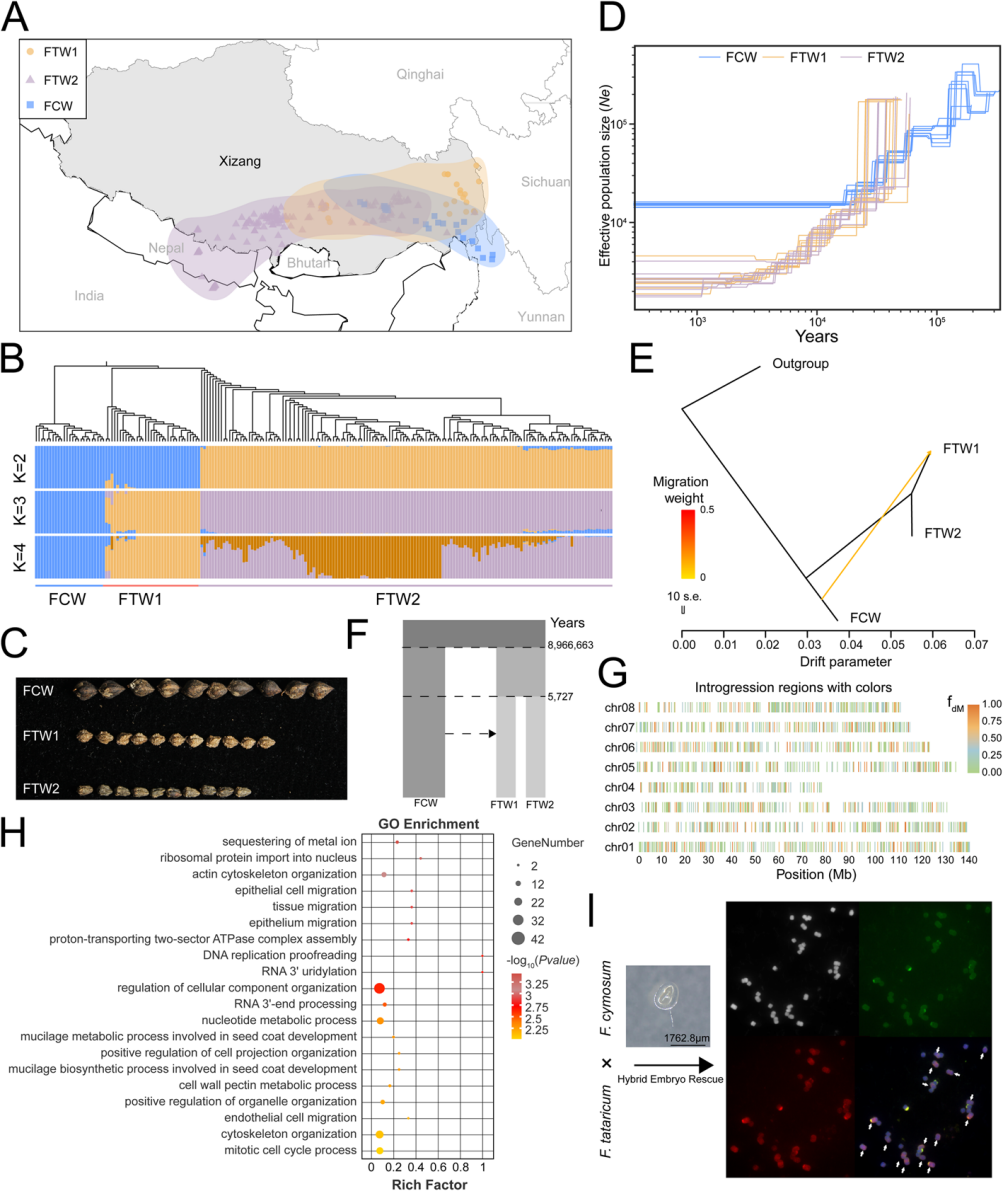
Introgression and interspecific hybridization between wild *F. cymosum* and wild *F. tataricum*. **A** Geographic distribution of collected wild *F. cymosum* and wild *F. tataricum* accessions. FTW1 and FTW2 represent two groups of wild *F. tataricum*. **B** Population structure analysis of wild *F. cymosum* and wild *F. tataricum* accessions, showing subpopulation distributions at *K* = 2 to 4. **C** Seed morphology differences between wild *F. cymosum* and FTW1 and FTW2. **D** Effective population size trends for wild *F. cymosum* and wild *F. tataricum*. **E** Gene flow analysis using TreeMix. The major migration events indicated by yellow arrows. **F** Estimation of interspecific introgression and divergence time between wild *F. cymosum* and wild *F. tataricum*. **G** Introgression analysis from wild *F. cymosum* into wild *F. tataricum*. **H** GO enrichment analysis of genes introgressed from wild *F. cymosum* into wild *F. tataricum*. **I** Embryo rescue and genomic in situ hybridization (GISH) in hybrids of wild *F. cymosum* and wild *F. tataricum* in the FTW1 group

Tibet is both the center of origin and genetic diversity of *F. tataricum* [8]. Typically, *F. cymosum* seeds lack a ventral groove, are relatively large, and exhibit distinct ridges. The seeds of wild *F. cymosum* have a shallow ventral groove, are smaller than typical *F. cymosum* seeds, and lack distinct ridges. These features are similar to *F. tataricum* seeds. Therefore, we hypothesized that gene flow may have occurred between wild *F. cymosum* and Tibetan wild *F. tataricum*, leading to similarities in seed morphology. To test this hypothesis and investigate the phylogenetic relationships and evolutionary history between *F. cymosum* and wild *F. tataricum,* we collected 25 accessions of *F. cymosum* and 181 accessions of wild *F. tataricum* from Tibet (Fig. 5A). These accessions were resequenced to an average depth of 10 × (Additional file 2: Table S22). Alignment to the JQ-MY *F. cymosum* reference genome identified 20 million high-quality SNPs (Additional file 1: Fig. S27; Additional file 2: Table S23). These SNPs were subsequently utilized for population structure and dynamic history analysis, and 206 buckwheat accessions were optimally divided into three groups. The *F. cymosum* accessions formed a distinct subpopulation (FCW), while the wild *F. tataricum* accessions were divided into two groups, designated as FTW1 and FTW2 (Fig. 5B; Additional file 1: Fig. S28).

The seeds of FTW1 are wider, resembling the morphology of wild *F. cymosum*, whereas the seeds of FTW2 are narrower and conical (Fig. 5C; Additional file 1: Fig. S29). FTW1 and FTW2 exhibited significant differences in their geographic distribution. FTW1 is primarily distributed in eastern Tibet, near Sichuan, whereas FTW2 is located in southern Tibet, bordering Nepal. Effective population size is one of the indicators to measure population demographic history [56–58]. The effective population size of *F. cymosum* reached equilibrium approximately 0.1 to 0.01 Mya (Fig. 5D). However, the effective population sizes of FTW1 and FTW2 for wild *F. tataricum* have continued to decline. The most likely cause of this phenomenon is inbreeding depression induced by the self-pollinating reproductive system of *F. tataricum* following its speciation. Genomic differences between *F. cymosum* and *F. tataricum* were identified using d_XY_, where genes were found enriched in the pathways related to flavonoid biosynthesis, which explains the differences in flavonoid content between *F. cymosum* and *F. tataricum* (Additional file 2: Tables S24, S25; Additional file 1: Figs. S30 and S31). Considering the seed similarities between *F. cymosum* and *F. tataricum* in Tibetan, we further analyzed the relationships in the interspecific group. A TreeMix analysis between groups revealed that after the divergence of *F. cymosum* and *F. tataricum*, a recent gene flow from *F. cymosum* to FTW1 happened following the population differentiation of *F. tataricum* (Fig. 5E). Positive D-statistics (*Z*-score = 5.26, *P* = 1.48 × 10^−7^) of the AABA-BABA test confirmed this result (Additional file 2: Table S23). Subsequently, we estimated a demographic model which reflecting the population split and gene flow (Fig. 5F). The model indicated that *F. cymosum* and *F. tataricum* diverged approximately 8.97 Mya, and gene introgression happened in the time after *F. tataricum* further split into FTW1 and FTW2. To investigate the impact of gene flow on wild *F. tataricum*, we utilized positive F_dM_ at the genomic window level to identify introgressed regions from FCW to FTW1. The results showed that introgressed genomic regions are distributed across all chromosomes, with some genes introgressed from *F. cymosum* into FTW1 enriched in pathways related to seed development (Fig. 5G, H; Additional file 2: Table S26). In total, 24 of 44 genes that may be related to seed development were highly expressed in the early development stage of seeds in *F. tataricum* (Additional file 1: Fig. S32; Additional file 2: Table S27). Among them, the homolog of the auxin efflux carrier (jqasm015636) enables seed size regulation in *Arabidopsis* by mediating the dynamic distribution of auxin during transport [59]. The *SS2* gene (jqasm001260), which encodes starch synthase, when inactivated in wheat, leads to significant changes in seed weight, and mutations in this gene result in a shrunken endosperm phenotype in barley [60–63]. The homologous gene, jqasm024771, encoding a B3 domain-containing protein, which directly binds to the *O2* promoter for transactivation, and its mutant results in reduced seed size and decreased accumulation of zein, starch, and lipids in rice. Therefore, we speculated that these introgressed genes from FCW may contribute to larger seeds of germplasms in the FTW1 group.

To investigate whether wild *F. cymosum* is more closely related to FTW1 or FTW2, we conducted hybridization experiments among the three groups. The hybridization success rate between FTW1 and wild *F. cymosum* (0.05%) was significantly higher than that between FTW2 and wild *F. cymosum* (0.03%) (Additional file 2: Table S28). Hybridization experiment results among the three groups further confirmed a closer genetic relationship between wild *F. cymosum* and FTW1. The hybrid progeny between FTW1 and wild *F. cymosum* developed normally through embryo rescue and produced seeds through selfing (Fig. 5I). We conducted genomic in situ hybridization (GISH) to verify that the parents of the hybrid progeny are wild *F. cymosum* and FTW1. The selfed progeny from the hybridization between wild *F. cymosum* and FTW1 exhibited flower/seed sizes similar to the maternal parent (wild *F. cymosum*) and flower colors identical to the paternal parent (FTW1) (Additional file 1: Fig. S33). Therefore, our successful interspecific hybridization further supports a closer phylogenetic relationship between *F. cymosum* and wild *F. tataricum*.

## Discussion

### Limits of traditional flowering plant systematics

Traditional flowering plant systematics primarily infers phylogenetic relationships among species by comparing their external morphological structures, such as plant morphology, leaf shape, flower structure, and fruit type. Species with similar morphologies are often considered to have closer phylogenetic relationships, an assumption that has played a significant role in early taxonomic research. However, it is a common phenomenon that morphology-based classification can lead to ambiguity in phylogenetic relationships. For instance, the genera *Helianthus* and *Aster* in the family *Asteraceae* were classified as closely related groups based on inflorescence structure and fruit type. However, molecular phylogenetic studies have revealed that the genetic relationship between these two genera is not as close as previously inferred from morphological data [64, 65]. In buckwheat, *F*. *cymosum* is recognized as the most probable candidate of the wild ancestor of *F. esculentum* based on the floral and seed morphology of buckwheat species, but actually, *F*. *cymosum* is closer to *F. tataricum* as measured by the comparative genomic and population genetic analyses in our study. Although the *F. cymosum* genomic size is similar to that of *F. esculentum*, this similarity is mainly attributed to analogous transposon bursts. With the rapid development of genome sequences, phylogenomics inferred from the whole plant nuclear and chloroplast genome markers, which can yield robust results for phylogenetic relationships, provides an ideal framework. Recently, several studies indicate that the genetic relationships of many plant species can be reconstructed based on the whole genome sequence data alone. For example, based on morphological differences in pistil structure, the Lamiaceae and Verbenaceae have long been considered distinct families [66]. To clarify ambiguities and correct taxonomic errors, the phylogenetic relationships of Lamiaceae were reconstructed using comparative plastid genomics [67]. The genus *Ajania* has been considered to exhibit a nested phylogenetic relationship with *Phaeostigma* based on capitulum characteristics and limited ribosomal DNA sequence evidence [68, 69]. However, analysis of plastid genome data revealed nucleoplasmic conflict between *Ajania* and *Phaeostigma*, clarifying that no nested phylogenetic relationship exists [70]. It was further suggested that the similarity in capitulum characteristics between *Ajania* and *Phaeostigma* is likely a result of convergent evolution, as adaptation to similar environmental pressures can lead to similar traits in different species. In this study, we used phylogenomic analyses to clarify that despite the phenotypic similarity between *F. esculentum* and *F. cymosum*, *F. cymosum* is more closely related to *F. tataricum* than it is to *F. esculentum*. Although traditional flowering plant systematics based on morphological traits has historically been highly useful, it does not fully reflect true phylogenetic relationships under the framework of modern evolutionary biology and genomics. Therefore, contemporary flowering plant systematics requires an integrative approach combining morphology, molecular biology, and genomics to achieve a more comprehensive understanding of species’ evolutionary histories and phylogenetic relationships.

### Affection of plant morphologic plasticity and hybridization

The transitional forms caused by plant morphologic plasticity and hybridization have made flowering plant systematics challenging. The genus *Plantago* includes numerous morphologically variable species, and morphology alone or limited molecular markers are insufficient to resolve its taxonomic issues [71]. High-quality plastid genome data obtained through resequencing have provided deeper insights into the taxonomy, phylogenetic relationships, biogeography, and evolutionary history of the genus *Plantago*. However, the genus *Plantago* still contains transitional species that have not been correctly classified, which Hassemer attributes to insufficient sampling. Hassemer proposed a solution involving extensive sampling of populations for all species, including their distribution across all environmentally distinct regions [71]. The genus *Stachyurus* includes species with hybridization and reticulate evolution, making classification difficult based solely on morphological analysis [72]. A robust phylogenetic framework for the genus *Stachyurus* was reconstructed using phylogenomics. High-resolution transcriptomic data identified and classified species with complex evolutionary histories, providing insights into their phylogenetic relationships. In summary, the accurate classification of transitional species requires comprehensive sampling of all species and subspecies, ensuring sufficient intraspecific diversity. Additionally, high-resolution nuclear and plastid genome data are essential to elucidate key evolutionary events. It is challenging to determine whether similar traits in two species result from natural hybridization or shared environmental pressures. Population genetic analysis can be used to investigate the causes of convergent phenotypes between two species. Introgression signals resulting from natural hybridization between *Arabidopsis arenosa* and *A. lyrata* were detected through population structure analysis and gene flow analysis [73]. This introgression conferred *A. lyrata* with enhanced adaptability to whole-genome duplication. In the natural hybridization event that formed hexaploid wheat (AABBDD), the *HKT1;5* gene was acquired from *Triticum monococcum* (A^m^A^m^), and this gene plays a key role in mediating Na⁺ tolerance in natural hexaploid wheat [74]. Additionally, genetic introgression of wild *Zea mays* ssp. *parviglumis* enriched with stress-resistance genes has significantly enhanced the environmental adaptability of modern maize varieties [75, 76]. In our study, through extensive sampling and population sequencing, introgression between wild *F. tartaricum* and *F. cymosum* can be detected. Such introgression may contribute to the seed morphological similarity observed between the two species.

### Convergent environment adaptive and selection shaping phenotype

Independent evolution of similar gene variants or functions has been detected in the genomes of unrelated species, often associated with shared environmental selection pressures. For example, to adapt to the arid environment, the plant species from the families of *Cactaceae* and *Euphorbiaceae* have independently evolved succulent stems and reduced leaves as an adaptation to minimize water loss [77]. In high-altitude environments, independent accumulations of *EPAS1* gene variants in Tibetan humans, horses, yaks, and dogs have conferred adaptation to hypoxic conditions [78–82]. In our work, leaf morphological similarity and higher flavonoid content between *F. cymosum* and wild *F. esculentum* might be attributed to their shared high-altitude habitats and the occurrence of convergent evolution. As anticipated, we did not detect cytonuclear discordance between *F. cymosum* and wild *F. esculentum* based on nuclear and chloroplast genomes, indicating the absence of natural hybridization or introgression. Further genetic differentiation regions analysis between wild and cultivated *F. esculentum* revealed that the leaf morphology controlling gene *NAL1* and key flavonoid enzyme gene *F3'H* were detected, which indicate that *F. cymosum* and wild *F. esculentum* may have independently selected different gene combinations to achieve similar plant morphology, enabling adaptation to high-altitude environments [53–55, 83, 84]. Therefore, by analyzing the genetic diversity of extensive sampling of populations across all environmentally distinct regions, we can comprehensively elucidate the genetic similarities between different lineages or species and can distinguish whether trait convergence is caused by introgression or similar environmental selection pressures.

### Geographical distribution and speciation

The relationship between geographical distribution and speciation constitutes an important subject within the field of evolutionary biology. One example is these, three closely related species distributed in the southwestern region of China and the Himalayan Mountains, which maintain their species integrity despite the overlap of their distribution ranges. This phenomenon is intricately linked to a multitude of biological factors. Primarily, from the perspective of reproductive isolation mechanisms, there exists a notable divergence in the flowering periods among these three species, which establishes a temporal barrier to reproduction. Specifically, *F. cymosum* exhibits the latest flowering, while *F. tataricum* has the earliest flowering (Fig. 3A). The disparity in flowering times results in a reproductive isolation barrier among the three buckwheat species. The flowering process in plants is a complex developmental event regulated by a multitude of factors, encompassing environmental influences [85, 86] as well as intrinsic elements [87–89]. Among these, the ABCE model proposed by Coen and Meyerowitz in 1991 stands as a classical framework for elucidating the developmental processes of floral organs. This model elucidates the genetic regulatory mechanisms underlying floral organ development. *AP1*, categorized as a class A gene, primarily governs the development of sepals and petals [90]. It also plays a pivotal role in the determination of floral meristems, facilitating the transition from inflorescence meristems to floral meristems [91, 92]. The C-terminal region of AP1 harbors a conserved transcriptional activation domain, enabling interactions with other regulatory factors, such as SOC1, to modulate the expression of downstream genes [34]. Among the three cultivated species of buckwheat, structural variations in the *AP1* gene have been observed (Fig. 3E–K). The *AP1* gene structure is consistent between *F. tataricum* and *F. esculentum*. However, *F. cymosum* exhibits a deletion in the C-terminal region of the *AP1* gene, resulting in the loss of its functional domain. More critically, the truncated AP1 protein, lacking the complete domain, is unable to interact with the SOC1 protein. This impaired interaction likely influences the flowering time, contributing significantly to the delayed flowering observed in *F. cymosum*. Secondly, the differentiation in floral organ structure among these species constitutes a significant morphological isolation mechanism, particularly through the formation of dimorphic flowers, which is closely associated with the regulatory expression of the *SELF3* gene, which governs self-incompatibility in buckwheats [11, 35]. In *F. cymosum* and *F. esculentum*, the *SELF3* gene retains its complete structural integrity. However, in *F. tataricum*, an insertion of a transposable element and subsequent mutation in the *SELF3* gene on chromosome 7 has rendered it a pseudogene, thereby abolishing its self-incompatibility function. Therefore, both *AP1* and *SELF3* genes may probably regulate the development of floral organs and flowering time and influence the efficiency of both self-pollination and cross-pollination, thereby maintaining the genetic distinctiveness of these three species.

### Closet relationship enhancing the interspecific hybridization

In the discourse on interspecific relationships among plants, hybridization emerges as a pivotal research trajectory, particularly the phenomena of natural hybridization and homoploid hybridization, along with their implications for species evolution and breeding endeavors [93]. Natural hybridization refers to the interbreeding between distinct species or subspecies under natural conditions, a phenomenon relatively common in the plant kingdom, especially among closely related species. This process facilitates gene flow, augments genetic diversity, and furnishes novel genetic materials that may enhance species adaptability to environmental changes [94]. Natural hybrids of *Ligularia nelumbifolia* and *Cremanthodium stenoglossum* were discovered in overlapping habitats at a 4600-m plateau environment, northwestern Sichuan [95]. Morphological and genomic evidence both support that the hybrids are more closely related to *L. nelumbifolia*, and adaptive changes to the alpine environment (reduced leaf size) were observed. Natural hybrids between *Rhododendron delavayi* and *R. irroratum* were discovered in the Baili Scenic Reserve, western Guizhou [96]. These hybrids possess higher horticultural value than azaleas, characterized by larger leaves, improved flower shapes, stronger fragrance, more diverse flower colors, and extended flowering periods. In the karst regions of southwestern China, *Camellia ptilosperma* was identified as a natural hybrid of *C. micrantha* and *C. flavida* [97]. *C. ptilosperma* demonstrates enhanced drought tolerance compared to its parental species, allowing it to thrive on karst slopes with limited shallow soil water. Consequently, its ecological niche extends beyond the habitat ranges of its progenitors. Hybrid offspring often exhibit superior traits. However, artificially simulating these natural hybrids is challenging due to the difficulty in identifying compatible materials and the lengthy breeding cycles [96, 98, 99]. Consequently, horticultural breeders prefer to utilize natural hybrids for improving cultivars through backcrossing [96]. Distant interspecific hybridization, such as cultivated species crosses with their wild relative species, has the big problem of prezygotic or postzygotic barriers due to substantial genetic divergence and complete reproductive isolation [100]. *F. cymosum* diverged from *F. tataricum* approximately 7.49 million years ago (Mya); thus, direct hybridization between these two species fails to produce mature embryos due to strong reproductive barriers stemming from high genetic divergence. Through exhaustive genomic analysis of *F. cymosum*-related wild resources FCW and *F. tataricum*-related wild resources FTW, we found that their genomic divergences are reduced. Therefore, we crossed FCW with the FTW lineage and broke prezygotic barriers and obtained hybrid embryos, further with the help of embryo rescue and chromosome doubling, we were able to generate sterile progeny (Fig. 5). Many crops still cannot cross with their wild relative species due to lack of available compatible lineages or species, thus it is necessary to evaluate the phylogenetic relationships between cultivated species with their wild relatives by phylogenomics. Thus, as well as clarifying a few aspects of the evolution of buckwheat, our study provides a valuable example that phylogenomics can be efficiently utilized for phylogenetic relationship analysis between crops and their wild relatives, and dissect the evolution in high-altitude lineages while maintaining their species boundaries.

## Conclusions

We demonstrated that *F. cymosum* is more closely related to *F. tataricum* than to *F. esculentum*. Therefore, we suggest that contemporary flowering plant systematics requires an integrative approach that combines morphology, molecular biology, and genomics to achieve a more comprehensive understanding of the evolutionary history and phylogenetic relationships of species. Through the comparison of morphology and genome among these species, flowering time and style morphology were found to contribute significantly to plant speciation without distinct geographical isolation. Introgression and interspecific hybridization between *F. cymosum* and wild *F. tataricum* have led to changes in seed morphology, providing a valuable example for the utilization of crops and their relatives. Living in interspecific groups in the high-altitude regions, the wild *F. esculentum* and *F. cymosum* tend to the small leaf sizes and high flavonoid content, which are associated with adaptation to alpine environments. Overall, this study provides comprehensive insight into the evolutionary relationships between buckwheat and its wild relatives, which also lays the foundation for future breeding efforts.

## Methods

### Plant material

Plant materials used for transcriptome sequencing were from FT cv. Pinku, with samples collected at three stages: early flower bud (FTS1), mid-flower bud (FTS2), and late flower bud (FTS3), cultivated in a greenhouse (National Crop Genebank of China, Haidian District, Beijing). The golden buckwheat materials were sourced from Tibet and Yunnan regions. The Tartary buckwheat materials were collected from the Tibet region. The wild common buckwheat materials were sourced from the Yunnan, Sichuan, and Tibet regions. The cultivated common buckwheat materials were sourced from central and northeastern China, as reported in our previous publication [11].

### Paraffin embedding and staining

To prepare paraffin sections for histological analysis, the samples were sequentially treated as follows: first, they were immersed in Environmental Friendly Dewaxing Transparent Liquid I for 20 min, followed by Environmental Friendly Dewaxing Transparent Liquid II for an additional 20 min. The sections were then dehydrated through immersion in anhydrous ethanol I and II for 5 min each and subsequently transferred to 75% ethyl alcohol for 5 min. After these steps, the sections were rinsed thoroughly with tap water to remove residual alcohol. Next, the plant tissue slices were stained with Toluidine Blue for 2 min to enhance tissue visualization, followed by another rinse with tap water to remove excess stain. Tissue coloration was inspected microscopically to determine whether differentiation or non-differentiation was appropriate based on the staining results. After microscopic evaluation, the sections were washed with tap water once more and dried in an oven. For final processing, the tissue sections were rendered transparent using xylene for 5 min. Finally, the samples were sealed with neutral gum for preservation. The prepared sections were then subjected to microscopic inspection, and image acquisition and analysis were performed to document and analyze the tissue structures.

### DNA extraction and RNA extraction

Young Tartary buckwheat leaves during the nutritional growth period were used to extract genomic DNA with reference to the CTAB method [101]. Total RNA was extracted using the RNApre Pure Factory Plus Kit (DP441, Beijing, China) and HiScript III RT SuperMix for qPCR (R323-01, Vazyme, Nanjing, China) was used for reverse transcription.

### Genome assembly and annotation

A minimum of 1 μg of genomic DNA per accession was utilized to construct sequencing libraries, following the manufacturer’s protocol provided by Illumina. Sequencing was performed on an Illumina NovaSeq 6000 platform at Berry Genomics, generating paired-end libraries with an average insert size of approximately 350 bp. Quality control of the raw fastq data was conducted using Trimmomatic v0.33, which removed adapters based on the manufacturer’s sequences. Subsequently, custom Perl scripts were employed to process the raw data. During this step, clean reads were generated by filtering out those containing adapters, poly-N sequences, or low-quality bases. The genome of the easily dehulled type (EDT) was assembled using PacBio HiFi sequencing data processed with the hifiasm assembler [102]. Hi-C data was aligned to the contigs utilizing the Juicer v1.6.2 pipeline [103]. Initial scaffolds were generated with 3D-DNA v180922 using default settings [104]. The resulting assembly was reviewed and refined through manual curation with Juicebox Assembly Tools v1.9.8 [105]. A subsequent round of scaffolding with 3D-DNA v180922 was carried out to produce the final pseudo-chromosomes. The completeness of the assembled genome was evaluated using the Benchmarking Universal Single-Copy Orthologs (BUSCO) framework [106], referencing the conserved genes from Embryophyta_odb10. We employed a comprehensive approach utilizing the BRAKER pipeline [107], which integrates homology-based, transcriptome-based, and de novo methods to predict high-quality protein-coding genes. Homologies were obtained from the accessible genome annotation of three buckwheat species (*F. cymosum* and *F. esculentum*, and *F. tataricum*). Additionally, we conducted an independent transcriptome-based prediction using HISAT2 and PASA [108], analyzing Illumina sequencing data obtained from various tissues, including root, stem, leaves, and flowers. Finally, we utilized EVidenceModeler v2.1.0 (https://github.com/EVidenceModeler/EVidenceModeler) to integrate the gene models generated from all prediction methods. To annotate non-coding genes in the genome, we used Infernal (http://eddylab.org/infernal/) to search for homologs in the Rfam database (https://rfam.xfam.org/).

### Transposon analysis

Repetitive sequences in the genome were annotated using a combination of homology-based methods and de novo approaches. Comprehensive identification and classification of transposable elements (TEs) were performed using the Extensive de novo TE Annotator (EDTA) pipeline, which integrates multiple tools for detecting and categorizing TEs. Default parameters were applied to generate an initial annotation, encompassing long terminal repeat retrotransposons (LTR-RTs), DNA transposons, and other repetitive elements. Full-length LTR-RTs were identified through LTR_Finder and LTR harvest and further refined using the LTR_retriever module within EDTA to enhance accuracy and eliminate false positives. LTR_retriever also estimated LTR-RT insertion times based on the genetic distance formula T = K/2rT = KK/2r, where KK represents sequence divergence. To ensure data reliability, low-confidence elements were excluded, resulting in a curated dataset suitable for downstream genomic analyses.

### Comparative genome analysis and evolution

First, we downloaded the published genomes assemblies of Tartary buckwheat cv. Pinku (http://www.mbkbase.org/Pinku1) [22], golden buckwheat cv. LJS (10.6084/m9.figshare.19711891.v2) [23], and common buckwheat cv. Pintian (https://ngdc.cncb.ac.cn/gwh/search/advanced/result?search_category=&search_term=&source=0&query_box=GWHBJBK00000000) [24] for further analysis. We conducted a macro-level collinearity analysis using the JCVI (Python version of MCScan) software to detect the anchor genes and chromosome arrangement [109]. The WGD genes were estimated to obtain a Ks distribution model using WGD software. To define the systematic evolutionary relationships of cultivated buckwheat, more than three thousand single-copy genes were identified for the following analysis. A series of single-gene tree and coalescent-based species trees were estimated using the ASTRAL pipeline [110] and visualized in DensiTree v3.0 (https://github.com/rbouckaert/DensiTree). Using single-copy genes from *F. tataricum*, *F. esculentum*, and *F. cymosum* as foreground branches, positively selected genes were identified employing the branch-site model of the CODEML program within the PAML package. For the phylogenetic tree containing multiple species, including *Beta vulgaris*, *F. tataricum*, *F. esculentum*, *F. esculentum* ssp. *ancestrale*, *F. cymosum*, *Arabidopsis thaliana*, *Fallopia multiflora*, *Glycine max*, *Rheum nobile*, *R. tanguticum*, and *R. palmatum*, their protein sequences were used to cluster and detect single-copy genes by Orthofinder v2.5.5 [111], generating three hundred and twenty genes to build a species tree using RAxML with “PROTGAMMALGX” model. The aligned sequence and species tree were subsequently estimated divergence time using the MCMCTREE in the package of PAML (http://abacus.gene.ucl.ac.uk/software/paml.html). CAFÉ5 was utilized to identify gene family expansion and contraction across the phylogeny [112].

### Transcriptome analysis

For the analysis of gene expressions in transcriptomics, raw sequencing reads were initially filtered using the fastp v. 0.20 to remove adaptor and low-quality base. Subsequently, clean reads were aligned to the reference genome *F. tataricum*. Pinku (HERA) using HISAT2 (version 2.1.0) with default parameter settings [113]. The resulting alignment files were processed with FeatureCounts (version 2.0.3) to quantify gene expression, producing a count matrix [114]. This matrix was then transformed into a gene FPKM matrix using Python scripts.

### Genome resequencing and SNP calling

Initially, total genomic DNA was extracted from a single plant per accession using a modified CTAB method. Libraries with an insert size of 500 bp were constructed and sequenced on the Illumina HiSeq X Ten platform (Illumina, San Diego, CA), generating 150-bp paired-end (PE) reads. Sequencing was conducted by Berray Gene Technology (Beijing, China). The raw paired-end reads were filtered using fastp v. 0.20 [115]. After filtering, the clean reads of each accession were then mapped onto the reference genome using the BWA-MEM program in BWA v0.7.17 with default parameters [116]. Then, the mapped reads were sorted using SAMtools (v1.3.1), and duplicated reads were removed using Picard (v1.13) (http://broadinstitute.github.io/picard/). The Genome Analysis Toolkit (GATK4, https://www.sentieon.com/) was applied for variant calling. The HaplotypeCaller model was used for SNPs and Indels calling, and the GVCFs of each accession were merged using the CombineGVCFs function. The SNPs were filtered with VariantFiltration (QD < 2, FS > 10.0, DP < 4, QUAL < 30 and SOR > 3). ANNOVAR was employed to annotate all the qualified variants on genomic genes using the default parameter [117].

### Chloroplast genome assembly and phylogenetic construction

Low-quality reads were filtered using the trimmomatic software to ensure high-quality data for assembly. GetOrganelle v1.7.7.1 was used to assemble the chloroplast genome based on the genome resequencing reads with the parameter “-R 15 -k 21,65,105” [118]. A circular chloroplast genome circular was obtained and used for follow analysis. For the annotation of chloroplast genomes, the published assembly file (NC_037705.1) of *F. cymosum* was used as reference, and the PGA software were used to annotated the newly sequenced chloroplast genomes of each sample [119]. The single-copy genes of the chloroplast genome were extracted using the script, followed by multiple sequence alignment and trimming with the default parameter. Subsequently, an evolutionary tree was constructed using RAxML software, with 1000 bootstrap replicates for validation [120].

### Population structure analysis

For the phylogenetic analysis using SNP datasets, a neighbor-joining tree was constructed for all samples using TreeBest 1.9.2 with 100 bootstrap replicates [121]. Principal component analysis (PCA) was performed using PLINK1.9 (https://www.cog-genomics.org/plink/). Population structure was inferred from large SNP genotype datasets using ADMIXTURE (version v1.3.0) [122]. The analysis was conducted with *K* values ranging from 2 to 8. The LD linkage disequilibrium was assessed and plotted using the PopLDdecay v3.41 (https://github.com/BGI-shenzhen/PopLDdecay). To assess genetic differentiation and divergence between populations, we employed the VCFtools and Pixy to calculate the fixation index (F_ST_) and the nucleotide diversity [123].

### Population demographic history

For the gene flow analysis, the SNP dataset was filtered using “-maf 0.05 -mac 2” in VCFtools and transformed into the input of TreeMix (v.1.13) with the running parameter “-m -se –bootstrap” for 3 replicates. ABBA-BABA analysis was performed by the Dtrios function in Dsuite v0.5 [124]. SMC + + was used to estimate the population size. We randomly selected ten different samples of each group per time and ran 20 repeats that covered all samples [125]. The mutation rate was set as 7 × 10^−9^ per synonymous site for each generation. A population demographic model was built using the built-in algorithms in FastSimicol2 [126] to estimate the parameters of the model. To refine the introgressed genomic regions, f_dM_ statistics of genomic window were evaluated using Python scripts (https://github.com/simonhmartin/genomics_general) with 50-kb sliding windows and a 50-kb step. The flow chart of data processing in this study is presented in Fig. S34.

### Subcellular localization

The coding sequences (CDS) of the target genes were cloned into the pCAMBIA1300 vector (Additional file 2: Table S29), which was subsequently introduced into *Agrobacterium tumefaciens* GV3101. Subcellular localization was analyzed using a Zeiss LSM900 laser confocal microscope, with mCherry detected at 561 nm (excitation) and 590–640 nm (emission) and GFP observed at 488 nm (excitation) and 500–530 nm (emission).

### Transgenic *Arabidopsis thaliana* and phenotypic assays

Total RNA was extracted using the RNApre Pure Plant Plus kit (Tian Gen, Beijing, China). First-strand cDNA was synthesized using HiScript III RT SuperMix for qPCR (Vazyme, Nanjing, China). The coding sequence was cloned into pCAMBIA-1302. The *Arabidopsis* overexpression lines were generated through *Agrobacterium tumefaciens* GV3107-mediated transformation [127]. Positive plants were identified by PCR and propagated to the T3 generation for observation of bolting and flowering time.

### Luciferase complementation assay

The pCAMBIA1300-nLuc and pCAMBIA1300-cLuc vector systems were used to detect protein interactions (Additional file 2: Table S29). FtAP1 and FcAP1 were constructed in pCAMBIA1300-nLuc. While FcSOC1 and FtSOC1, as well as AtSOC1, were constructed in pCAMBIA1300-cLuc. The recombined vectors were transferred into *Agrobacterium* GV3101. Positive clones were cultured until the concentration reached 1 OD. After resuspension in MES solution (2 mM MES, 10 mM MgCl2, 1% APS), the solution was injected into 4-week-old *Nicotiana benthamiana* leaves. After dark incubation for 1 day, the injection sites were treated with D-Luciferin potassium salt (Coolaber, China), and imaging was performed using the Tanon 5200 luminescence imaging system. LUC enzyme activity was detected using the Dual Luciferase Reporter Assay Kit (Vazyme, China).

### Yeast two hybrid

The system was constructed using the pGBDT7 and pGADT7 vectors (Additional file 2: Table S29). *FtAP1* and *FcAP1* were constructed into the pGADT7 vector, while FtSOC1, FcSOC1, and AtSOC1 were constructed into the pGBDT7 vector. The Y2H yeast (ZOMANBIO, Beijing, China) was used for the yeast transformation experiment. Positive yeast clones were subsequently plated on minimal synthetic defined (SD) media lacking leucine and tryptophan (SD-L/T) and on SD media lacking leucine, tryptophan, histidine, and adenine (SD-L/T/H/Ade) to verify the interaction.

### MBP (Maltose Binding Protein)-pull down

The *FtAP1* and *FcAP1* genes were cloned into the pMAL-c5X vector, while the *FtSOC1* gene was cloned into the pET-28a (+) vector using the primers (Additional file 2: Table S29). BL21 (DE3) chemically competent cells (WEIDI, China) were used to transform the recombinant plasmids. Positive colonies were cultured at 37 °C until the optical density (OD) at 600 nm reached 0.8. Protein expression was induced overnight at 20 °C and 120 rpm by adding 1 mM isopropyl *β*-D-thiogalactoside (IPTG). The cells were lysed in a buffer containing 50 mM Tris, 150 mM NaCl, 1 mM DTT (pH 7.5). The supernatant was collected and incubated with the required protein combinations for 1 h. Pre-equilibrated MBPSep Dextrin Agarose Resin (Yeasen, China) was added to the supernatant and allowed to bind for 1 h. The resin was then washed five times with the same buffer. The proteins were eluted using an elution buffer (containing 50 mM Tris, 150 mM NaCl, 20 mM maltose, and 1 mM DTT, pH 7.5). Protein samples were mixed with 5X SDS-PAGE Sample Loading Buffer (ABclonal, China) and denatured at 100 °C for 10 min. Proteins were separated by sodium dodecyl sulfate–polyacrylamide gel electrophoresis (SDS-PAGE) and detected by Western blot analysis [128]. Proteins were transferred from SDS-PAGE gels to Immun-Blot PVDF membranes (Bio-Rad, China). The membranes were incubated for 1 h with primary antibodies, including Anti-MBP Tag mouse monoclonal antibody (Sangon Biotech, China) and His-tag mouse monoclonal antibody (Beyotime, China), respectively. After five washes with TBST buffer, the membranes were incubated with Goat anti-Mouse IgG antibodies (Thermo Fisher Scientific, USA) for 1 h. Following another five washes with TBST, the proteins were visualized using Immobilon Western HRP Substrate (Merck, Germany) and a Tanon 5200 luminescence imaging system used for image acquisition.

### Interspecific hybridization experiment and cytological analysis

We first attempted interspecific crosses between *F. cymosum* and *F. esculentum*, as well as between *F. cymosum* and *F. tataricum*, and observed and counted the number of embryos. Subsequently, the embryo rescue was conducted with the steps: sterilized in 70% alcohol for 2 min, then 10% sterile calcium hypochlorite for 20 min, and then rinsed 5–6 times in sterile water. Embryos were cultured on B5 medium of the inducer (2.0 mg/L 2,4-D, 0.5 mg/L NAA, 0.5 mg/L IAA, 0.2 mg/L Kinetin, 2.0 mg/L casein hydrolysate, 25 g/L sucrose, and 0.8% agar) under a stereomicroscope (DMSZ7). After dark culture at 24℃ for 2 weeks, waiting for the formation of callus and proembryonic cell complexes (PECCs). PECCs were split for regeneration in MS medium (containing 2.0 mg/L 6-BA, 1.0 mg/L Kinetin, 30 g/L sucrose, and 3 g/L gelrite) at 24 °C, 16 h/8 h (light/dark) for 3 weeks. The developed young shoots in embryos were transferred to MS medium for further cultivation into seedling. To cytologically examine the induced allotetraploid from inter-hybridization using the genomic in situ hybridization (GISH) analysis, genomic DNA from *F. cymosum* and *F. tataricum* was labeled utilizing the Atto550 NT labeling kit (Jena Bioscience, Jena, Germany). The GISH protocol was carried out following previously established methods. No blocking DNA was incorporated in the procedure. Chromosome preparations were counterstained with DAPI (4′,6-diamidino-2-phenylindole) using Vectashield (Vector Laboratories, Burlingame, USA). The hybridization signals were visualized and documented with a BX-63 epifluorescence microscope, which was outfitted with a Photometric SenSys Olympus DP70 CCD camera (Olympus, Tokyo, Japan).

### Statistical analysis

Phenotypic data for flowers and seeds are presented as mean ± SD. Correlation analyses between genome size and gene number, as well as Student’s *t*-tests for significant differences between groups, were conducted using GraphPad Prism 8 (GraphPad Software, USA). Descriptive statistics for the phenotypic data were analyzed using Microsoft Excel 2024 (USA).

## Supplementary Information

Additional file 1: Supplemental Figures. Contains compiled supplementary figures and legends referenced in the main text

Additional file 2: Supplemental Table. Contains summary data

## Data Availability

The previous genome assembly of FT cv. Pinku (http://www.mbkbase.org/Pinku1) [22], FT cv. Qianku (10.6084/m9.figshare.21617562.v4) [129], FT cv. MQ (http://buckwheat-gpdb.cn/#/download) [130], FC cv. LJS-YGC (10.6084/m9.figshare.19711891.v2) [23], FC cv. JQ-YN (https://figshare.com/articles/dataset/Fagopyrum_dibotrys/22240414/1) [131], FE cv. Pintian (https://ngdc.cncb.ac.cn/gwh/search/advanced/result?search_category=&search_term=&source=0&query_box=GWHBJBK00000000) [24], FE cv. PL4 (https://ddbj.nig.ac.jp/search/entry/bioproject/PRJDB15031) [35], and FE cv. XN9976 (10.6084/m9.figshare.21617562.v4) [129] were downloaded online and used for further analysis. The previous resequencing data of wild and cultivar type of FE and Outgroup *Fagopyrum* species were accessible at (https://ddbj.nig.ac.jp/search/entry/bioproject/PRJDB15031) [35], (https://ngdc.cncb.ac.cn/gsa/browse/CRA012512) [11], and (https://ngdc.cncb.ac.cn/gsa/browse/insdc/SRA1062765) [132]. In addition, all data generated in this study are publicly available. The genome sequencing data used for genome assembly, annotation, and population analysis are available from the Genome Sequence Archive (https://ngdc.cncb.ac.cn/gsa/browse/CRA028530) of National genomics Data Center under BioProject ID PRJCA035181 [133]. The chromosome-level assembly and annotation file of wild *F. esculentum* (*F. esculentum* ssp. *ancestrale* DDX) are also under the Genome Warehouse (GWH) with accession GWHGPYG00000000 which available at https://ngdc.cncb.ac.cn/gwh/Assembly/101666/show [134]. The chromosome-level assembly and annotation file of wild *F. esculentum* DDX, completed chloroplast genome of FE and FC, and RNA-seq expression pattern file of flower time are also available at Figshare (10.6084/m9.figshare.29673392.v3) as described in ref. [135]. The source code and scripts are publicly accessible at GitHub (https://github.com/youlongjizi/Buckwheat_Phylo_code) [136] and Zenodo (10.5281/zenodo.17055081) [137] under the MIT License.
