# Supplementary material for: Phylogenomics provides comprehensive insights into the evolutionary relationships among cultivated buckwheat species
Source: Genome Biol. 2025 Oct 1;26:327. doi: 10.1186/s13059-025-03793-2 (PMC12487180; doi:10.1186/s13059-025-03793-2)
Supplement: Supplementary file 1 — Additional file 1: Supplemental Figures. Contains compiled supplementary figures and legends referenced in the main text [file 13059_2025_3793_MOESM1_ESM.pdf]

Supplementary Materials for

Phylogenomics provides comprehensive insights into the evolutionary relationships among cultivated buckwheat species

Yaliang Shi[1†], Bo Li[2†], Yuanfen Gao[1†], Xiaohan Wang[1†], Yang Liu[1†], Xiang Lu[1], Hao Lin[1], Wei Li[1], Dili Lai[1], Ming Hao[3], Jia Gao[1], Kaixuan Zhang[1], Dengcai Liu[3], Sun-Hee Woo[4], Muriel Quinet[5], Alisdair R Fernie[6], Xu Liu[1*], Yuqi He[1*], Meiliang Zhou[1*]

[1.] National Key Facility for Crop Gene Resources and Genetic Improvement/Key Laboratory of Grain Crop Genetic Resources Evaluation and Utilization, Ministry of Agriculture and Rural Affairs. P. R. China, Institute of Crop Sciences, Chinese Academy of Agricultural Sciences, Beijing 100081, China

[2.] Center for Integrative Conservation, Xishuangbanna Tropical Botanical Garden, Chinese Academy of Sciences, Mengla 666303, China

[3.] Triticeae Research Institute, Sichuan Agricultural University, Chengdu 610000, China

[4.] Department of Crop Science, Chungbuk National University, Cheongju 28644, Korea

[5.] Groupe de Recherche en Physiologie Végétale (GRPV), Earth and Life Institute-Agronomy (ELI-A), Université catholique de Louvain, Croix du Sud 45, boîte L7.07.13, B-1348 Louvain-la-Neuve, Belgium

[6.] Department of Molecular Physiology, Max-Planck-Institute of Molecular Plant Physiology, Potsdam 14476, Germany

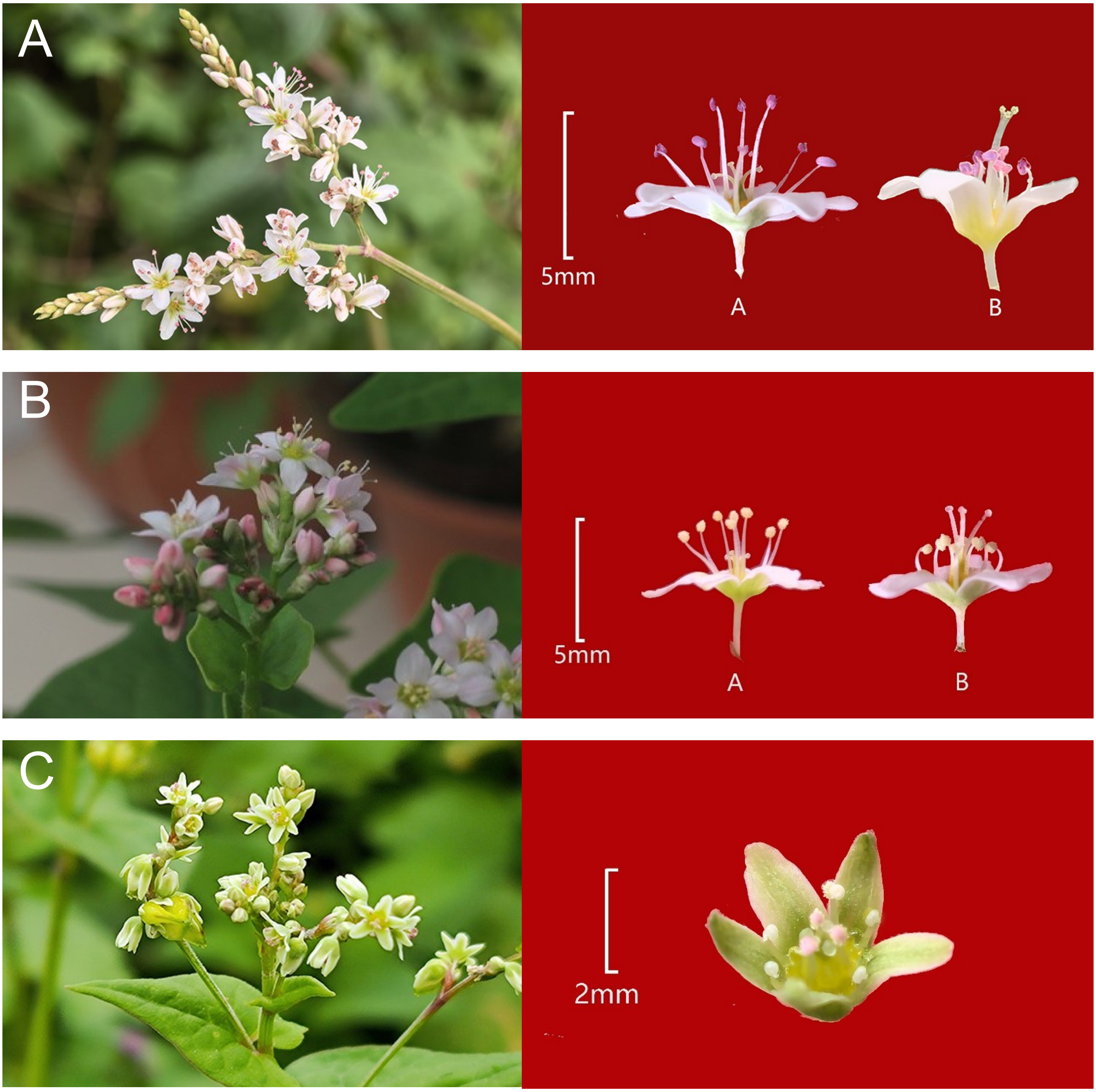

Fig. S1. Morphological polymorphism of the three buckwheat flower styles. Both *Fagopyrum cymosum* **(A)** and *F. esculentum* **(B)** have types with heteromorphic styles [A, S-morph (short styles and long-level anthers); B, L-morph (long styles and short-level anthers)]. The stamen and pistil of *F. tataricum* **(C)** are of equal length, and it is completely self-pollinating.

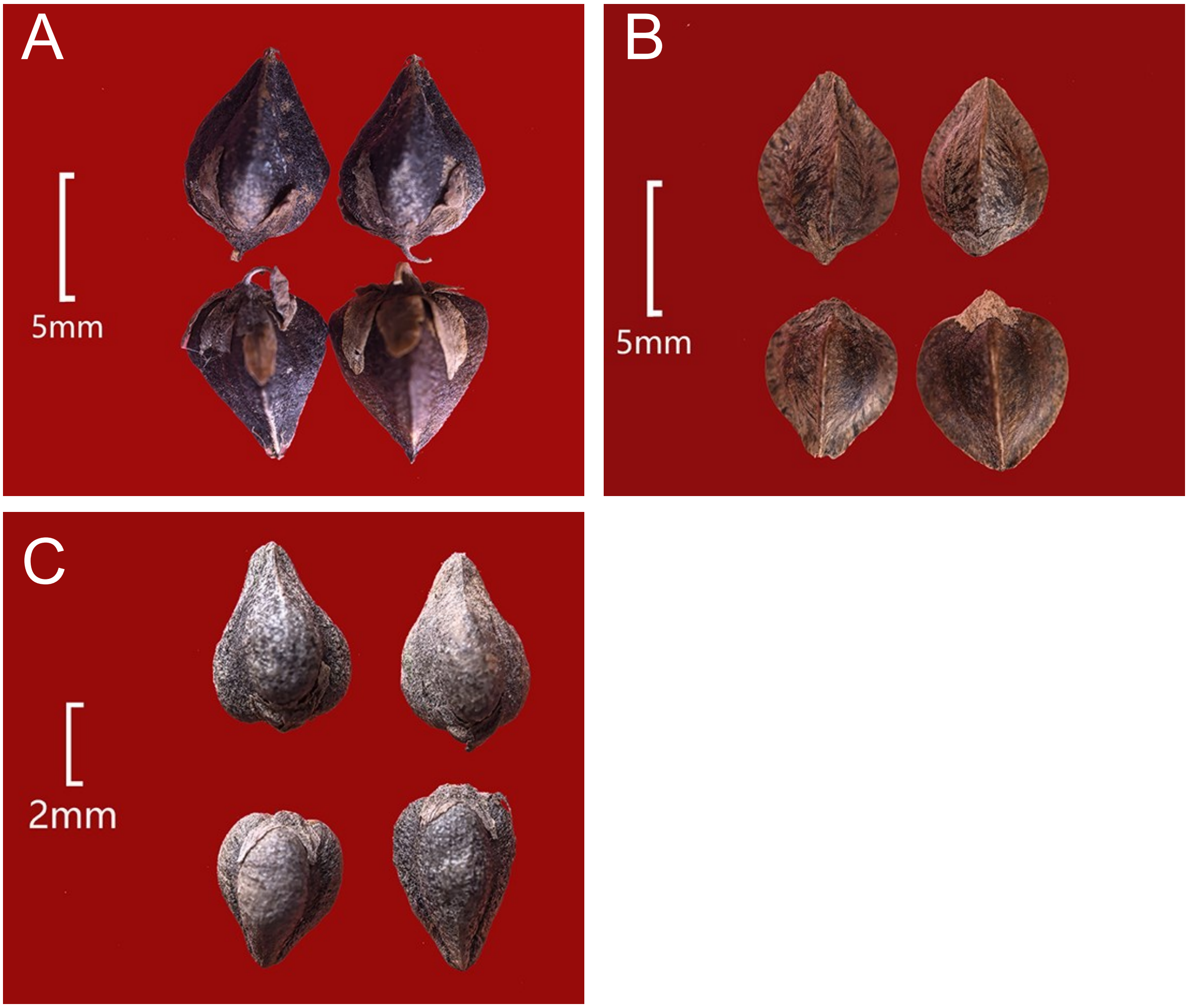

Fig. S2. The seeds of three buckwheat species. The seeds of **(A)** *F. cymosum* and **(B)** *F. esculentum* are relatively large and exhibit distinct edges, whereas the seeds of **(C)** *F. tataricum* are more rounded and have a smoother texture.

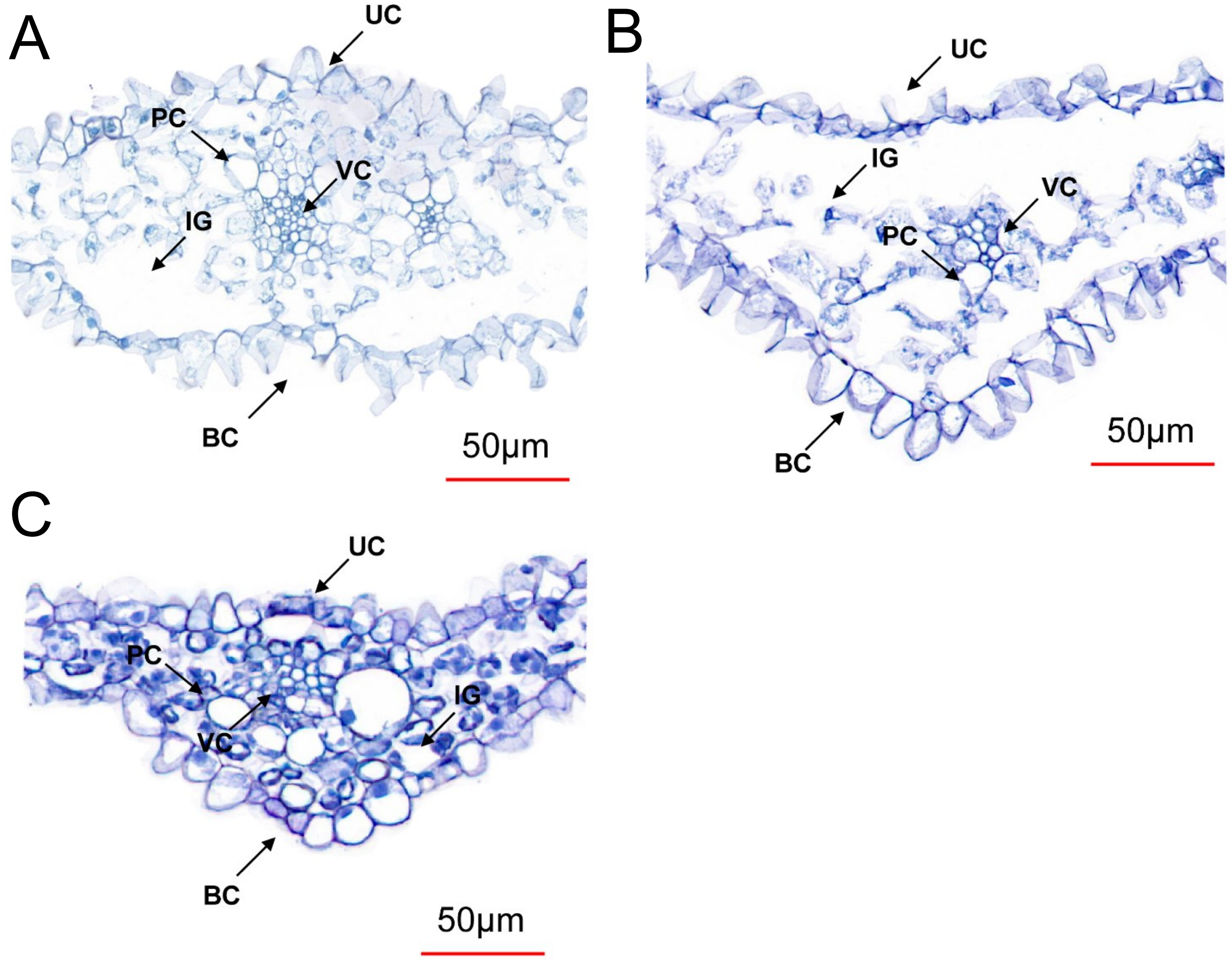

Fig. S3. Longitudinal section microscopic images of floral organs under the flowering period of (A) *F. cymosum*, (B) *F. esculentum*, and (C) *F. tataricum*. BC, basal epidermal cells; PC, parenchyma cells; UC, upper epidermal cells; VC, vascular cells; IG, intercellular gaps.

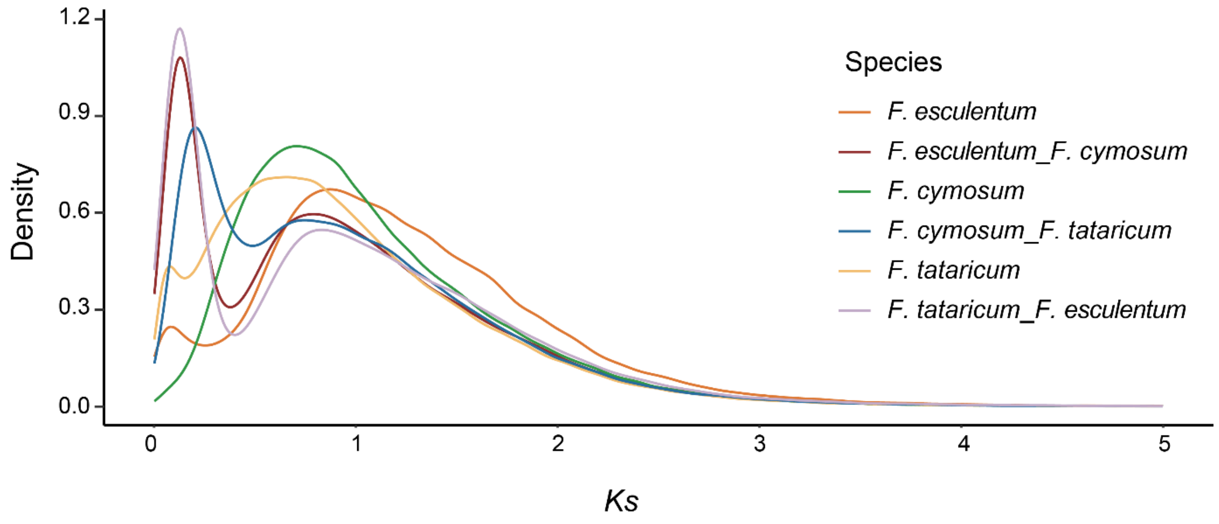

Fig. S4. **Ks distribution density curve between *F. cymosum*, *F. esculentum*, and *F. tataricum*.**

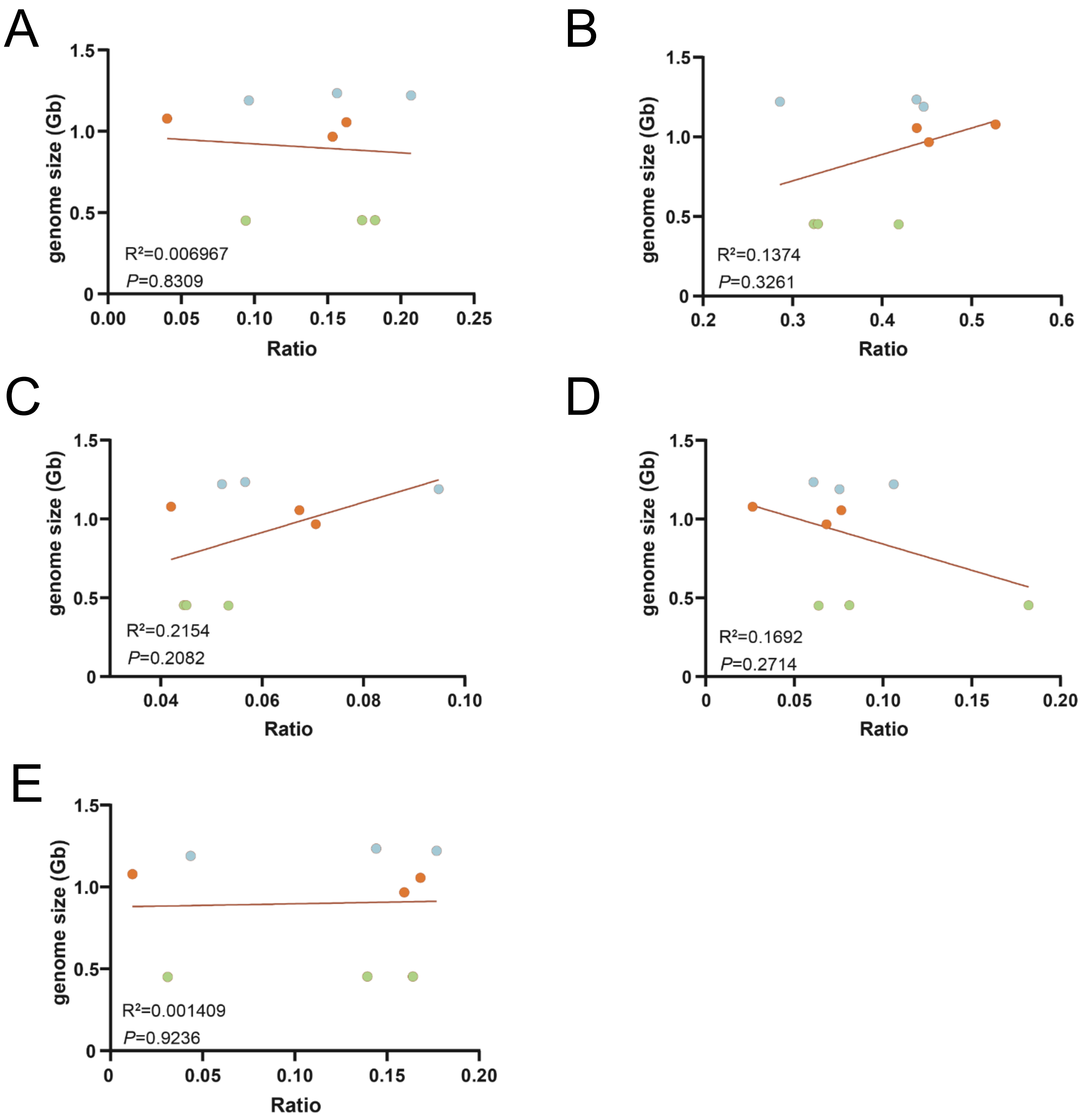

Fig. S5. Analysis of the correlation between five types of duplication genes and genome size. **A** Whole-genome duplication. **B** Dispersed duplication. **C** Proximal duplication. **D** Tandem duplication. **E** Transposed duplication.

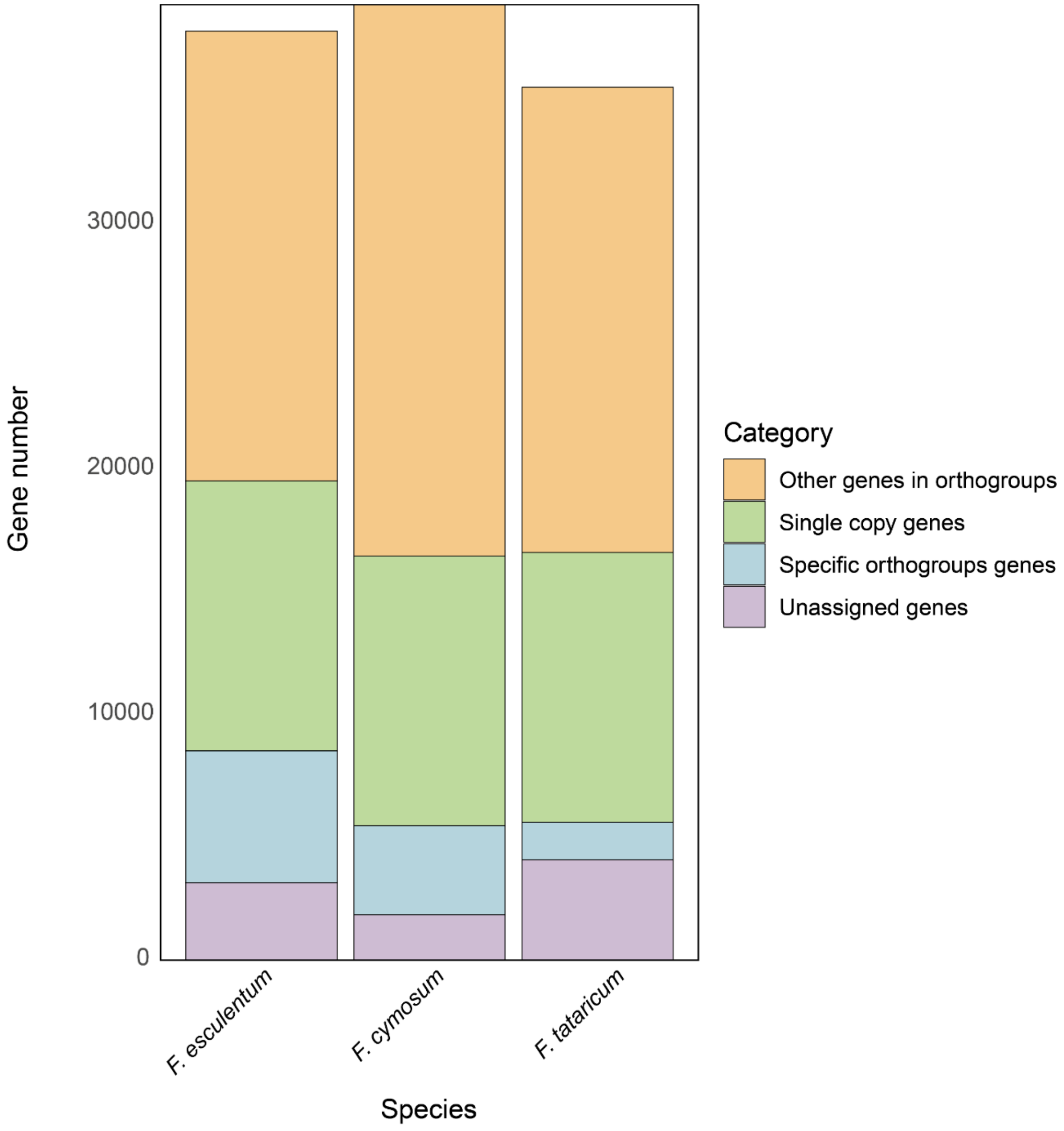

Fig. S6. Classification statistics of *F. cymosum*, *F. esculentum*, and *F. tataricum.*

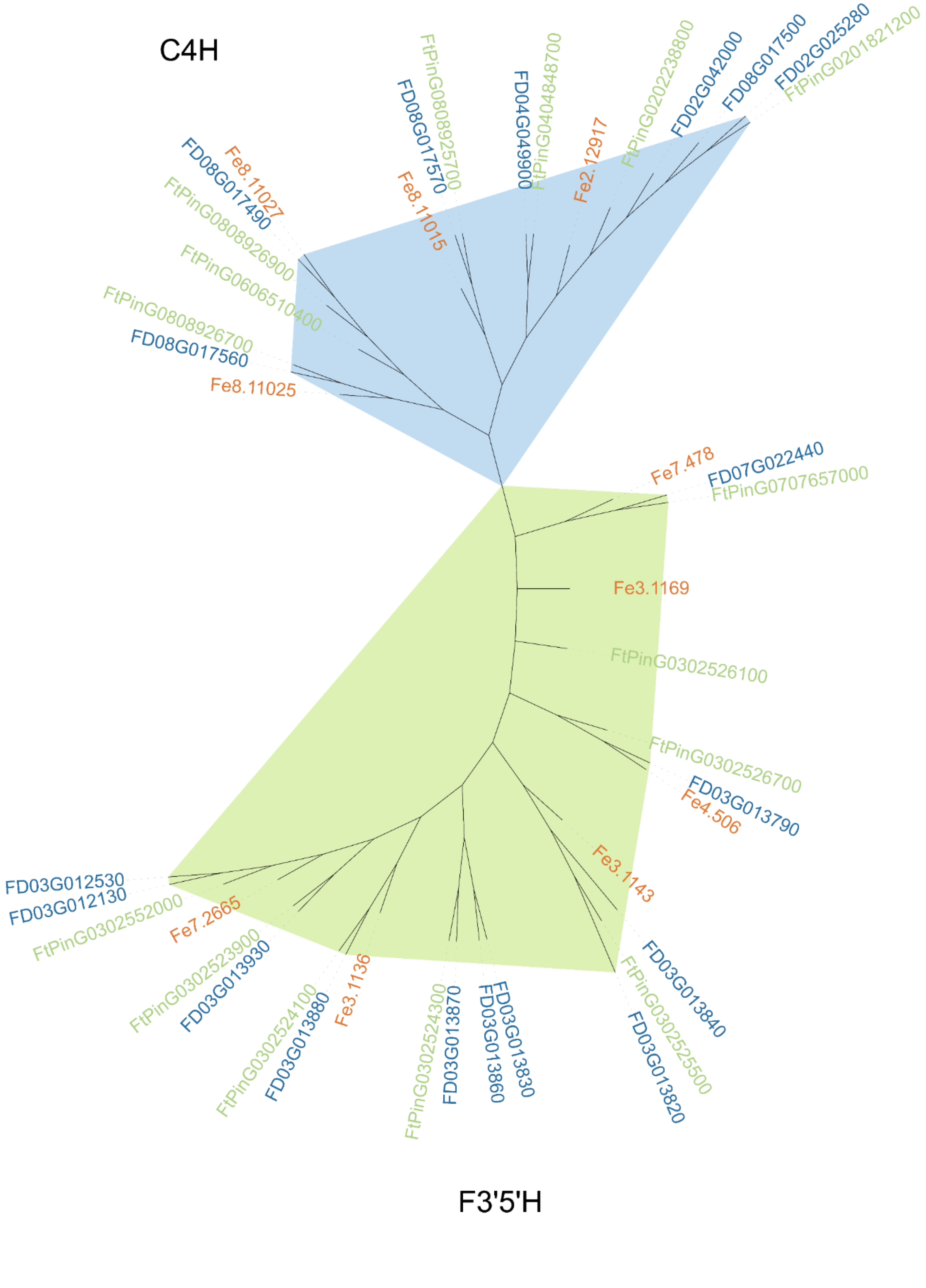

Fig. S7. Neighbor-joining tree of C4H and F3'5'H genes in *F. cymosum*, *F. esculentum*, and *F. tataricum*.

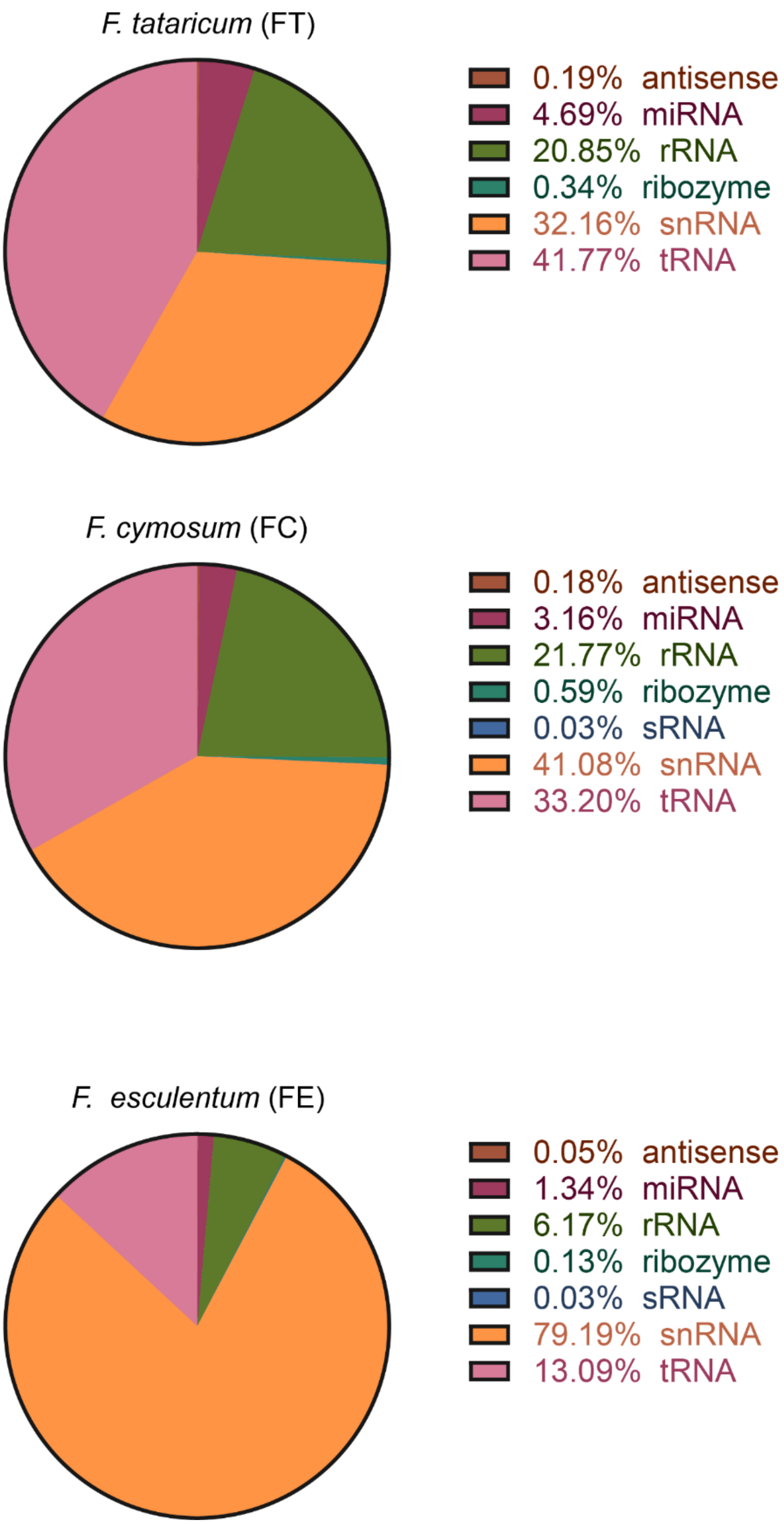

Fig. S8. Proportion of ncRNA in the buckwheat genomes.

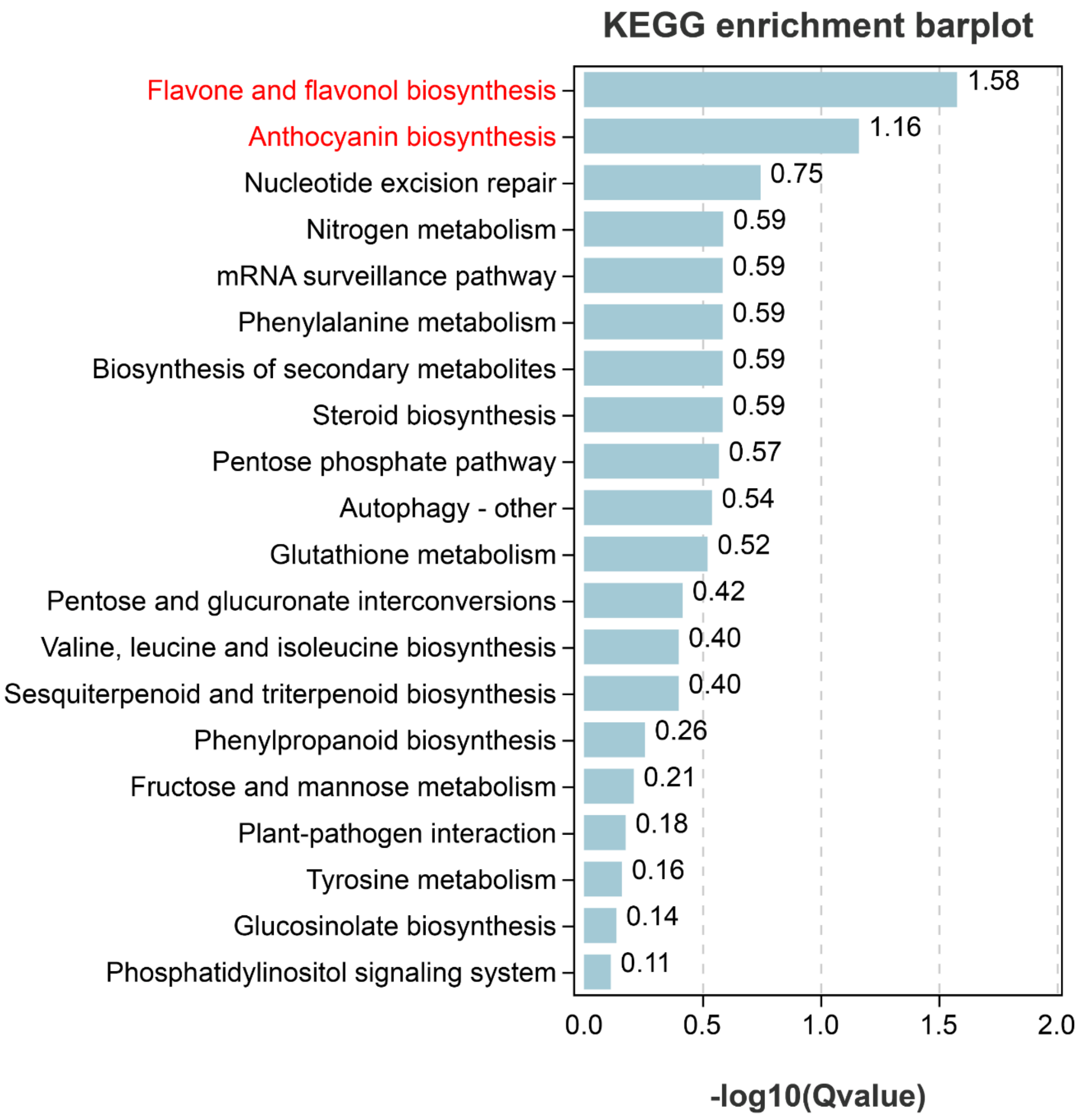

Fig. S9. KEGG enrichment results of genes in the *F. tataricum* inversion region.

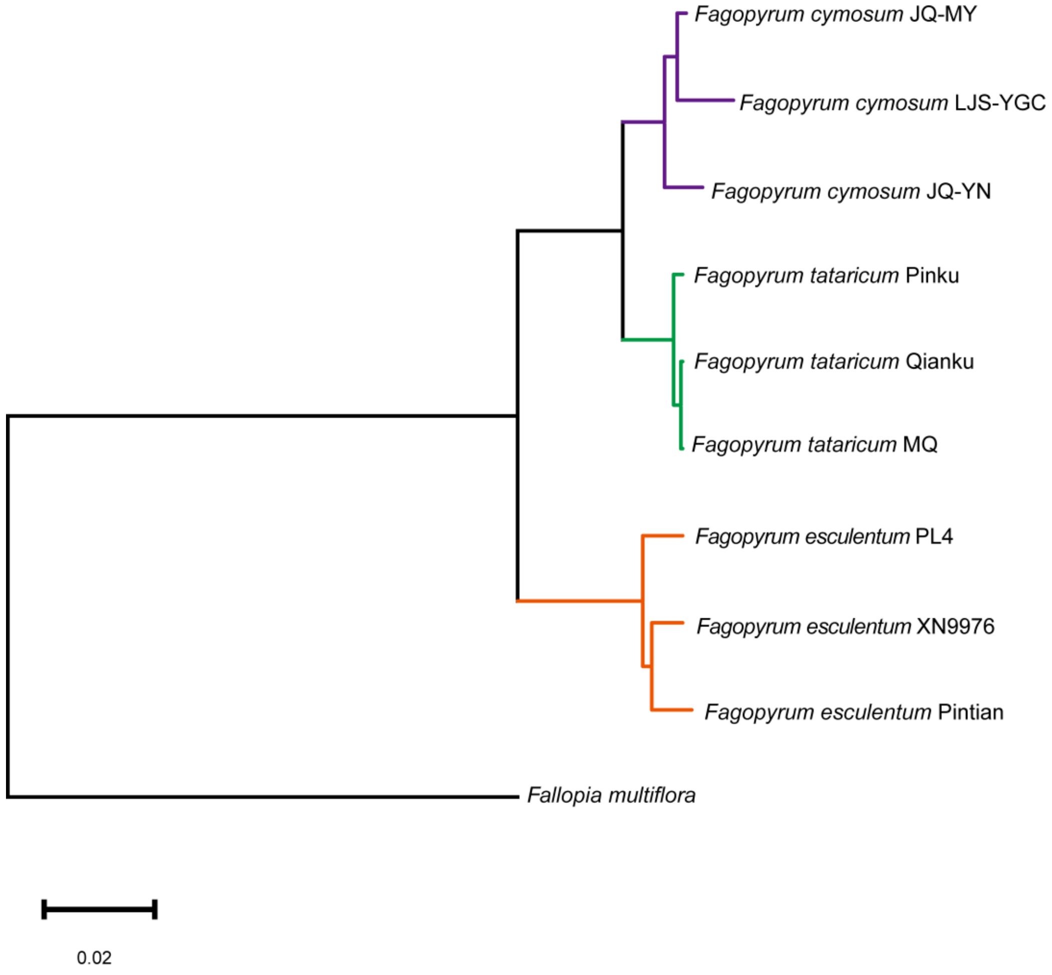

Fig. S10. A species tree constructed using the quadripartite test based on shared single-copy gene data from the three species. Each species had used the single copy genes from three genome assembly.

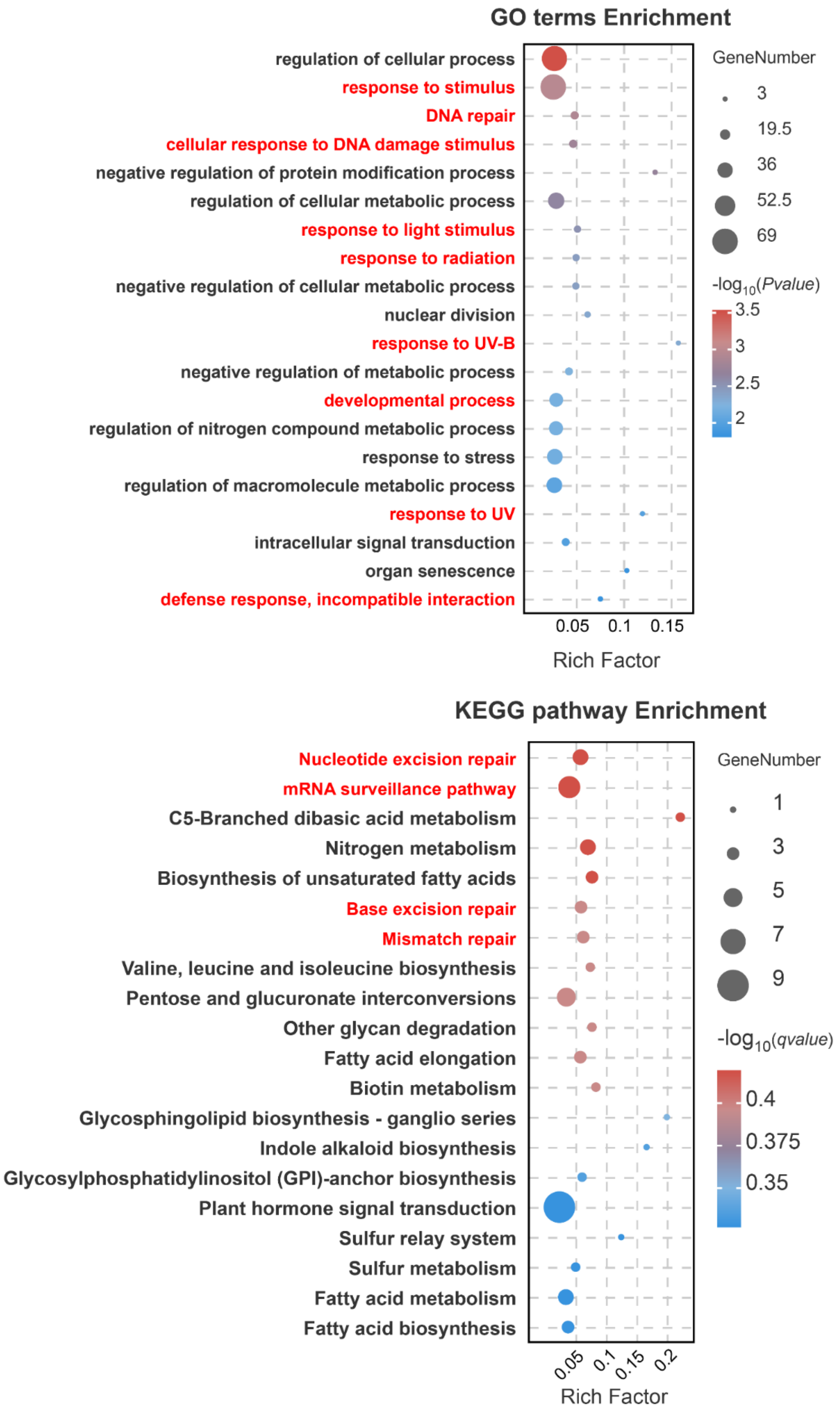

Fig. S11. Gene Ontology (GO) and Kyoto Encyclopedia of Genes and Genomes (KEGG) enrichment analysis of genes under positive selection in *F. tataricum*. Most of these genes are enriched in biological processes related to radiation damage resistance and metabolic pathways..

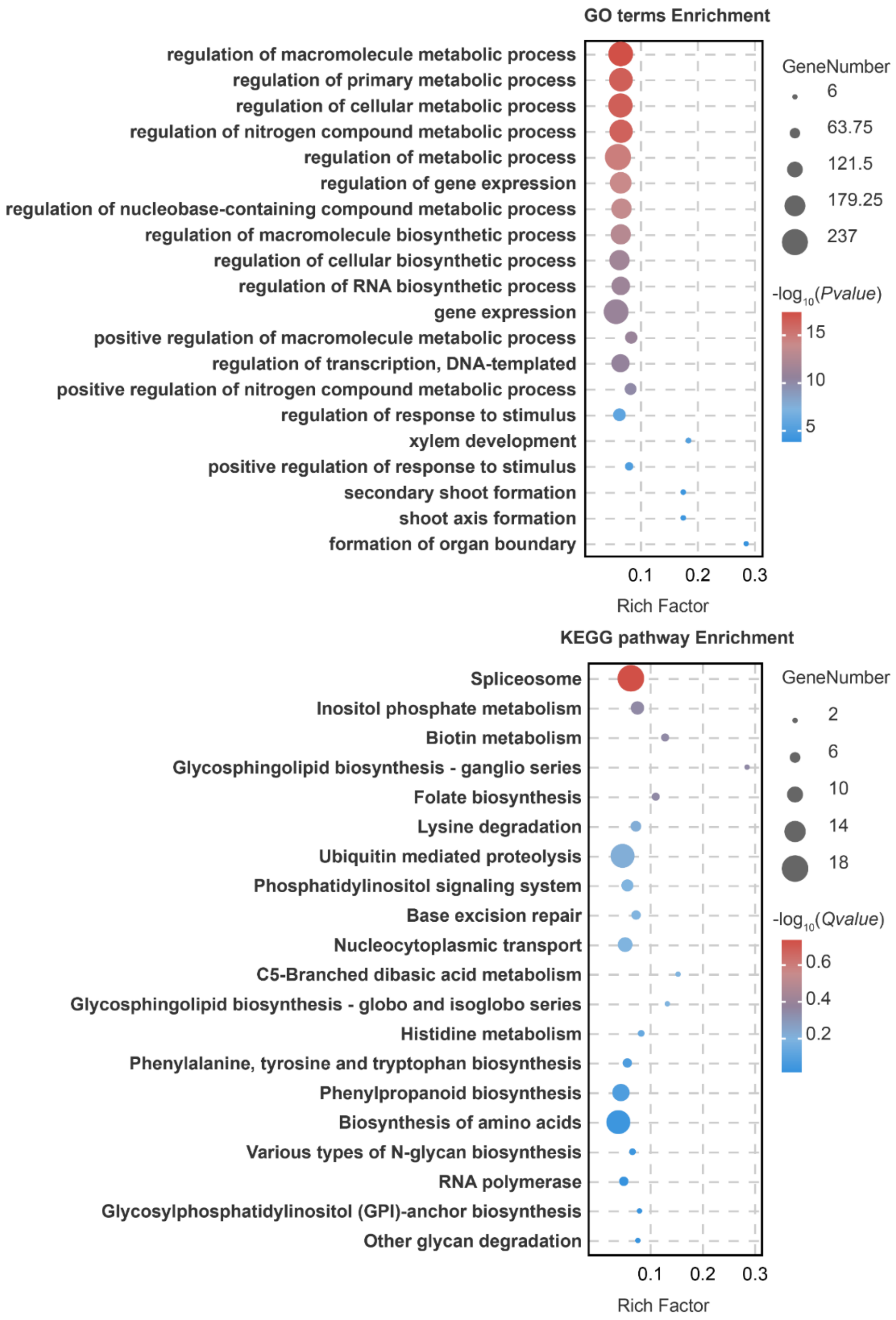

Fig. S12. Gene Ontology (GO) and Kyoto Encyclopedia of Genes and Genomes (KEGG) enrichment analysis of genes under positive selection in *F. esculentum.*

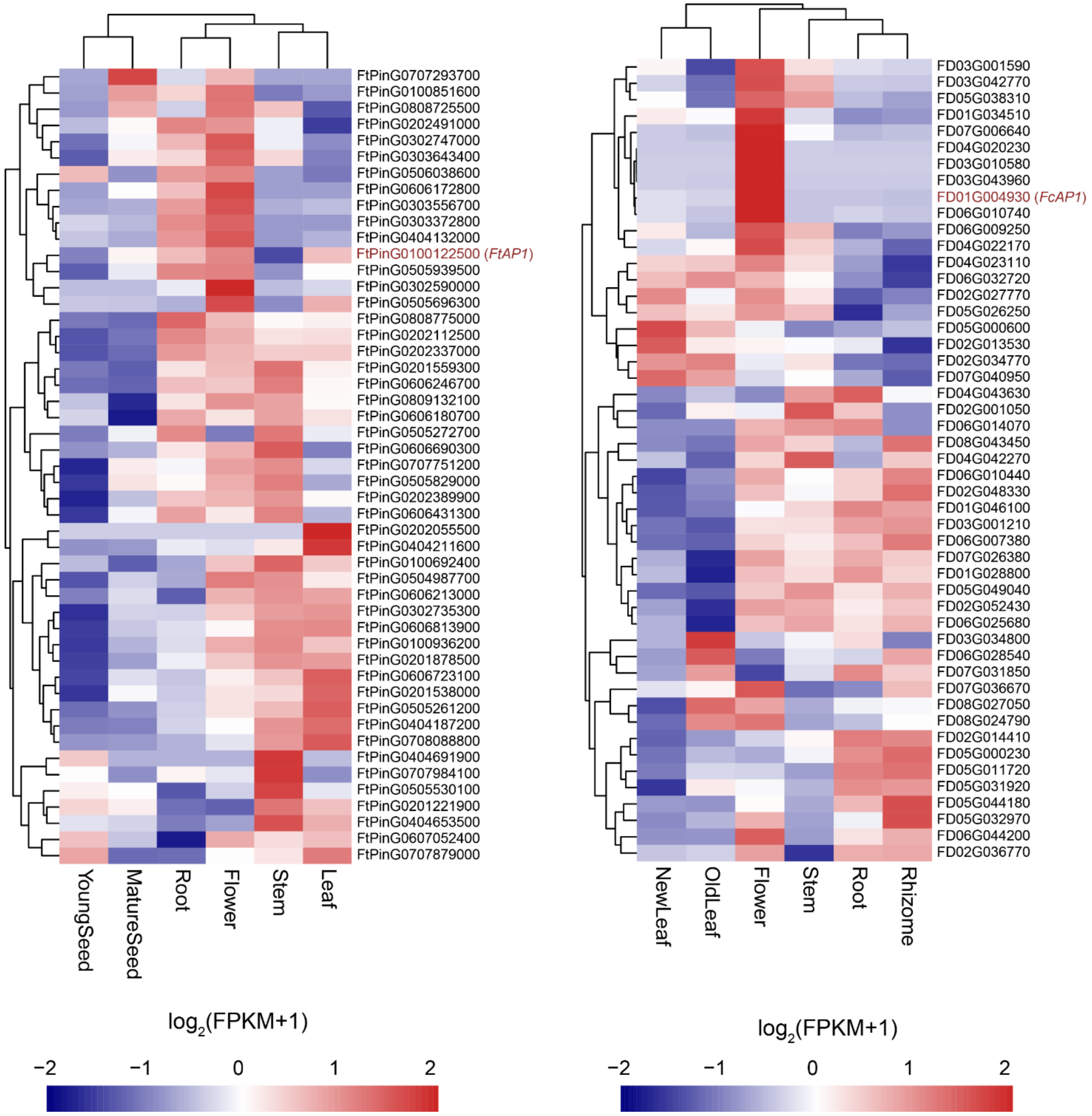

Fig. S13. The expression levels of flower development genes in different tissues of Tartary buckwheat and golden buckwheat affected by positive selection.

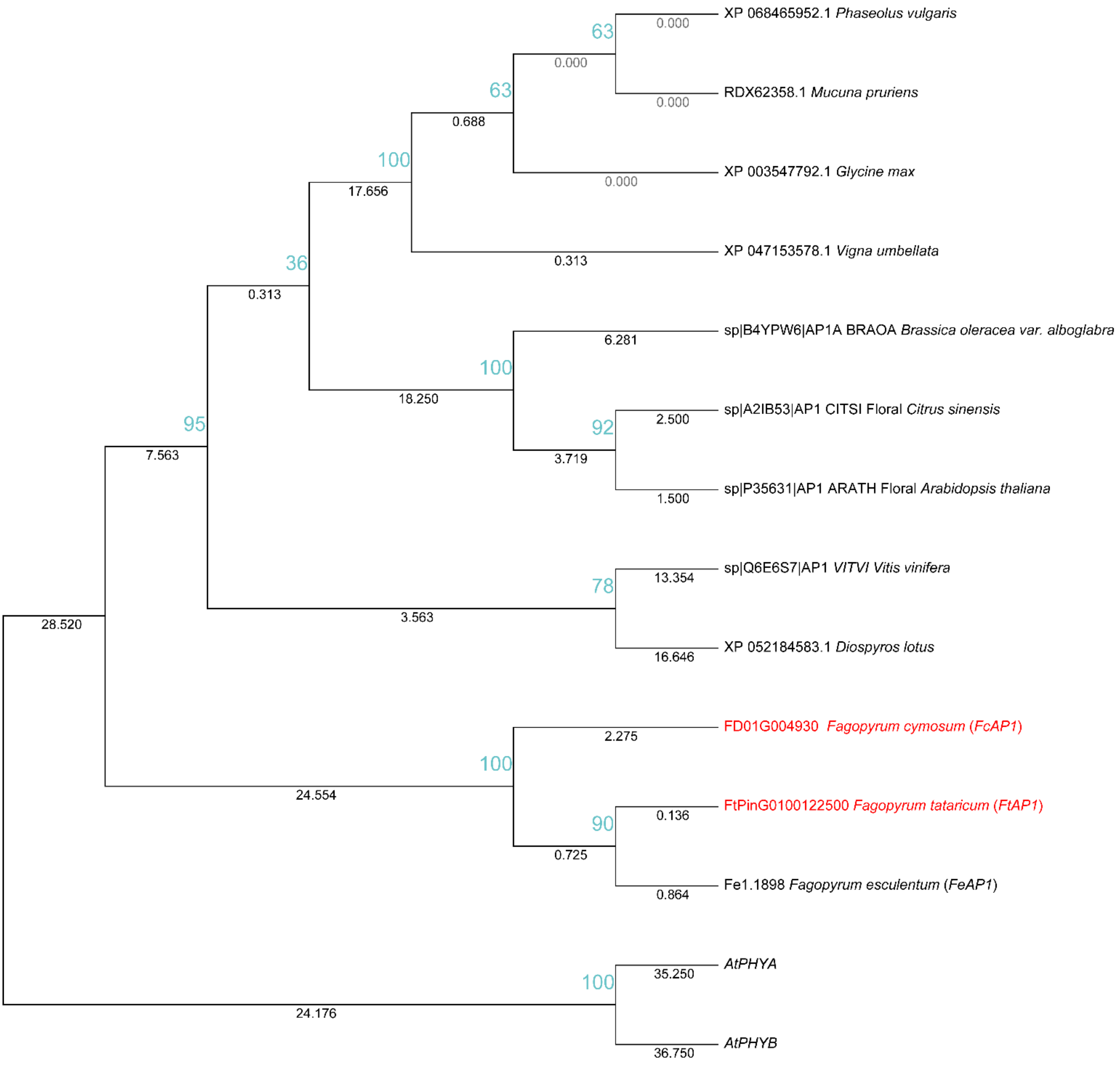

Fig. S14. Phylogenetic tree of the *AP1* gene constructed by the neighbor-joining method. The topology of the tree was built with default alignment and tree-building parameters, tested through 500 bootstrap iterations.

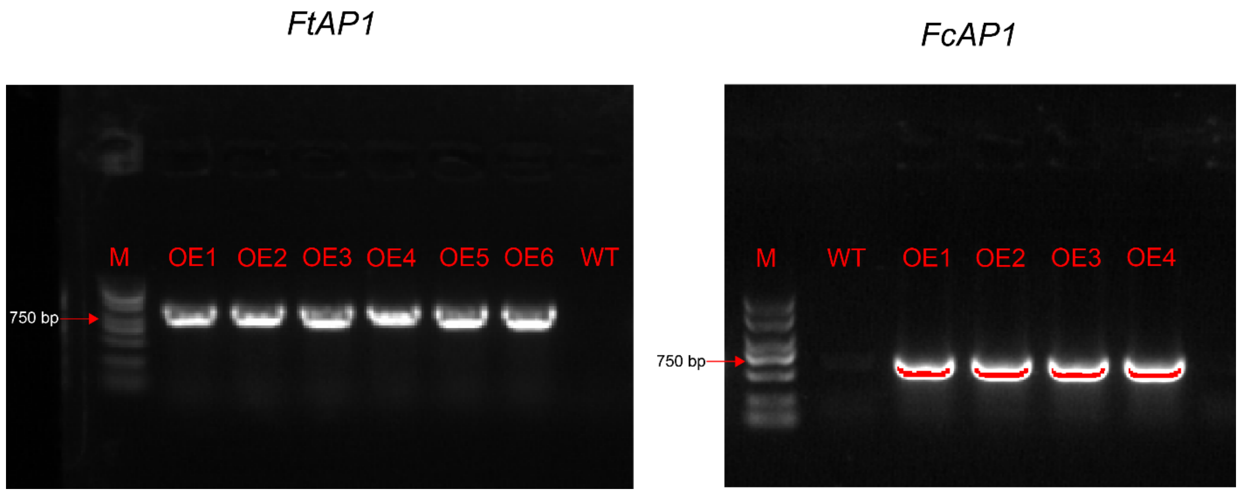

Fig. S15. PCR verification of overexpressed *FtAP1* and *FcAP1* transgenic lines in *A. thaliana*.

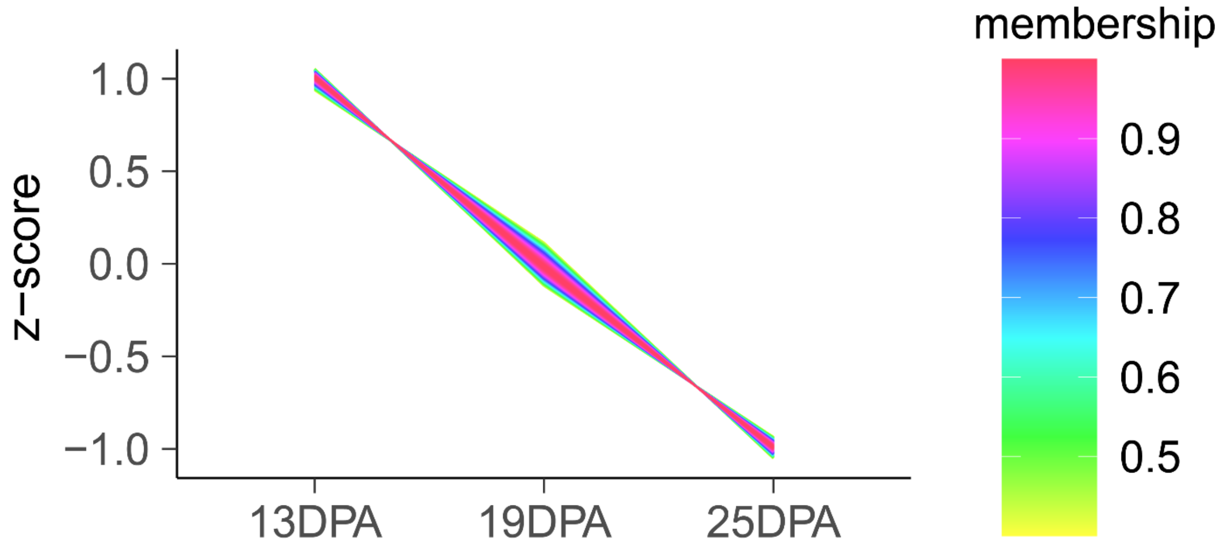

Fig. S16. Expression pattern clustering diagram. Clustering analysis of expression patterns across three different developmental stages of *F. tataricum* seeds was conducted. The results showed a similar declining trend in the expression of the *AP1* gene and its predicted interacting partner *SOC1* gene, beginning at the end of flowering.

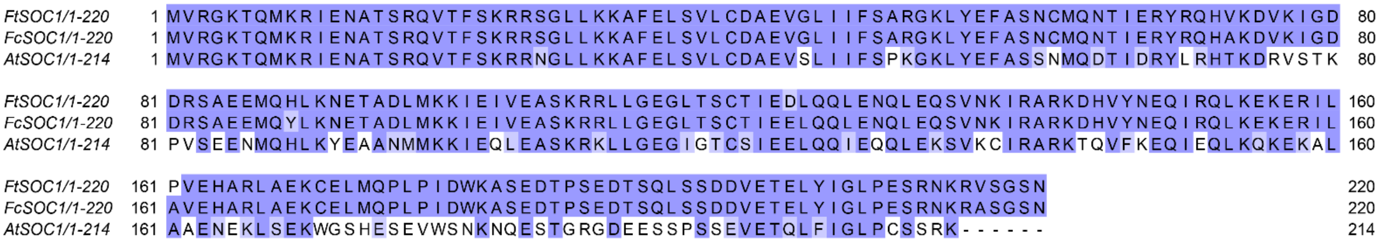

Fig. S17. Sequence alignment and conservation of SOC1. The SOC1 protein sequence was highly conservative in the *F. tataricum* and *F. cymosum*.

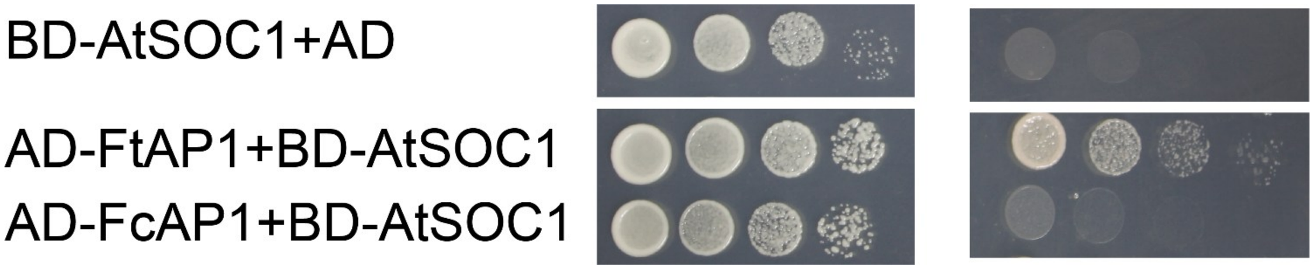

Fig. S18. Conservation of protein interactions in *Arabidopsis thaliana* validated by yeast two-hybrid Assay.

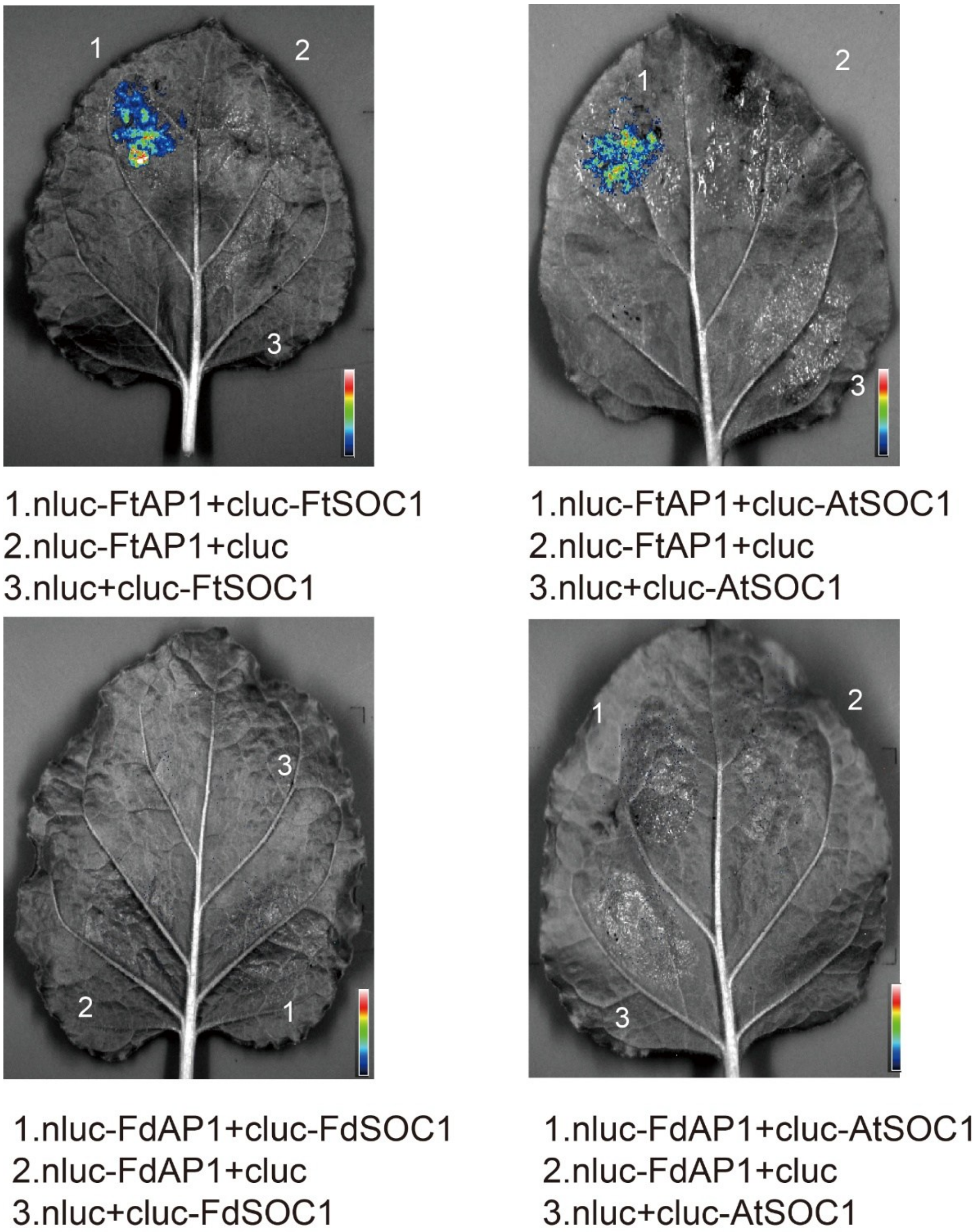

Fig. S19. The luciferase complementation assay (LCA) used to verify the conservation of protein interactions between AP1 and SOC1 in *A. thaliana* .

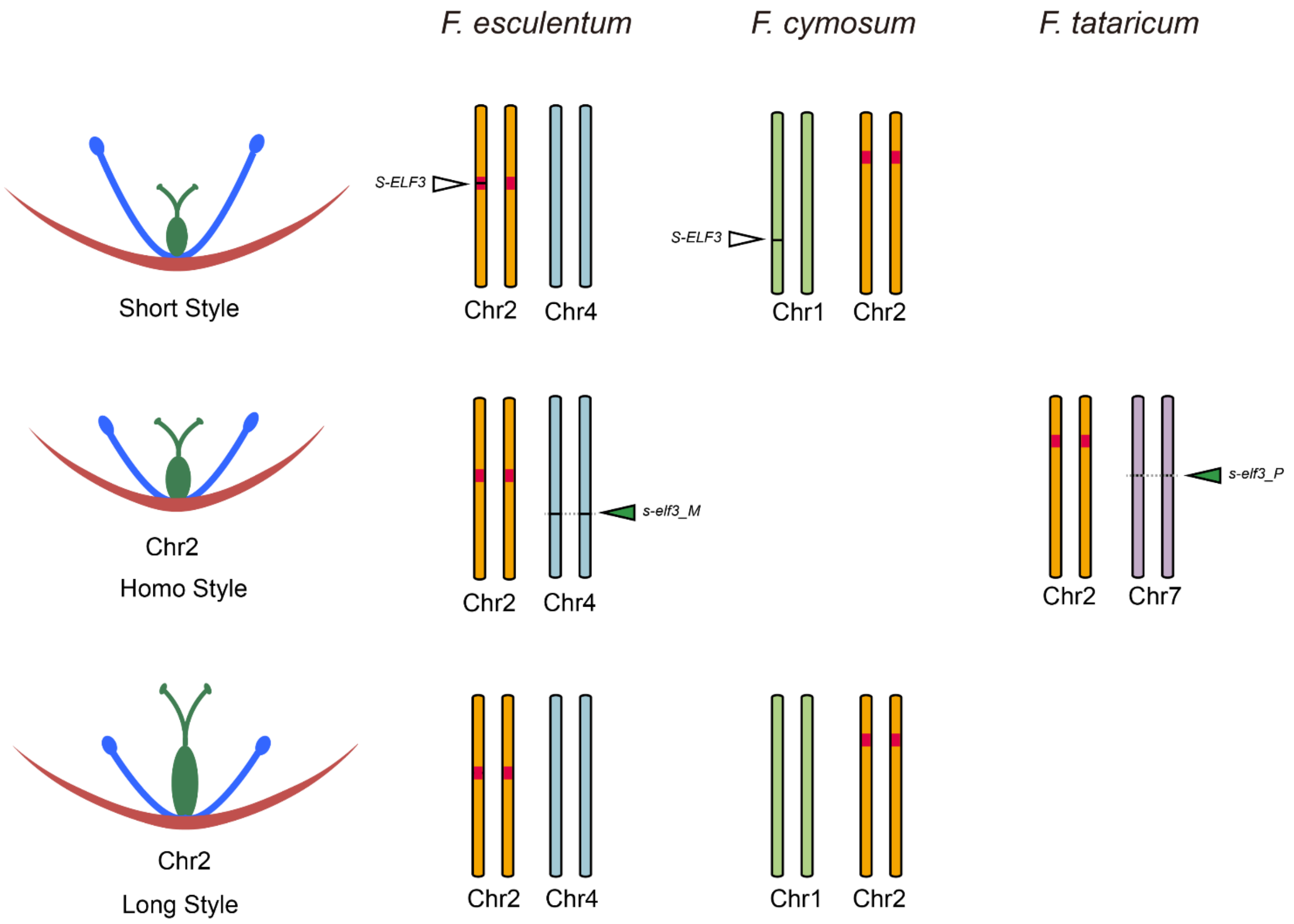

Fig. S20. Genomic mapping of putative loci associated with flower morphology and self-incompatibility.

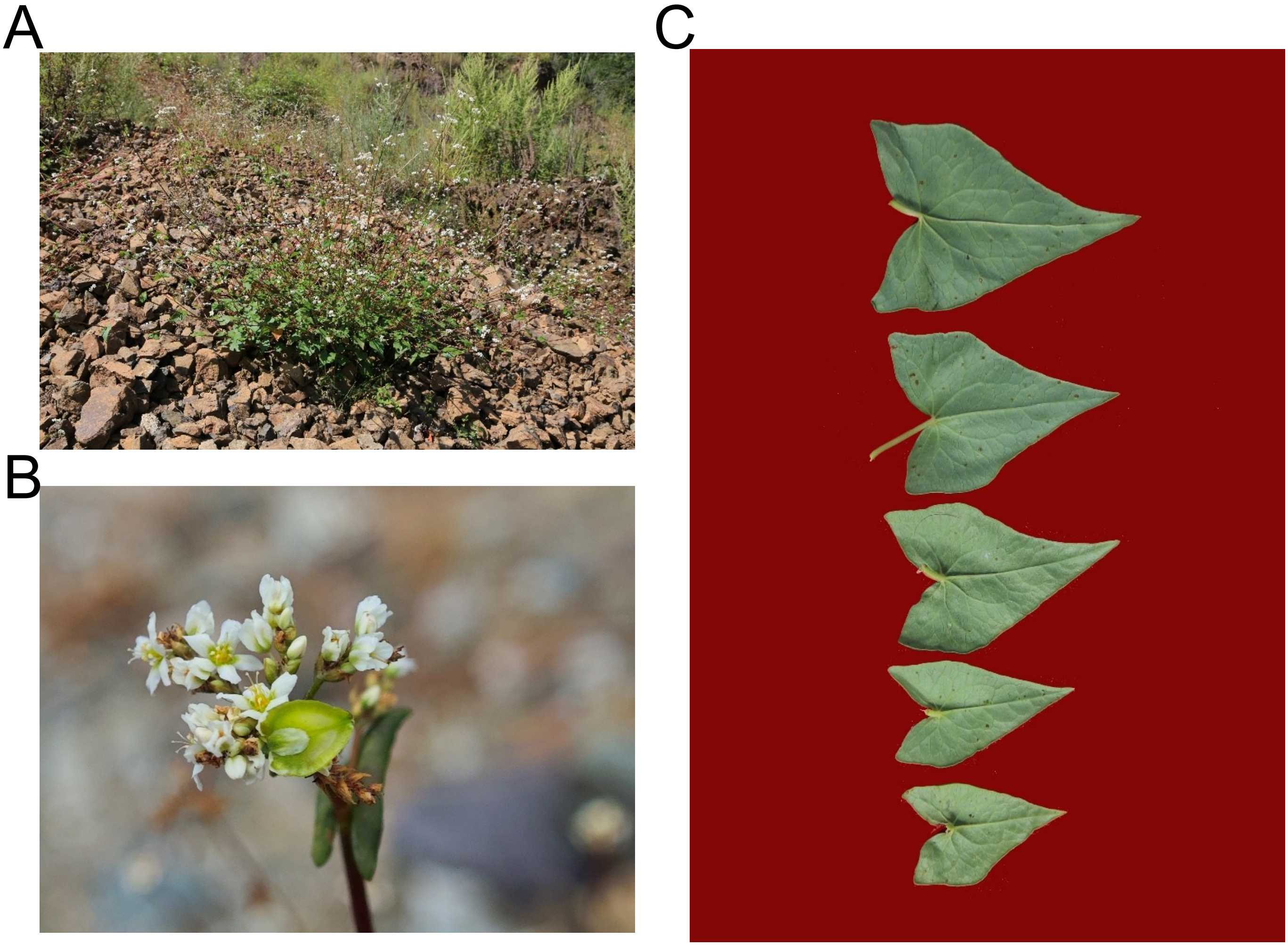

Fig. S21. The phenotypic characteristics of *F. esculentum ssp. ancestrale* under natural conditions. **A** The whole plant. **B** Flowers and seeds. **C** Leaves. Compared to cultivated common buckwheat, the *F. esculentum ssp. ancestrale* exhibited narrower leaves.

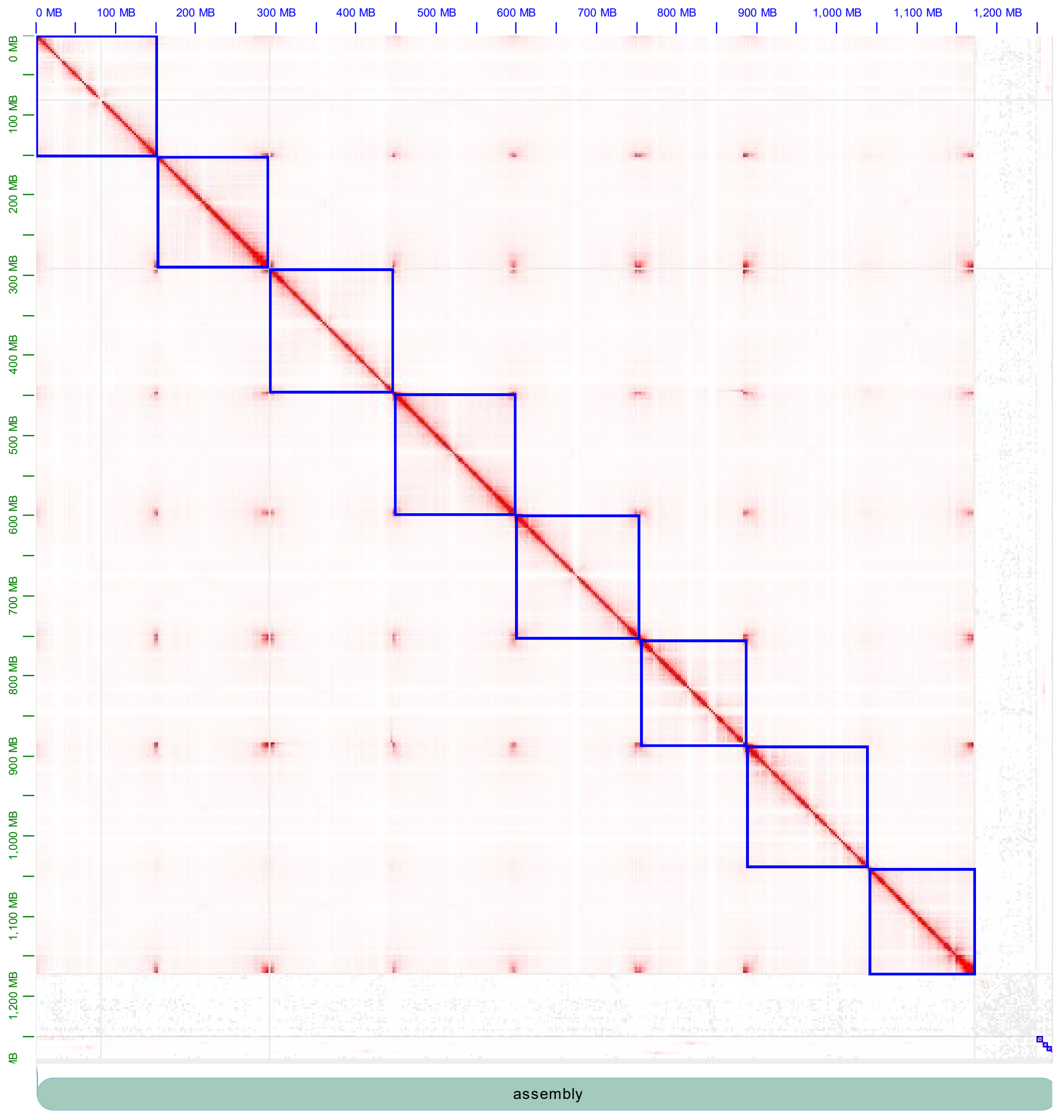

Fig. S22. Hi-C contact map of DDX genome assembly. The color intensity from white (low) to red (high) represents the frequency of chromatin interactions. Dark blue boxes delineate the boundaries of eight chromosomes.

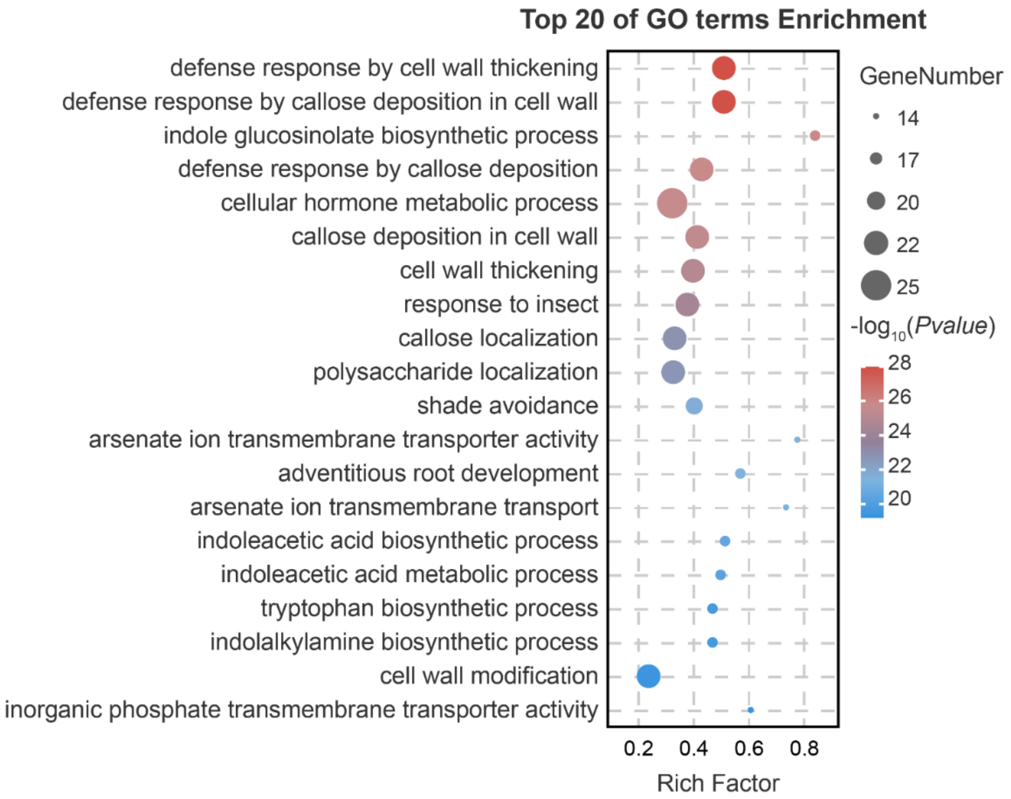

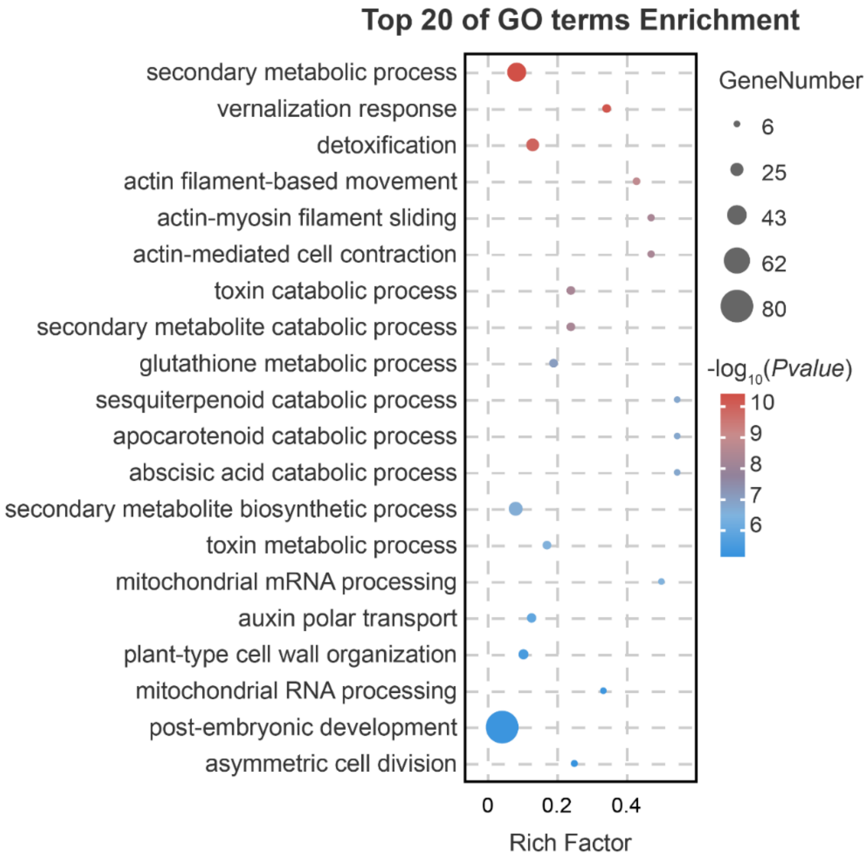

Fig. S23. GO enrichment analysis of expanded (top) and contracted (bottom) family genes in *F. esculentum* ssp. *Ancestrale* (DDX genome).

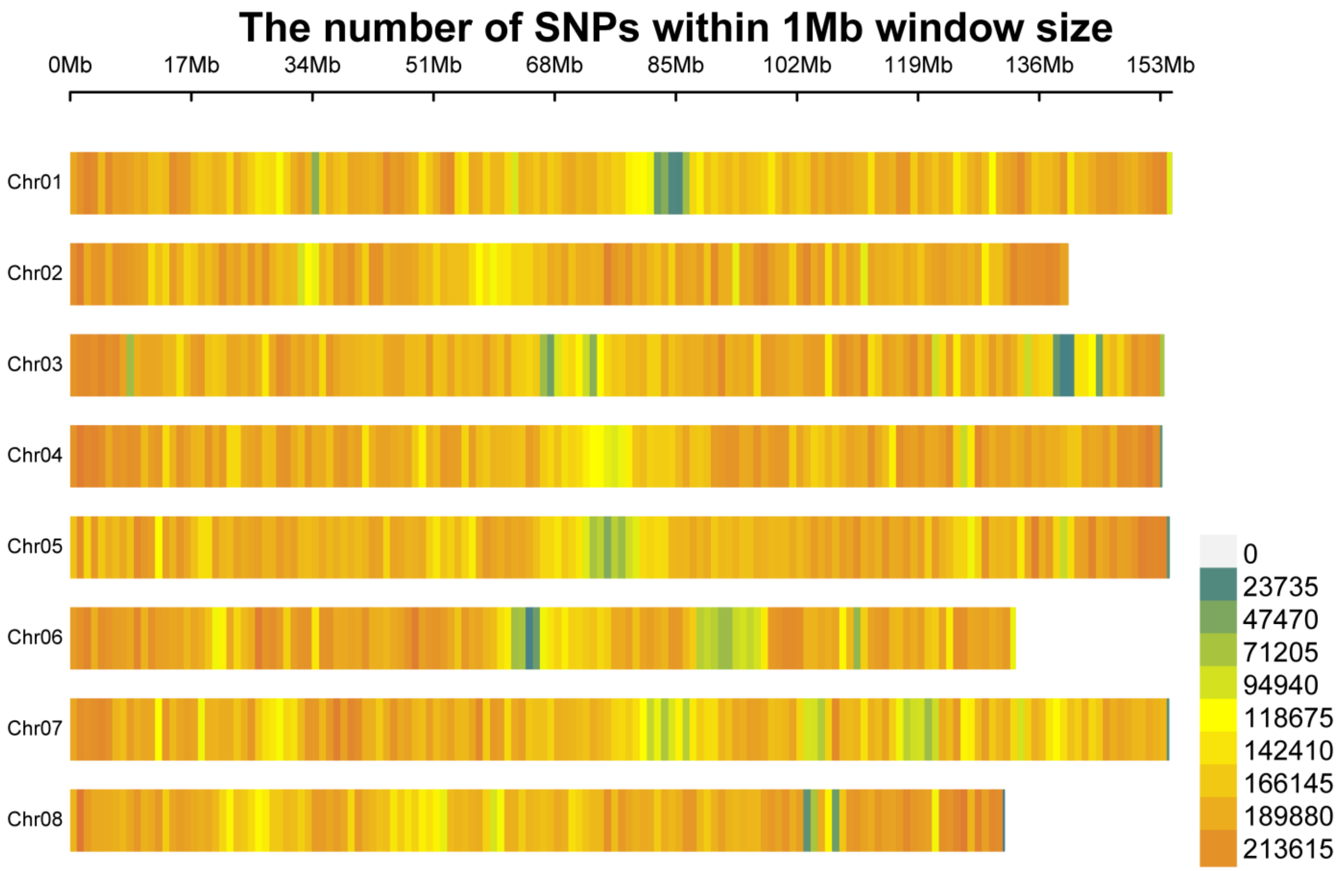

Fig. S24. Density plot of SNPs used for population analysis (the DDX genome as the reference genome).

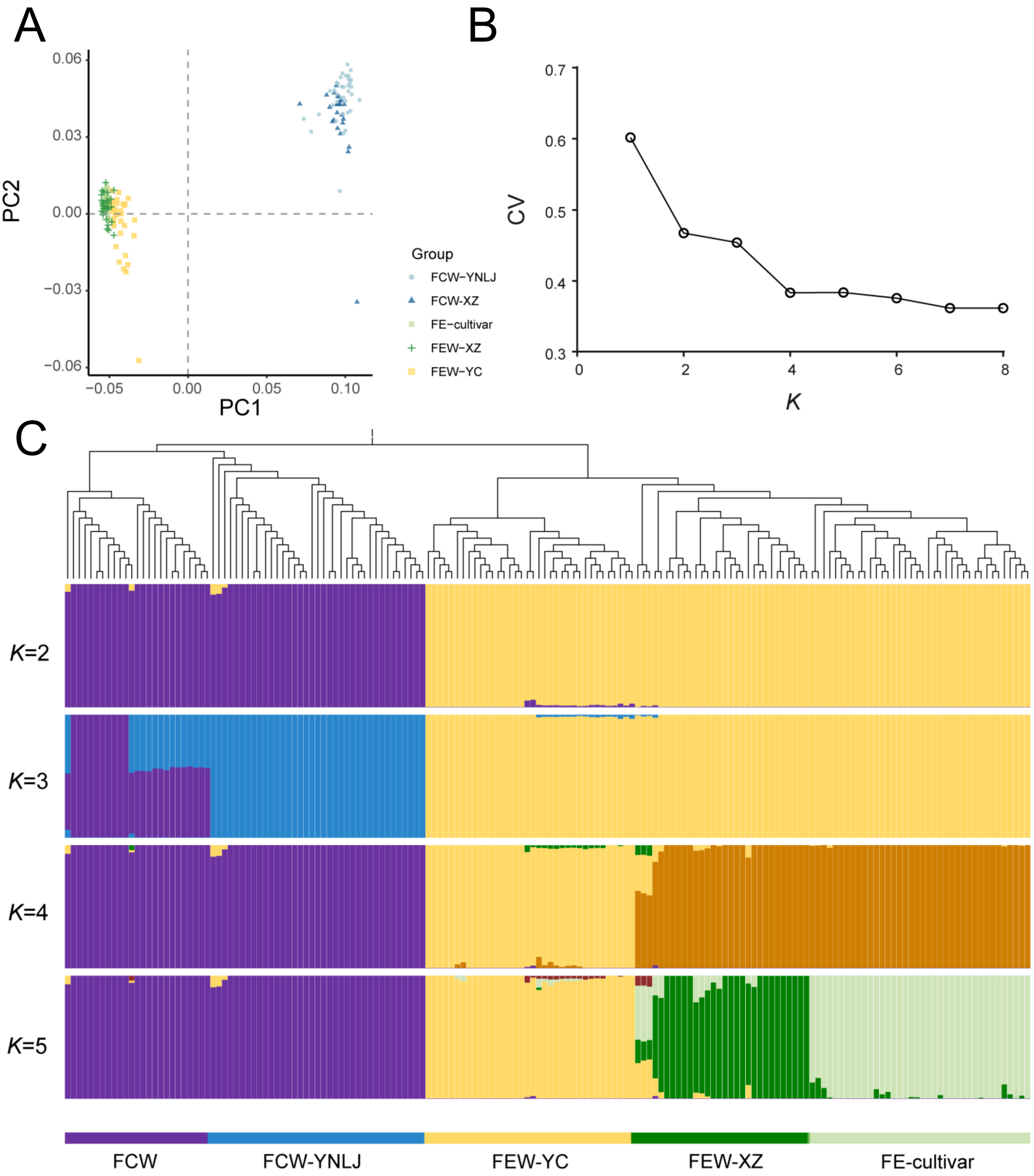

Fig. S25. Population structure of *Fagopyrum cymosum* and *Fagopyrum esculentum* (wild and cultivar). A PCA plot of five group. **B** Cross-Validation (CV) error curve of ADMIXTURE analysis. **C** Population structure of five interspecific groups.

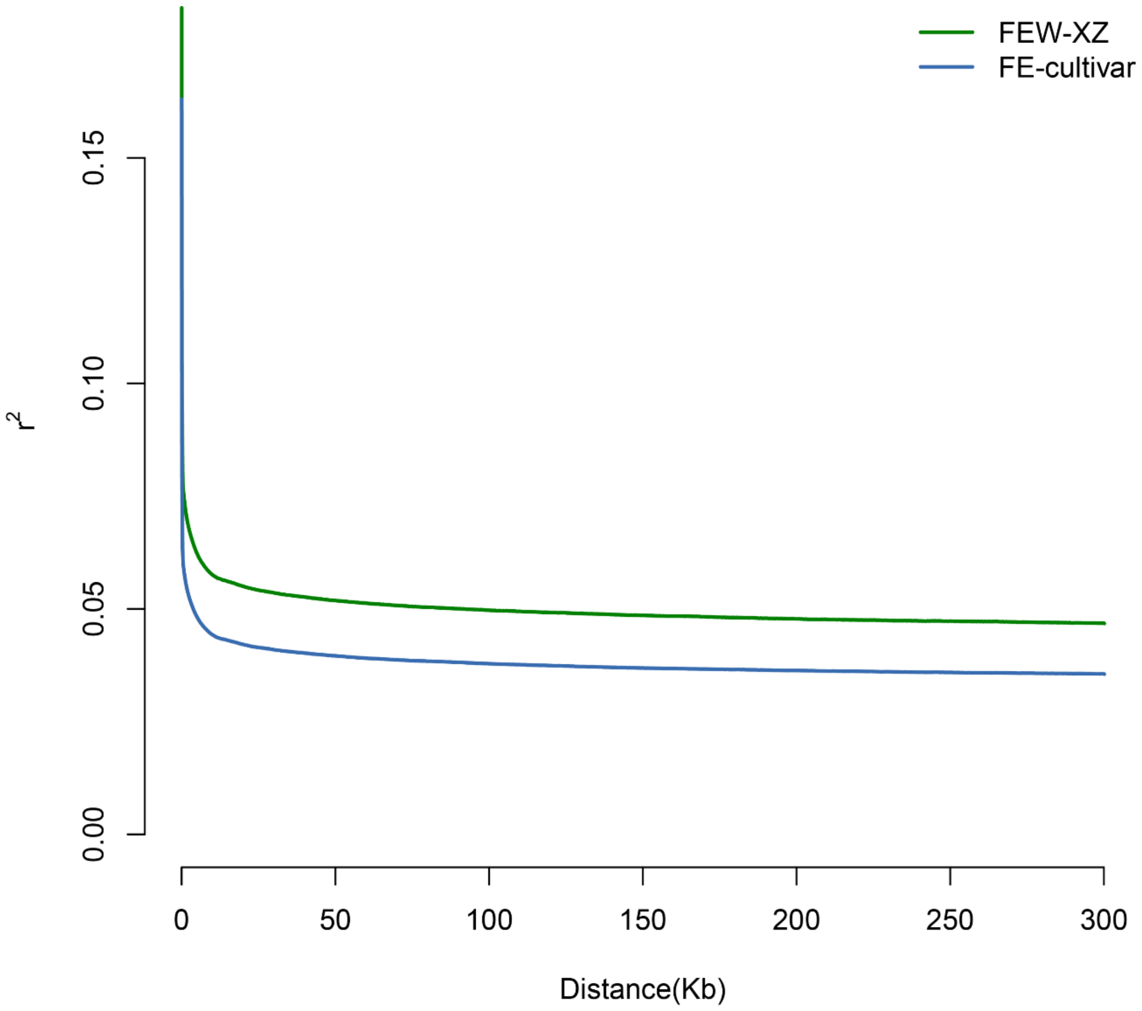

Fig. S26. LD decay curve of two groups of *Fagopyrum esculentum*.

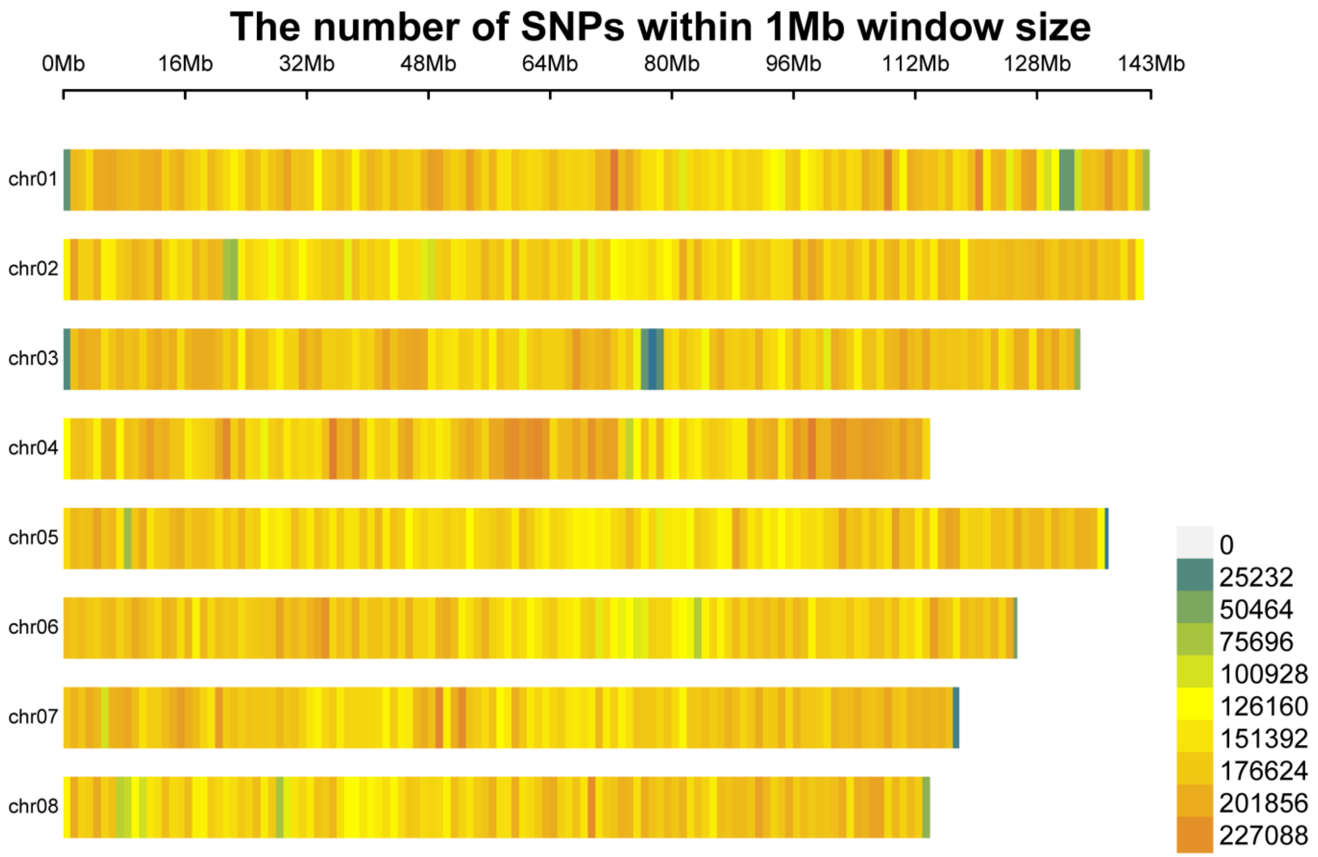

Fig. S27. Whole genome SNP density in the JQ-MY reference genome.

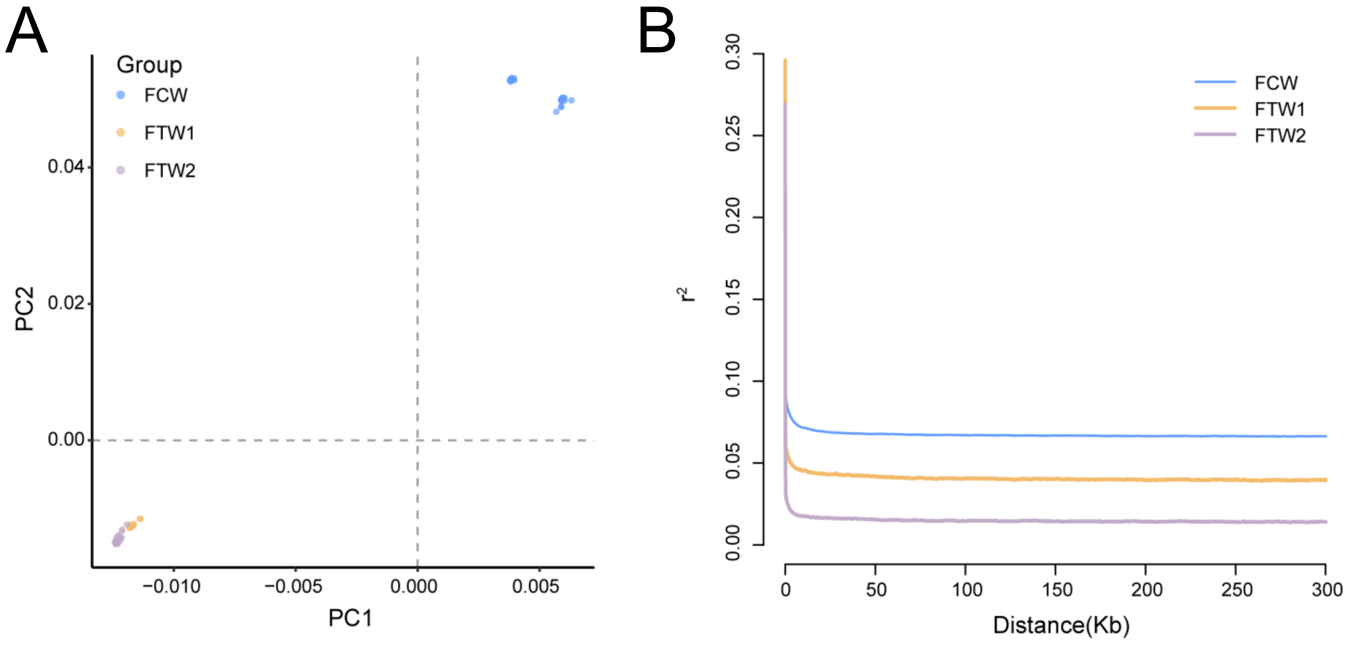

Fig. S28. PCA plot and LD decay curve of the three groups. A PCA plot of groups for *F. cymosum* and wild *F. tataricum*. **B** LD decay curve of groups for *F. cymosum* groups and two wild *F. tataricum* groups.

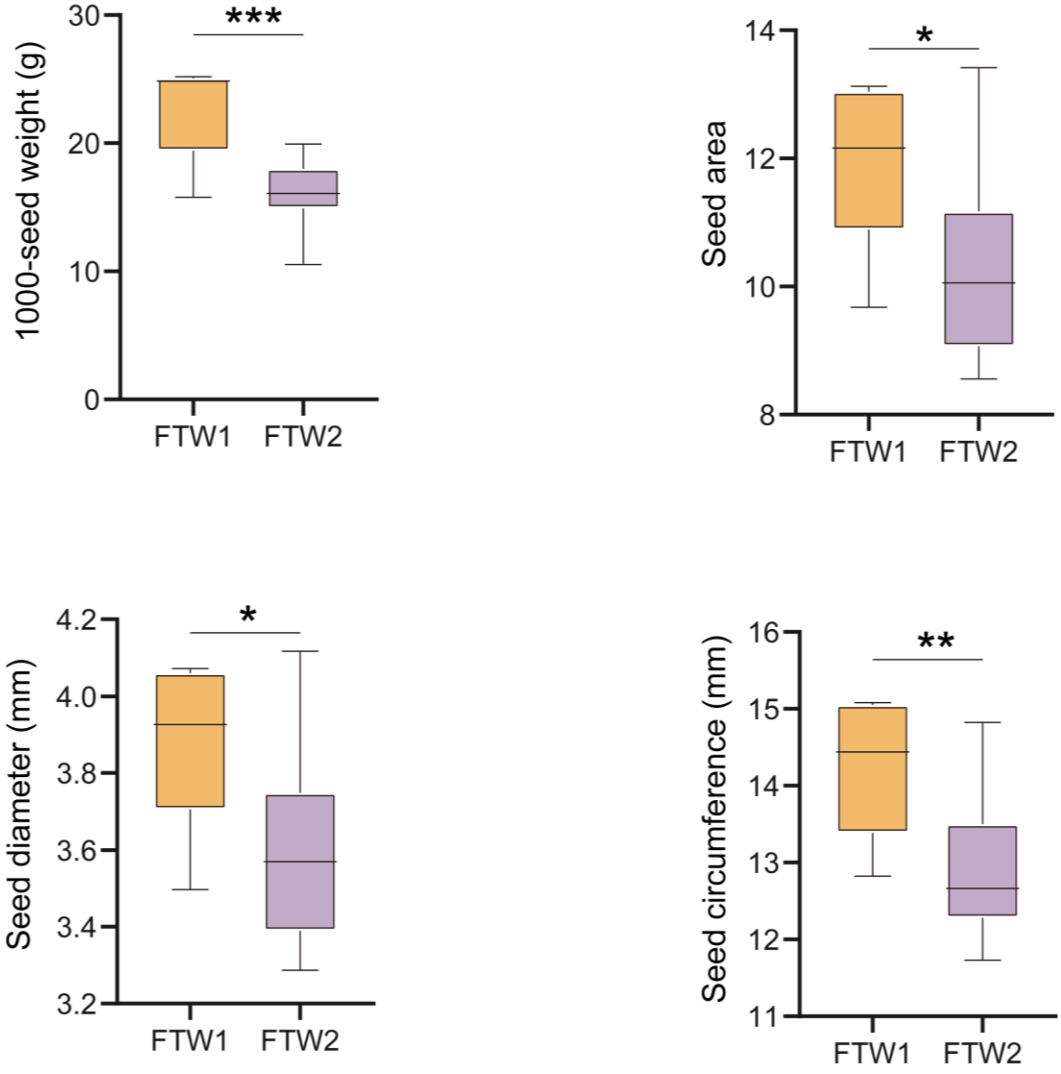

Fig. S29. Phenotypic identification of wild *F. tataricum* seeds. *: $p < 0.05$, **: $p < 0.01$, ***: $p < 0.001$.

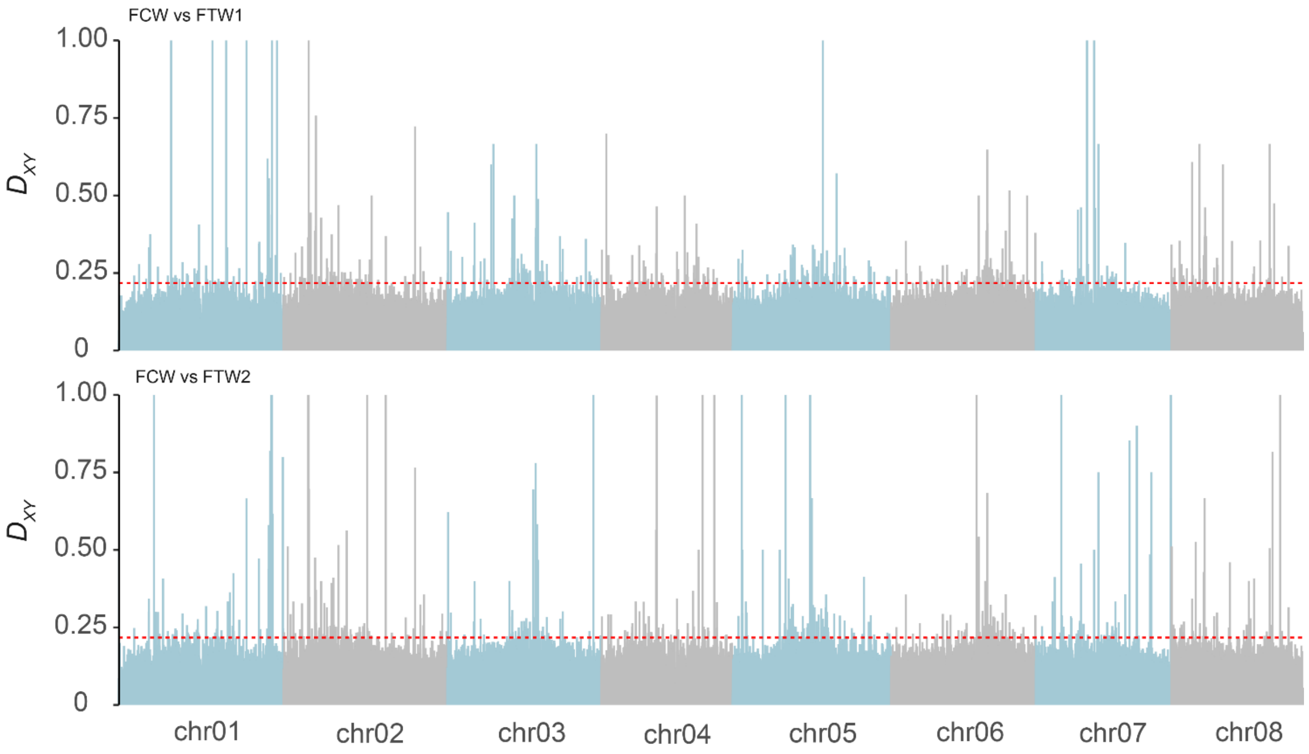

Fig. S30. Genetic differentiation between *F. cymosum* and *F. tataricum* (FTW1 and FTW2). Compared to the two wild buckwheat groups, Tartary buckwheat exhibited genetic differences in different regions of the genome, including both identical and distinct region (d_{xy}=1), as well as differences of varying scales in others region.

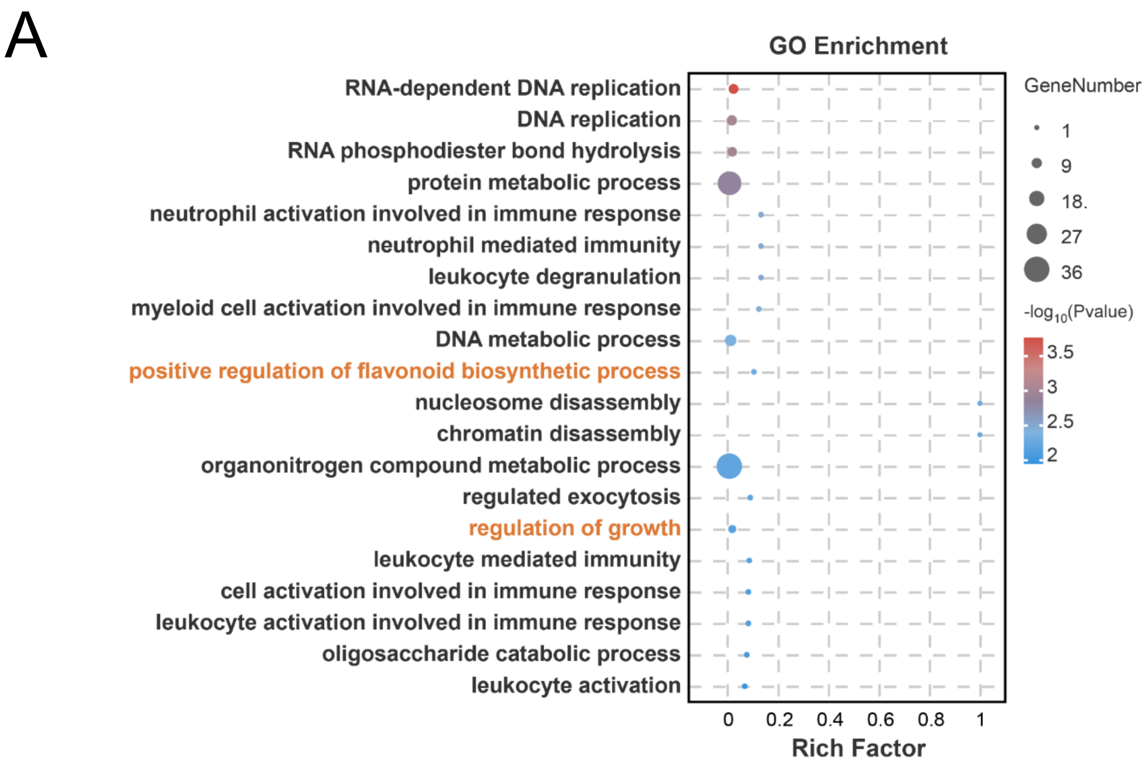

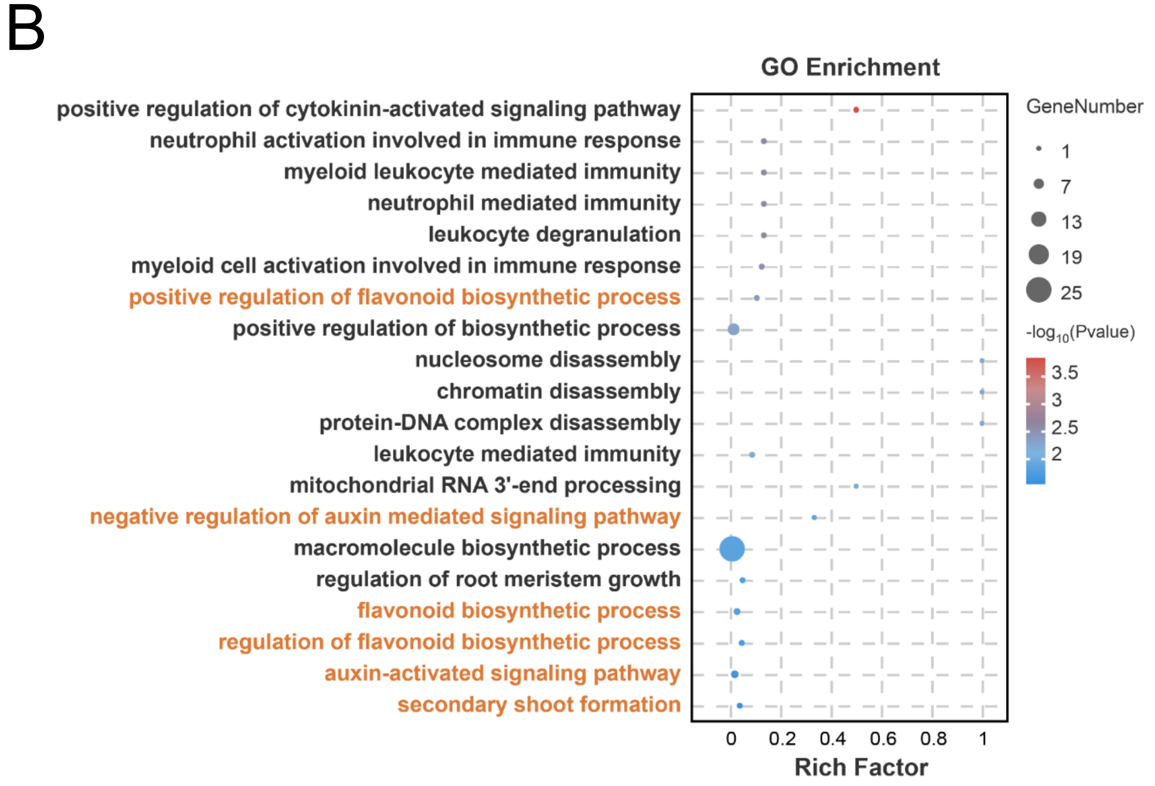

Fig. S31. GO enrichment analysis of gene differentiation between *F. cymosum* and *F. tataricum*. A Enrichment of genes in the differentiation region between FCW and FTW1. **B** Enrichment of genes in the differentiation region between FCW and FTW2.

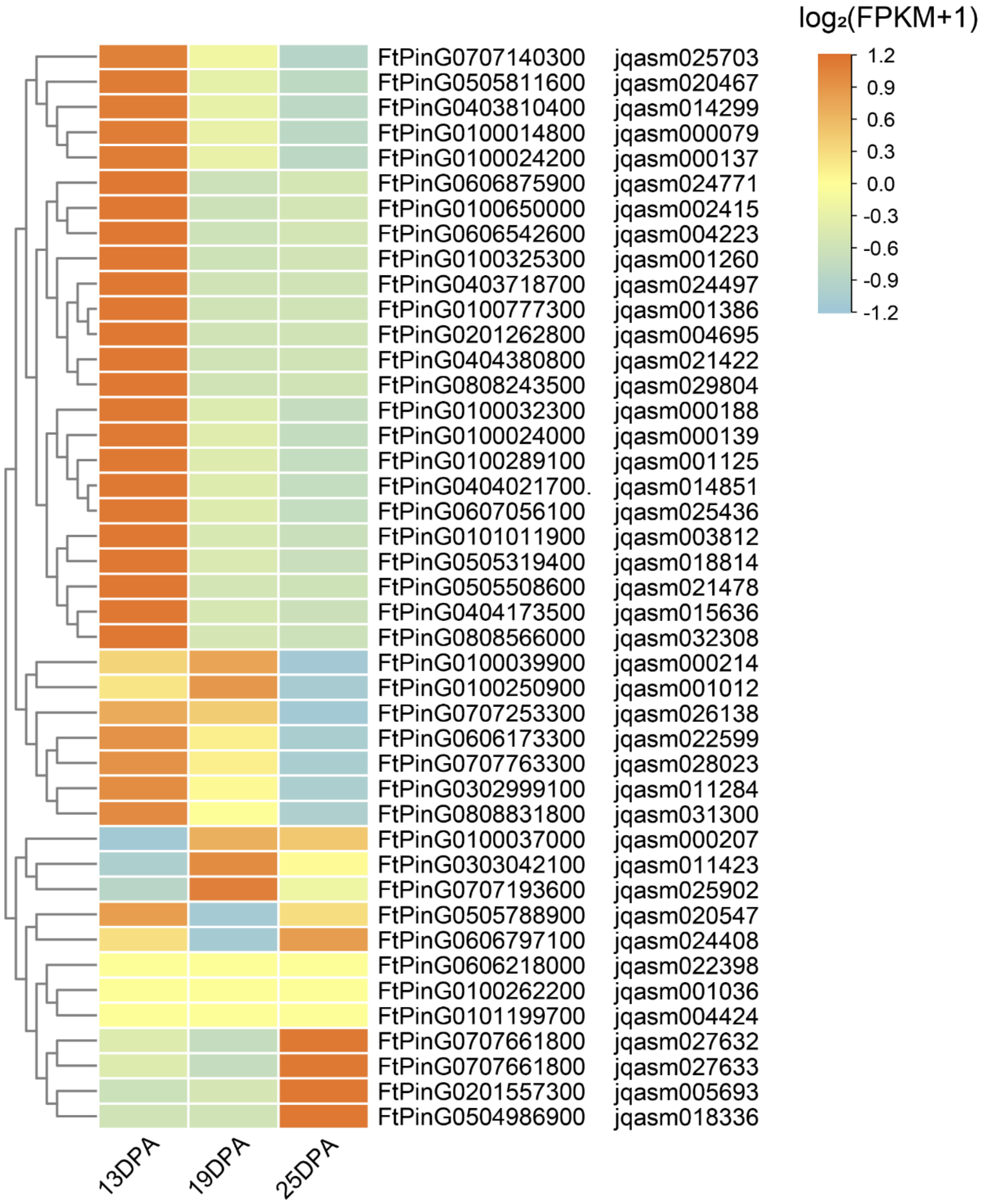

Fig. S32. Gene expression patterns of introgressed genes in seed development. DPA: the seed development stage at 13, 19, and 25 days after flowering. The first column of gene name was referred to as the *F. tataricum,* and last column was the orthologous gene in *F. cymosum*.

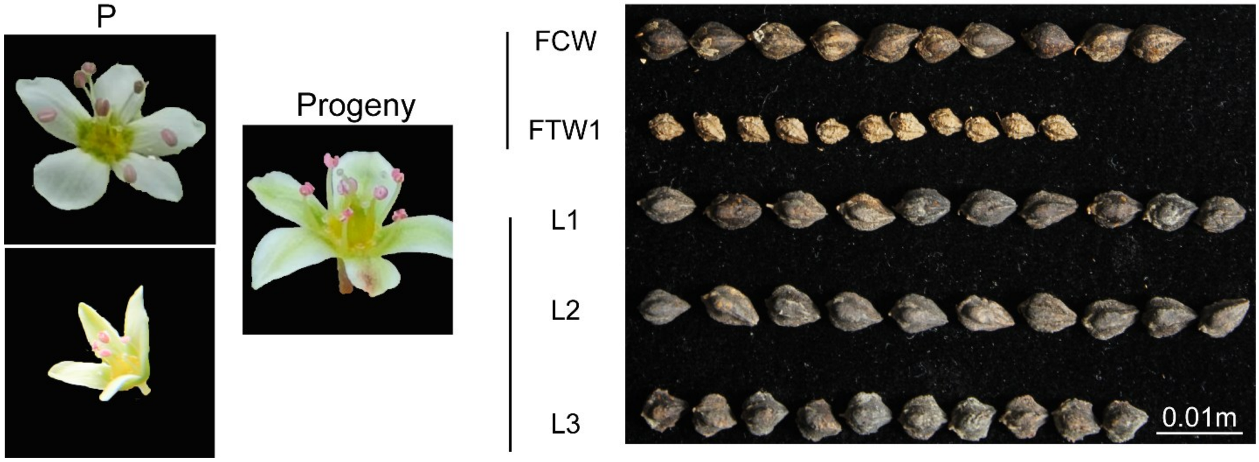

Fig. S33. The hybrids progeny between wild *F. cymosum* and wild *F. tataricum* in the FTW1 group. These progenies exhibit floral and seed morphological traits similar to wild *F. cymosum*.

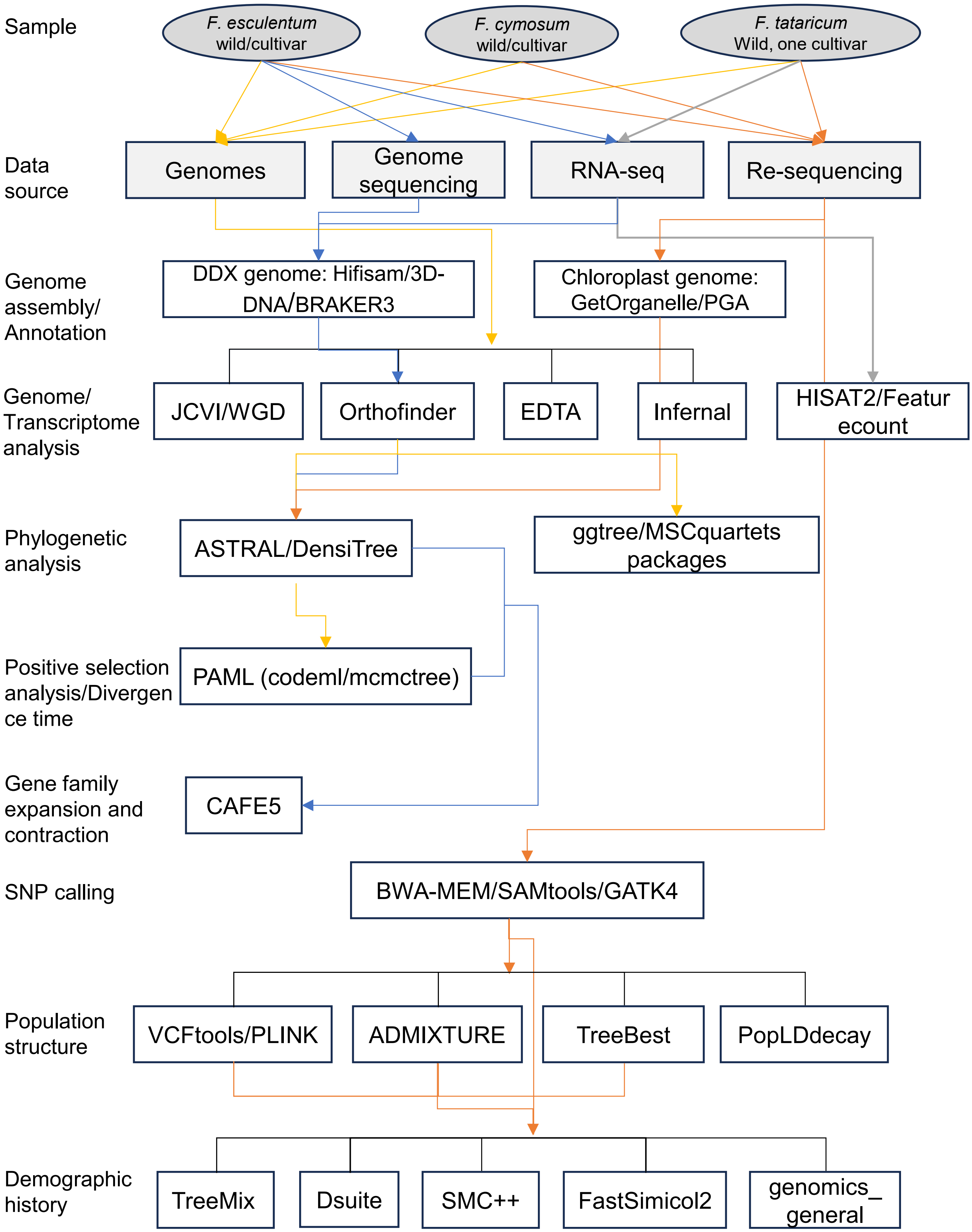

Fig. S34. The flow chart of data processing in this study. The processing purposes were showed in the left of figure. Software and programs were deposited in the rectangular frame. Lines with green, blue, gray, and orange color indicated the flow of sourced data.
